# Curcuminoid–BF_2_ complexes: Synthesis, fluorescence and optimization of BF_2_ group cleavage

**DOI:** 10.3762/bjoc.13.223

**Published:** 2017-10-26

**Authors:** Henning Weiss, Jeannine Reichel, Helmar Görls, Kilian Rolf Anton Schneider, Mathias Micheel, Michael Pröhl, Michael Gottschaldt, Benjamin Dietzek, Wolfgang Weigand

**Affiliations:** 1 Institute for Inorganic and Analytical Chemistry, Friedrich-Schiller-Universität Jena, Humboldtstrasse 8, 07743 Jena, Germany; 2 Institute for Physical Chemistry, Friedrich-Schiller-Universität Jena, Helmholtzweg 4, 07743 Jena, Germany; 3 Leibniz Institute of Photonic Technology (IPHT), Albert-Einstein-Straße 9, 07745 Jena, Germany; 4 Jena Center of Soft Matter, Friedrich-Schiller-Universität Jena, Philosophenweg 7, 07743 Jena, Germany

**Keywords:** BF_2_ complex, curcumin, dyes, fluorescence, hydrolysis, spectroscopy

## Abstract

Eight difluoroboron complexes of curcumin derivatives carrying alkyne groups containing substituents have been synthesized following an optimised reaction pathway. The complexes were received in yields up to 98% and high purities. Their properties as fluorescent dyes have been investigated. Furthermore, a strategy for the hydrolysis of the BF_2_ group has been established using aqueous methanol and sodium hydroxide or triethylamine.

## Introduction

In recent years curcumin, a pigment naturally occurring in *curcuma longa*, and its analogues, the curcuminoids, have attracted much attention regarding their biological activities [1]. These include antioxidant [2] and radical scavenging [3], antitumor [4] and anti-inflammatory [5] activities, as well as HIV inhibition [6]. Despite showing activity against several diseases and having only negligible side effects, low water solubility and fast degradation limit their potential medical application to this day [7].

**Scheme 1:**
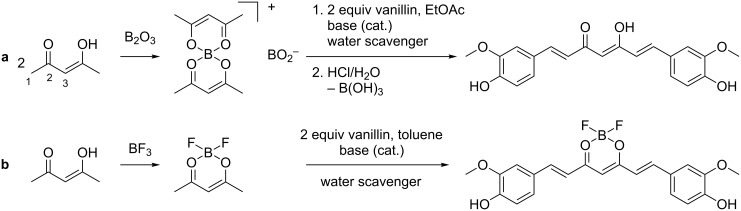
Synthesis of the curcumin structure motif using (a) boric oxide or (b) boron trifluoride.

The formation of the curcumin structure motif takes place by a base catalysed aldol condensation between the corresponding aldehyde and 2,4-pentanedione. To avoid a Knoevenagel condensation at the C-3 atom (Scheme 1a), the β-diketone moiety needs to be fixed into the enol form. In principle, this can be achieved by two different methods. The first one described by Pabon et al. utilises boric oxide in ethyl acetate as an intermediate agent (Scheme 1a) [8]. Boric acid esters like tri-*n*-butyl borate are normally used to scavenge water being produced during the reaction, while piperidine [9] and *n*-butylamine [10–11] are typical bases used as catalysts for this type of reaction (Scheme 1). Although working well with vanillin and similar derivatives, the yields strongly decrease when employing other aldehydes [12]. This procedure also requires a rather extensive work-up including several extraction steps and chromatography [8]. A second, more recent approach first published by Rao et al. relies on boron trifluoride as the complexing agent [13]. The reaction was altered by Zhang et al. to be carried out in toluene (Scheme 1b) [14]. This reaction produces the BF_2_ complex of the corresponding curcuminoid in yields up to 98% and high purity as an insoluble solid, which requires only a minimum of work-up.

The BF_2_ complexes by themselves have attracted attention regarding their properties as fluorescent dyes with fluorescence quantum yields of up to 60% and Stokes shifts of up to 5000 cm^−1^ [9]. Additionally, the incorporation of the BF_2_ group forces the β-diketone unit into the enol form, which leads to increased rigidity and enhanced photostability of the molecule [9].

Although a range of papers reports the synthesis of BF_2_ complexes of β-diketones such as curcuminoids [13–14] or dibenzoylmethanes [15–16], to our knowledge the reported procedures for the hydrolysis of these complexes are very limited and not always reproducible.

As part of our ongoing research to increase the selectivity of antitumor active metal complexes [17–22], our focus was on the synthesis of curcuminoids that could serve as building blocks to attach sugars like D-fructose or D-glucose [23]. Due to the easy accessibility to azido sugars [24–25] we decided to synthesise a range of curcuminoids bearing propargyl and pent-1-yn-5-yl ether groups as partners for “click” reactions [26]. We already observed that for the BF_2_ complex of bispropargyl functionalised bisdemethoxycurcumin, the BF_2_ group was hydrolysed under regular “click” reaction conditions [23].

In this paper we report on the synthesis and spectroscopic characterization of the above mentioned compound as well as seven other novel curcuminoid BF_2_ complexes containing terminal triple bonds in their side chains. We also optimized the reaction conditions for the cleavage of the BF_2_ group to release the curcuminoids as an alternative synthetic route to substituted curcumins.

## Results and Discussion

### Synthesis

#### BF_2_ complexes

First, we prepared the aldehydes **1a–h** as the starting materials by Williamson ether synthesis of the corresponding hydroxybenzaldehydes with either propargyl bromide in dry DMF or 5-chloropent-1-yne in dry acetonitrile. As bases we used potassium carbonate for the propargyl ethers and caesium carbonate for the ethers with the longer side chains. Aqueous work-up achieved the aldehydes in excellent yields of up to 98%. NMR spectroscopic results were found to be in good accordance with the data published elsewhere [27–30].

We received the BF_2_ complexes **2a–h** by aldol reactions between the in situ generated BF_2_ complex of 2,4-pentanedione and the corresponding aromatic aldehydes **1a–h**. Following a procedure reported originally by Zhang [14], we were able to isolate **2a–h** in yields ranging from 56 to 96% (Table 1).

**Table 1:** BF_3_·Et_2_O-promoted synthesis of curcuminoid–BF_2_ complexes **2a–h**.

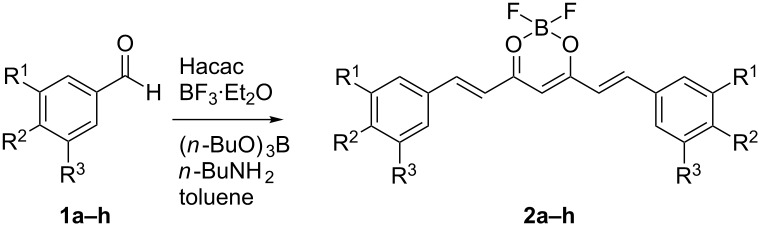				

Entry	R^1^	R^2^	R^3^	Yield (%) ^a^

**2a**	H	O-propargyl	OMe	90
**2b**	H	O-propargyl	H	96
**2c**	Br	O-propargyl	OMe	94
**2d**	Br	O-propargyl	H	65
**2e**	H	O-propargyl	O-propargyl	74
**2f**	H	OMe	O-propargyl	88
**2g**	H	OMe	O-pent-4-yn-1-yl	56
**2h**	H	O-pent-4-yn-1-yl	H	87

^a^Yield after recrystallization.

The amount of base required have to be increased from 0.1 to ca. 0.6 equiv due to the formation of HF, which forms *n*-butylammonium fluoride as a main impurity. Therefore, all complexes were purified by recrystallization from a mixture of acetone and water. Because most of the compounds were partially hydrolysed when heated to 80 °C in aqueous acetone solution, we carried out the purification at room temperature. The final compounds were characterized by ^1^H, ^11^B{^1^H}, ^13^C{^1^H} and ^19^F{^1^H} NMR spectroscopy, EI mass spectrometry, elemental analyses as well as UV–vis absorption and fluorescence spectroscopy.

The proton NMR spectra of all compounds exhibit the characteristic AB spin systems (*J* = approx. 16 Hz) occurring from the *trans*-olefinic protons in combination with a singlet at around 6.5 ppm. Signals in ^19^F{^1^H} NMR appeared as sharp singlets at around −140 ppm, while ^11^B{^1^H} NMR signals appear as up to 3 ppm broad singlets at about +0.9 ppm. No coupling between ^19^F and ^11^B nuclei was observed because of the high quadrupole moment of the ^11^B nucleus. Due to the high relative mass difference between both naturally occurring boron isotopes, signals for the ^10^B^19^F and ^11^B^19^F complexes with Δδ ≈ 0.1 ppm could be found in the ^19^F{^1^H} NMR spectrum. Several additional signals were observed after a few hours, as the compounds started to hydrolyse due to residual water in DMSO-*d*_6_. Resonances at −148.6 ppm in ^19^F{^1^H} and −1.3 ppm in ^11^B{^1^H} spectra (sharp singlet) could be assigned to BF_4_^−^ as the most common hydrolysis byproduct by comparison with HBF_4_ in DMSO-*d*_6_.

#### X-ray crystallography

**Figure 1:**
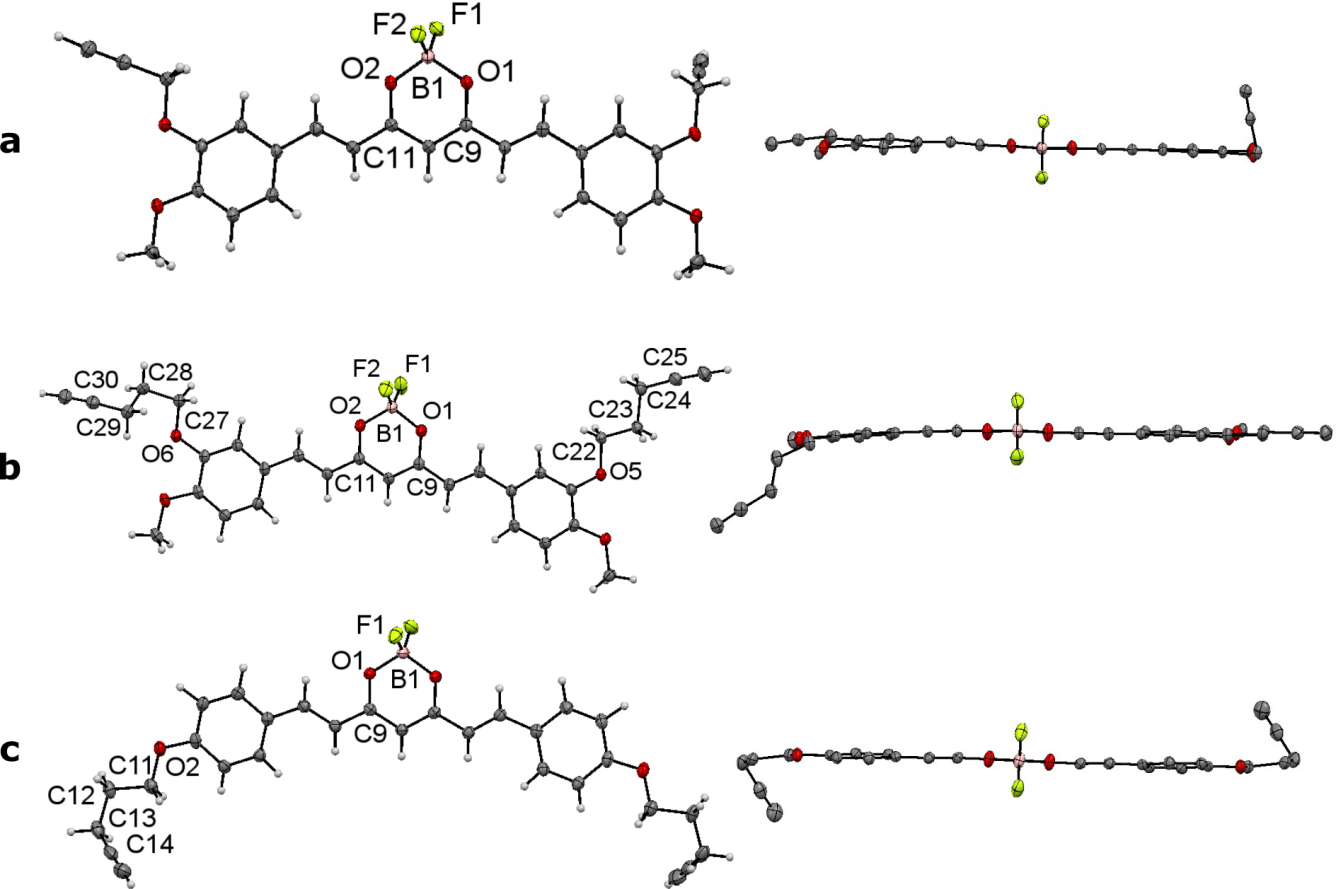
ORTEP drawings in side view (left) and top view (right) of complexes **2f** (a), **2g** (b) and **2h** (c). Hydrogen atoms are omitted from top view for clarity.

Compounds **2f**, **2g** and **2h** were also characterized by X-ray diffraction methods. Crystals suitable for analysis were grown by slow diffusion of *n*-hexane into CH_2_Cl_2_ solutions. All BF_2_ complexes show the expected tetrahedral coordination sphere around the boron atom and the all-*trans* geometry of the olefinic double bonds (Figure 1). Bond lengths and angles around the boron atom are in good accordance to values published for similar complexes [9]. One aromatic ring of **2f** is twisted by approx. 8° out of the plane formed by the ligand backbone and the second aromatic ring (Figure 1a). The twisting is stabilized by an intermolecular interaction between one propargyl CH_2_ proton and a methoxy oxygen atom (see Supporting Information File 1 for intermolecular distances as well as selected bond length and angles). This interaction also induces the respective propargyl group to be turned by approximately 70° out of the plane formed by the backbone. Due to sterical intermolecular interactions in **2g**, one pent-5-yne-1-yl chain is in *gauche/anti* conformation, while the other is in the more favoured *anti/anti* conformation (Figure 1b) with the torsion angles only slightly differing from the ideal 60° or 180°, respectively. The aromatic rings are almost coplanar with the backbone. In both cases, one of the longer side chains lays mostly within the molecule plane, while the other is turned out. The structure of complex **2h** shows *C*_2_ symmetry with both side chains in *gauche/anti* conformation. The deviation from the ideal angles is higher than that for **2g**. The plane formed by each phenyl ring is turned by approximately 5° out of the plane of the backbone.

#### BF_2_ cleavage

**Scheme 2:**
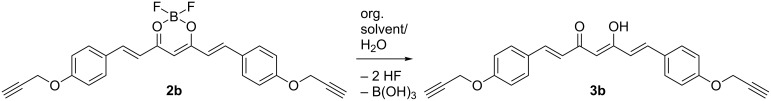
BF_2_ group hydrolysis of complex **2b**.

To hydrolyse the BF_2_ complexes and release the free ligands we investigated several mixtures of organic solvents and water in 4:1 ratios as well as dry THF as a control reaction at 65 °C (Table 2). Complex **2b** was chosen as the model compound (Scheme 2) as **3b** has been previously reported in the literature [31].

From the solvents screened, methanol and THF containing water show the best results (Table 2). Comparison with data reported in the literature [31] as well as the absence of a ^19^F{^1^H} NMR signal proved the success of the reaction. As expected, in the control reaction in dry THF no cleavage reaction was observed.

**Table 2:** Optimization of reaction conditions for BF_2_ group cleavage.

Solvent	Additive	Time (h)	Yield (%)

THF^a^	none	18	75^b^
MeOH^a^	none	18	80^b^
EtOH^a^	none	18	65^b^
DMF^a^	none	6	30^b^
dry THF	none	18	0
THF^a^	NaOH^c^	6	80^d^
MeOH ^a^	NaOH^c^	3.5	98^d^

^a^Containing 20% H_2_O; ^b^Yield after purification by column chromatography; ^c^5 wt % in water; ^d^Yield after recrystallization from acetone/water.

Interestingly, upon upscaling from 0.4 mmol to 4 mmol **2b** the yield of **3b** decreased to approx. 40%. Alterations of reaction time and temperature resulted in no significant changes.

**Scheme 3:**
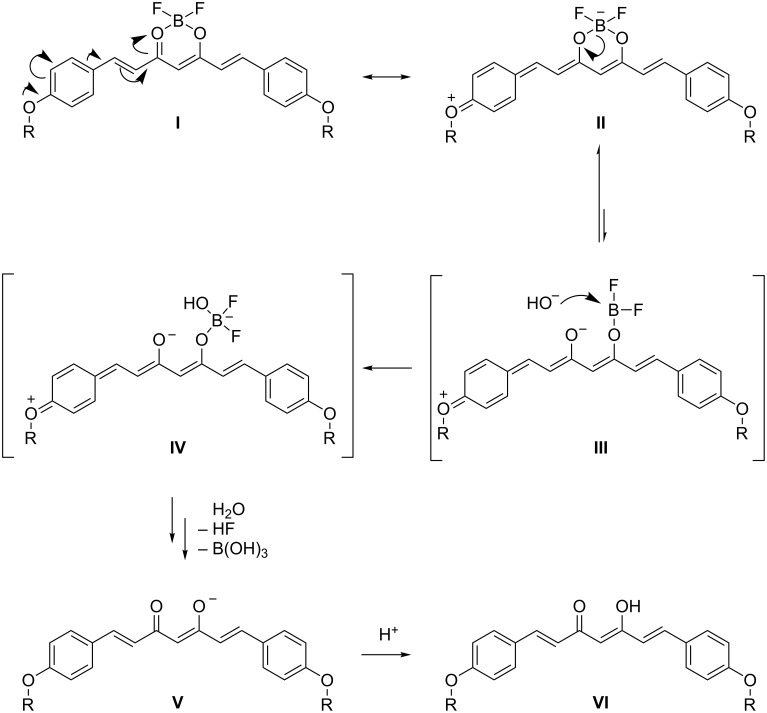
Suggested mechanism of BF_2_ complex hydrolysis.

Rao and co-worker report, that for the BF_2_ complex or unsubstituted curcumin a tautomeric form exists in solution, which acts as a weak acid [13]. Only the BF_2_ group of the deprotonated acid is able to become hydrolysed. In our case, although no free OH groups are present, we have also observed a similar pH value dependence as reported by Rao [13]. For this reason, we suggest that the possibility to form a quinoid structure is responsible for the increased stability of the BF_2_ complex in acidic solution (Scheme 3). At higher temperatures, there is an equilibrium between the quinoid form of the BF_2_ complex with a formal negative charge on the boron atom (**II**, “borate”) and a structure with one cleaved boron oxygen bond having the formal negative charge localized on the oxygen atom (**III**, best described as a difluoroboric acid ester). The ester is prone to a nucleophilic attack of a hydroxide ion, while the borate is not. After the nucleophilic attack a hydroxy difluoroborate (**IV**) is formed, which undergoes fast hydrolysis to boric acid, hydrogen fluoride and the corresponding curcuminoid in the anionic form (**V**). The latter finally becomes protonated by one equivalent of HF (**VI**). If no additional base is present, the hydroxide concentration decreases with ongoing hydrolysis so far, that the reaction effectively stops.

To confirm our suggestion we carried out the hydrolysis reactions of 4 mmol **2b** in 80% aq methanol or THF again with the addition of 10 mol % of NaOH. The final yields could be increased to 98 and 80%, respectively. Additionally, we could observe a much shorter reaction time. This proves the necessity for a base to be present to complete the reaction.

We were able to apply this procedure to BF_2_ complexes **2a–c** and **2e** to receive the curcuminoids **3** in good to excellent yields. **2d** and **2f–h** were found to possess a relatively low solubility in methanol and especially **2d** to be more sensitive to nucleophilic bases when heated in solution. To increase the solubility, most of the water added was replaced by DMSO (Table 3). This improved the solubility and did not induce any additional impurities. We also changed the base from NaOH to triethylamine for these compounds to avoid partial decomposition. The disadvantage of triethylamine was a longer reaction time of seven to eighteen hours, probably due to the lower hydroxide concentration.

**Table 3:** Hydrolysis reactions.

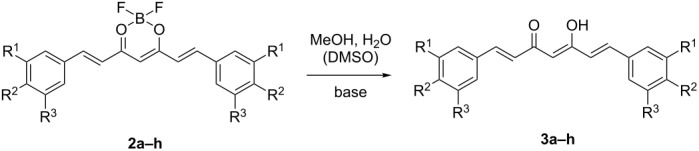						

Entry	R^1^	R^2^	R^3^	Solvent (MeOH/DMSO/H_2_O)	Base	Yield (%)^a^

**3a**	H	O-propargyl	OMe	8:0:2	NaOH^b^	87
**3b**	H	O-propargyl	H	8:0:2	NaOH	92
**3c**	Br	O-propargyl	OMe	8:0:2	NaOH	92
**3d**	Br	O-propargyl	H	8:1.5:0.5	TEA	n/a
**3e**	H	O-propargyl	O-propargyl	8:0:2	NaOH	84
**3f**	H	OMe	O-propargyl	8:1.5:0.5	TEA	90
**3g**	H	OMe	O-pent-4-yn-1-yl	8:1.5:0.5	TEA	80
**3h**	H	O-pent-4-yn-1-yl	H	8:1.5:0.5	TEA	95

^a^Yield after recrystallization; ^b^5 wt % solution in water.

Regarding the ^1^H NMR spectra, we found that all crude products contain small amounts of decomposition products resulting from base induced cleavage of the backbone. Recrystallization from acetone or ethanol and water mixtures gave the pure products **3a–c** and **3e–h** as yellow or orange solids in good to excellent yields. They were characterized by ^1^H and ^13^C{^1^H} NMR spectroscopy, mass spectrometry, UV-visible spectroscopy and elemental analysis. For **3d** we found two sets of NMR signals in both ^1^H and ^13^C{^1^H} NMR spectra with relative intensities of 1:0.25, which could be assigned to be no starting material. These did not change upon alteration of NMR solvent or temperature. Also, no [M]^+^ signals or any expected fragments for **3d** were found in EI or ESIMS spectra.

#### Optical spectroscopy

**Figure 2:**
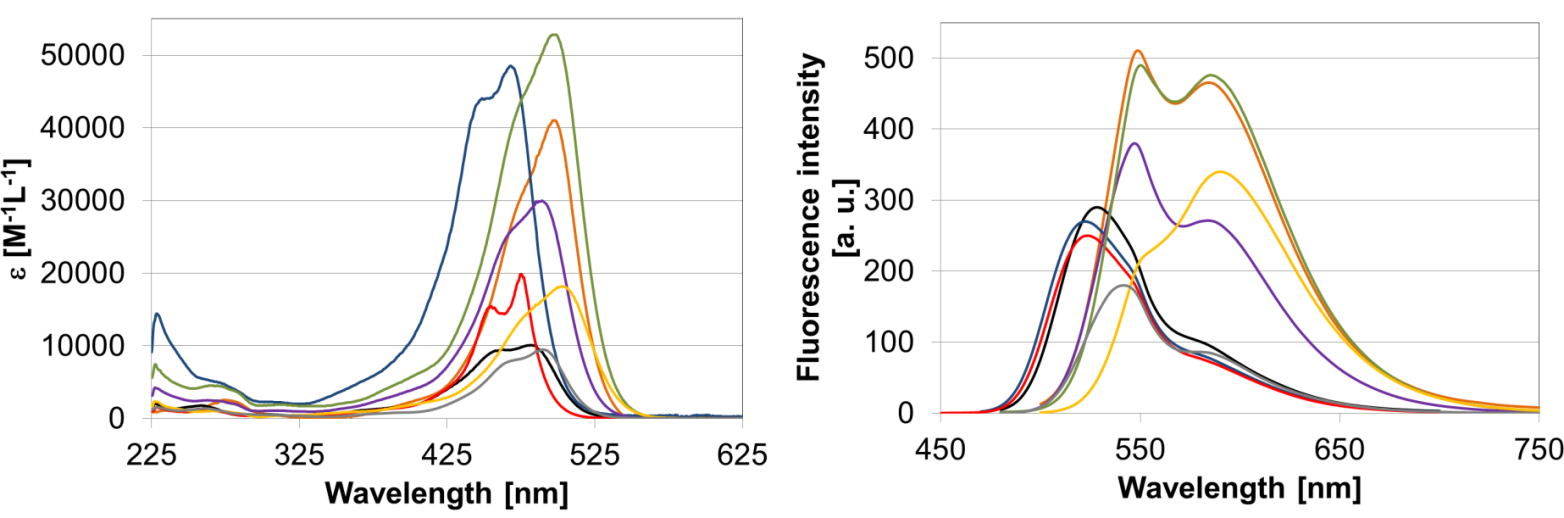
Absorbance (left) and emission (right) spectra of compounds **2a** (orange), **2b** (black), **2c** (blue), **2d** (red), **2e** (purple), **2f** (green), **2g** (yellow) and **2h** (grey) in CH_2_Cl_2_.

To investigate their properties as fluorescent dyes we measured the UV–vis absorption and fluorescence spectra of the BF_2_ complexes **2a–h**. Measurements were carried out in dichloromethane at room temperature and under ambient atmosphere. The results are shown in Figure 2.

All BF_2_ complexes show strong absorption bands with absorption maxima between 475 and 503 nm resulting from π–π^*^ transitions [9]. Extinction coefficients range from roughly 9500 to over 50000 M^−1^ cm^−1^ (Table 4). All absorption curves show secondary maxima or shoulders at slightly shorter wavelengths.

**Table 4:** Absorption and emission spectral properties of BF_2_ complexes **2a–h** in CH_2_Cl_2_. See Supporting Information File 1 for details on the measurement setup.

Compound	λ_max_^abs^ (nm)	ε · 10^-3^ (M^−1^ cm^−1^)	λ_max_^em^ (nm)	Φ^a^	τ^b^ (ns)	Stokes-shift (cm^−1^)

**2a**	497	41.0	548	0.51	1.68	1873
**2b**	480	10.1	528	0.29	1.21	1894
**2c**	476	48.5	522	0.27	1.01	1851
**2d**	475	19.8	524	0.25	1.01	1969
**2e**	487	30.0	547	0.38	1.56	2252
**2f**	497	52.8	550	0.49	1.55	1939
**2g**	503	18.2	590	0.34	1.57	2932
**2h**	489	9.5	542	0.18	1.50	2000

^a^Fluorescence quantum yield was determined against rhodamine 6G (Φ = 0.95) in ethanol. ^b^Fluorescence lifetime upon 400 nm excitation.

**Figure 3:**
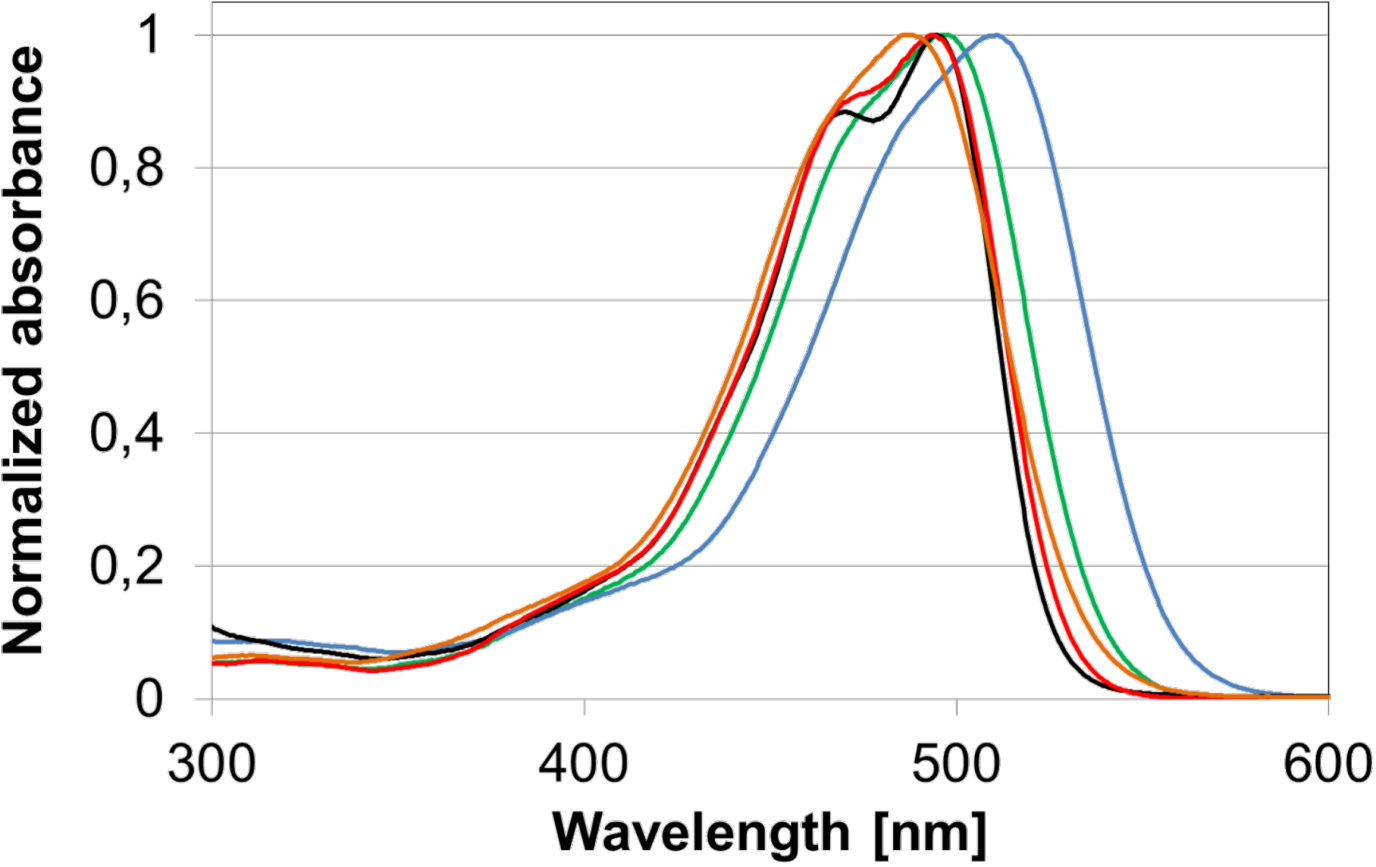
Absorbance spectra of **2b** in methanol (orange), tetrahydrofuran (red), toluene (black), dichloromethane (green) and dimethyl sulfoxide (blue).

As solvatochromism is a known property for curcumin and its derivatives [32–33], we investigated the solvatochromism of **2b** as an example compound in five different solvents (Figure 3). Solvents were chosen by their *E*_T_(30) values of polarity as determined by Reichardt [34]. With rising solvent polarity, the vibrational structure of the absorption band is being lost. In toluene, THF and dichloromethane the compound shows only weak solvatochromism. Interestingly, a positive solvatochromism relative to the more nonpolar solvents is appearing in DMSO, while the absorption band is slightly being shifted hypsochromically in methanol.

**Figure 4:**
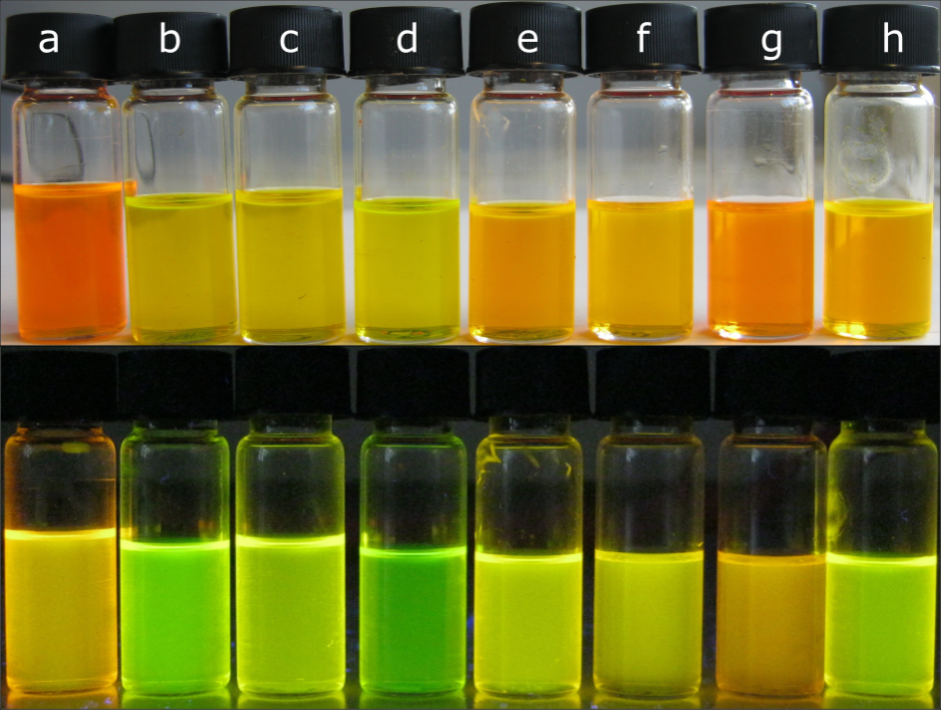
Compounds **2a–h** in dichloromethane solution in daylight (top) and under 365 nm irradiation (bottom).

In solution, upon excitation at 365 nm, green, yellow or orange fluorescence can be observed (Figure 4).

All fluorescence spectra are characterized by two maxima, one in the range of 520–550 nm and one in the range of 580–590 nm. However, there are distinct differences in the intensity ratios of these two maxima between the compounds. For **2b–d**, the lower energy maximum appears as a shoulder, for **2a** and **2e–h**, it appears as a local maximum, and for **2g** it represents the global maximum of emission. This trend can be rationalized by taking the electronic structure of the compounds into account. With increasing electron density of the aromatic system, the emission intensity in the low-energy regime of the spectrum increases. It is also noteworthy, that regarding **2a** and **2f**, which are regioisomers, the second fluorescence band is more intense for **2f** than for **2a**. For the complexes containing a brominated phenyl ring, the presence of an additional electron-donating methoxy group has almost no impact on the fluorescence properties.

## Conclusion

We have synthesized a series of novel curcuminoid–BF_2_ complexes by an improved synthetic route. All complexes were received in high yields and purities and characterized by ^1^H, ^11^B, ^13^C and ^19^F NMR spectroscopy, mass spectrometry and elemental analysis. We found the complexes to possess high absorption in the range of 475 to 500 nm and strong fluorescence between 520 and 590 nm, resulting in Stokes shifts of up to 3000 cm^−1^. Finally, an effective strategy to hydrolyse the BF_2_ group and release the curcuminoids could be established using aqueous methanol and mild basic conditions. In some cases, when the solubility of the substrates was low, DMSO was used as an additional solvent. These compounds can act as building blocks for the attachment of biomolecules via “click” chemistry.

## Supporting Information

File 1 Experimental data, X-ray crystallographic details, selected bond lengths and angles, copies of NMR spectra.

File 2 CIF files for complexes **2f**, **2g** and **2h**. These data (CCDC-1526555 for **2f**, CCDC-1526556 for **2g**, and CCDC-1526557 for **2h**) can be obtained free of charge from The Cambridge Crystallographic Data Centre via http://www.ccdc.cam.ac.uk/data_request/cif.
