# Supplementary material for: Curcuminoid–BF2 complexes: Synthesis, fluorescence and optimization of BF2 group cleavage
Source: Beilstein J Org Chem. 2017 Oct 26;13:2264–72. doi: 10.3762/bjoc.13.223 (PMC5669223; doi:10.3762/bjoc.13.223)
Supplement: File 1 — Experimental data, X-ray crystallographic details, selected bond lengths and angles, copies of NMR spectra. [file Beilstein_J_Org_Chem-13-2264-s001.pdf]

Supporting Information

for

Curcuminoid–BF_2 complexes: Synthesis, fluorescence and optimization of BF_2 group cleavage

Henning Weiss[1], Jeannine Reichel[1], Helmar Görls[1], Kilian Rolf Anton Schneider[2], Mathias Micheel[3], Michael Pröhl[4], Michael Gottschaldt[4], Benjamin Dietzek[2,3] and Wolfgang Weigand[1,4]*

Address: [1]Institute for Inorganic and Analytical Chemistry, Friedrich-Schiller-Universität Jena, Humboldtstrasse 8, 07743 Jena, Germany, [2]Institute for Physical Chemistry, Friedrich-Schiller-Universität Jena, Helmholtzweg 4, 07743 Jena, Germany, [3]Leibniz Insitute of Photonic Technology (IPHT), Albert-Einstein-Straße 9, 07745 Jena, Germany and [4]Jena Center of Soft Matter, Friedrich-Schiller-Universität Jena, Philosophenweg 7, 07743 Jena, Germany

Email: Wolfgang Weigand - wolfgang.weigand@uni-jena.de

*Corresponding author

Experimental data, X-ray crystallographic details, selected bond lengths and angles, copies of NMR spectra

Table of contents

1. General remarks S3

2. Synthesis of aldehydes **1** and characterization data S4

3. Synthesis of BF_2 complexes **2** and characterization data S6

4. Synthesis of curcuminoids **3** and characterization data S11

5. X-ray crystallographic data for **2f**, **2g** and **2h** S16

6. References S18

7. Intermolecular interactions in **2f** S19

8. Copies of NMR spectra for **2** and **3** S20

1: General remarks:

All, except hydrolysis reactions, were carried out under nitrogen atmosphere using standard Schlenk techniques. DMF and acetonitrile were dried over calcium hydride, toluene over sodium and benzophenone, while THF was stored over KOH. All solvents were distilled prior to use. Starting materials were commercially sourced and used without further purification. 3-Bromo-4-hydroxy-5-methoxybenzaldehyde and 3-bromo-4-hydroxybenzaldehyde were received following a literature procedure [1]. NMR spectra were recorded on Bruker Avance 200, 400 or 600 spectrometers in regular borosilicate glass tubes. Chemical shifts are reported in ppm relative to external TMS for ^{1}H and $^{13}C\{^{1}H\}$, $FCCl_3$ for $^{19}F\{^{1}H\}$ and $BF_3{\cdot}OEt_2$ for $^{11}B\{^{1}H\}$ NMR, respectively. Coupling constants are reported in Hz. EI and ESI–TOF–MS were recorded on SSQ 710 and MAT95XL (Finnigen MAT) devices in positive mode. UV–vis spectra were measured on a Specord D 600 spectrometer by Analytik Jena. Fluorescence spectra were recorded on a Jasco FP-6300 fluorescence spectrometer. Relative fluorescence quantum yields were measured by comparing the areas under the fluorescence emission spectrum of dilute ($0.02 < A < 0.06$) solutions of **2a–h** in dichloromethane. Rhodamine 6G in ethanol ($\phi = 0.95$) was used as the standard. Final values were determined by using equations reported in the literature [2]. Spectrally resolved emission decay curves were determined employing a Hamamatsu HPDTA streak camera. Samples were excited by pulses centred at 400 nm created by frequency doubling the output of a Ti:sapphire laser (Tsunami, Newport Spectra-Physics GmbH). The repetition rate of the fundamental is reduced to 400 kHz by a pulse selector (model 3980, Newport Spectra-Physics GmbH). Emission was collected for solutions from a 1 cm cuvette in a 90° angle and spectrally dispersed on the detector using a CHROMEX spectrograph.

Measurements were performed with a polarizer set to magic angle, i.e., set to 54.7° with respect to the excitation polarization, in the detection path. Spectrally and time-resolved emission data was spectrally integrated and analysed using DecayFit software [1]. All decay curves could adequately fitted by a monoexponential decay model.

2. Synthesis of aldehydes 1 and characterization data:

Aldehydes **1** were synthesized following two general procedures. Analytical results are in good accordance to data published elsewhere [3–7].

General procedure (**1a–f**): The respective hydroxybenzaldehyde (6 mmol) was dissolved in DMF and K_2CO_3 (3.3 g, 24 mmol) and propargyl bromide (0.63 mL, 6.6 mmol; 80 wt % in toluene) were added. The resulting suspension was stirred at room temperature overnight and poured into ice water. The precipitated solid was collected by filtration, washed with water and dried in air to receive the respective ethers as off-white solids.

General procedure (**1g–h**): The respective hydroxybenzaldehyde (6 mmol) was dissolved in acetonitrile (30 mL) and Cs_2CO_3 (2.9 g, 9 mmol), KI (1 g, 6 mmol) and 5-chloropent-1-yne (0.7 mL, 6.6 mmol) were added. The mixture was stirred under reflux overnight and cooled to room temperature. Ethyl acetate and water (15 mL each) were added and the organic phase was extracted with water (3 × 15 mL) and brine, dried (Na_2SO_4) and evaporated under reduced pressure to yield the respective ethers as pale yellow solids.

3-Methoxy-4-propargyloxybenzaldehyde (1a) from 3-methoxy-4-hydroxy benzaldehyde: Yield 95%. ^{1}H-NMR (400 MHz, $CDCl_3$): δ 9.85 (s, 1H); 7.45 (dd, 1H, *J*

= 8.2, 1.8 Hz); 7.41 (d, 1H *J* = 1.8 Hz); 7.13 (d, 1H, *J* = 8.2 Hz); 4.84 (d, 2H, *J* = 2.4 Hz); 3.92 (s, 3H); 2.56 (t, 1H, *J* = 2.4 Hz).

4-Propargyloxybenzaldehyde (1b) from 4-hydroxybenzaledhyde: Yield 99%. ^{1}H-NMR (400 MHz, $CDCl_3$): δ 9.88 (s, 1H); 7.84 (d, 2H, *J* = 8.8 Hz); 7.07 (d, 2H, *J* = 8.7 Hz); 4.76 (d, 2H, *J* = 2.3 Hz); 2.57 (t, 1H, *J* = 2.4 Hz). ^{13}C{^{1}H}-NMR (101 MHz, $CDCl_3$): δ 190.9; 162.5; 132.0; 130.7; 115.3; 77.6; 76.5; 56.0.

3-Bromo-4-propargyloxy-5-methoxybenzaldehyde (1c) from 3-bromo-4-hydroxy-5-methoxybenzaldehyde: Yield 94%. ^{1}H-NMR (600 MHz, $CDCl_3$): δ 9.84 (s, 1H); 7.65 (d, 1H *J* = 1.8 Hz); 7.38 (d, 1H, *J* = 1.7 Hz); 4.87 (d, 2H *J* = 2.4 Hz); 3.93 (s, 3H); 2.49 (t, 1H, *J* = 2.4 Hz). ^{13}C{^{1}H}-NMR (151 MHz, $CDCl_3$): δ 190.0; 154.3; 149.3; 133.6; 128.8; 118.8; 110.1; 78.2; 76.3; 60.3; 56.4.

3-Bromo-4-propargyloxybenzaldehyde (1d) from 3-bromo-4-hydroxybenzaldehyde: Yield 92%. ^{1}H-NMR (400 MHz, $CDCl_3$) δ 9.85 (s, 1H); 8.09 (d, 1H, *J* = 2.0 Hz, 1H); 7.82 (dd, 1H, *J* = 8.5, 2.0 Hz); 7.17 (d, 1H, *J* = 8.5 Hz); 4.87 (d, 2H, *J* = 2.4 Hz); 2.60 (t, 1H, *J* = 2.4 Hz). ^{13}C{^{1}H}-NMR (101 MHz, $CDCl_3$): δ 189.7; 158.7; 134.9; 131.4; 130.9; 113.3; 113.2; 77.3; 77.0; 57.0.

3,4-Bis-(propargyloxy)benzaldehyde (1e) from 3,4-dihydroxybenzaldehyde and 2.4 eq. of propargyl bromide: Yield 83%. The product was purified by column chromatography (silica gel, ethyl acetate / hexanes 1:2). ^{1}H-NMR (400 MHz, $CDCl_3$): δ 9.88 (s, 1H); 7.58 (d, 1H, *J* = 1.8 Hz); 7.53 (dd, 1H, *J* = 8.2, 1.8 Hz); 7.18 (d, 1H, *J* = 8.3 Hz); 4.86 (d, 2H, *J* = 2.4 Hz); 4.83 (d, 2H, *J* = 2.4 Hz); 2.57 (t, 1H, *J* = 2.4 Hz); 2.55 (t, 1H, *J* = 2.4 Hz).

3-Propargyloxy-4-methyoxybenzaldehyde (1f) from isovanillin: Yield 95%. ^{1}H-NMR (600 MHz, $CDCl_3$): δ 9.78 (s, 1H); 7.46 (d, 1H *J* = 1.6 Hz); 7.44 (dd, 1H, *J* = 8.2,

1.7 Hz); 6.94 (d, 1H, *J* = 8.2 Hz); 4.75 (d, 2H, *J* = 2.4 Hz, 2H); 3.88 (s, 3H); 2.52 (t, 1H, *J* = 2.4 Hz). $^{13}C\{^1H\}$-NMR (151 MHz, $CDCl_3$): δ 190.6; 154.9; 147.2; 129.8; 127.2; 112.0; 110.9; 77.8; 76.5; 56.6; 56.1.

3-(Ppent-4-yn-1-yloxy)-4-methoxybenzaldehyde (1g) from isovanillin: Yield 94%. ^1H-NMR (400 MHz, $CDCl_3$): δ 9.84 (s, 1H); 7.45 (dd, 1H, *J* = 8.2, 1.8 Hz); 7.42 (d, 1H, *J* = 1.8 Hz); 6.97 (d, 1H, *J* = 8.1 Hz); 4.18 (t, 2H, *J* = 6.3 Hz); 3.94 (s, 3H); 2.43 (td, 2H, *J* = 6.9, 2.7 Hz); 2.12 – 2.03 (m, 2H); 1.98 (t, 1H, *J* = 2.6 Hz).

4-(Pent-4-yn-1-yloxy)-benzaldehyde (1h) from 4-hydroxybenzaledhyde: Yield 93%. ^1H-NMR (400 MHz, DMSO-d_6): δ 9.86 (s, 1H); 7.85 (d, 1H, *J* = 8.7 Hz); 7.11 (d, 1H, *J* = 8.7 Hz); 4.14 (t, 2H, *J* = 6.2 Hz); 2.80 (t, 1H *J* = 2.8 Hz); 2.34 (td, 2H, *J* = 7.1, 2.8 Hz); 1.97 – 1.88 (m, 2H). $^{13}C\{^1H\}$-NMR (101 MHz, DMSO-d_6): δ 191.3; 163.5; 131.9; 129.7; 114.9; 83.5; 71.7; 66.6; 27.5; 14.5.

3. Synthesis of BF_2 complexes 2 and characterization data:

General procedure (modified from lit. [8]):

2,4-Pentanedione (0.3 mL, 3 mmol) and BF_3·OEt_2 (0.55 mL, 4.4 mmol) were dissolved in toluene (5 mL) and stirred at 65 °C for 2 h. Then, the respective aldehyde (6 mmol) dissolved in the minimum amount of toluene was transferred into the solution, followed by tri-*n*-butyl borate (1.85 mL, 7.4 mmol). Stirring was continued for 30 min and *n*-butylamine (approx. 0.6 equiv) was added dropwise until a color change was observed and a solid began to precipitate. The resulting suspension was kept stirring at 65 °C overnight. After cooling to room temperature the crude product was separated by filtration and washed with small amounts of cold

toluene and water. The isolated solid was dissolved in acetone at room temperature and water was added slowly. The resulting suspension was filtered and the isolated solid washed with water and dried in air to receive the pure product.

2a · H_2O:

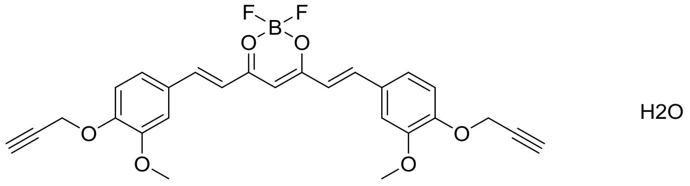

Yield 90%, purple solid. m. p.: 221.5 – 222.5 °C. ^{1}H-NMR (400 MHz, DMSO-d_6): δ 7.97 (d,2H, *J* = 15.6 Hz), 7.56 (d, 2H, *J* = 1.5 Hz), 7.52 (d, 2H, *J* = 8.4 Hz, 2,1 Hz), 7.13 (d, 2H, *J* = 8.5 Hz), 7.07 (d, 2H, *J* = 15.7 Hz), 6.55 (s, 1H), 4.88 (d, 4H, *J* = 2.2 Hz), 3.86 (s, 6H), 3.60 (t, 2H, *J* = 2.2 Hz). ^{13}C{^{1}H}-NMR (101 MHz, DMSO-d_6): δ 179.11, 152.88, 146.71, 146.65, 126.85, 125.82, 119.02, 113.53, 112.26, 101.26, 78.93, 78.56, 56.09, 55.85. ^{11}B{^{1}H}-NMR (128 MHz, DMSO-d_6): δ 0.9 (s). ^{19}F{^{1}H}-NMR (188 MHz, DMSO-d_6): δ -138.4 (s). EI-MS: m/z (%) = 492 (15); 387 (25); 215 (100). UV/Vis λ_{max} [nm] (ε [$M^{-1}cm^{-1}$]): 497 (41013). Anal. calcd. for $C_{27}H_{23}BF_2O_6 \cdot H_2O$: C (63.55); H (4.94). Found: C (63.27); H (4.72).

2b:

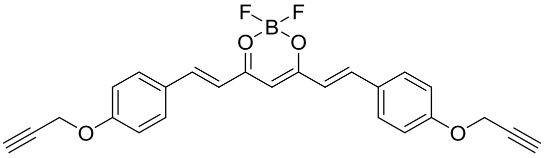

Yield 96%, red solid. m. p.: 253 – 254 °C. ^{1}H-NMR (600 MHz, DMSO-d_6): δ 7.98 (d, 2H, *J* = 15.4 Hz, H-5); 7.85 (d, 4H, *J* = 8.5 Hz); 7.10 (d, 4H, *J* = 8.3 Hz); 7.07 (d, 2H, *J* = 15.4 Hz); 6.53 (s, 1H); 4.91 (s, 4H); 3.62 (s, 2H). ^{13}C{^{1}H}-NMR (150 MHz, DMSO-d6): δ 55.8; 78.8; 101.8; 115.6; 119.2; 127.5; 131.6; 146.3; 160.4; 179.4. ^{19}F{^{1}H}-

NMR (188 MHz, DMSO-d_6): δ -138.1. $^{11}B\{^1H\}$-NMR (128 MHz, DMSO-d_6): δ -1.26. EI-MS: m/z (%) = 432 (60); 413 (20); 393 (5); 327 (35); 185 (100). UV/Vis λ_{max} [nm] (ε [$M^{-1}cm^{-1}$]): 480 (10128). Anal. calcd. for $C_{25}H_{19}BF_2O_4$: C (69.47); H (4.43). Found: C (69.44); H (4.56).

2c · H_2O:

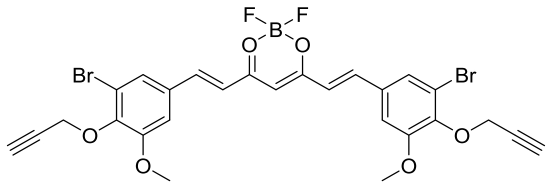

Yield 94%, orange solid. m. p.: 279.5 – 280 °C. ^1H-NMR (600 MHz, DMSO-d_6): δ 7.99 (d, 2H, *J* = 15.7 Hz); 7.79 (d, 2H, *J* = 2.0 Hz); 7.61 (d, 2H, *J* = 2.2 Hz); 7.32 (d, 2H, *J* = 15.8 Hz); 6.53 (s, 1H); 4.83 (d, 4H, *J* = 2.6 Hz); 3.91 (s, 6H); 3.57 (t, 2H, *J* = 2.5 Hz). $^{13}C\{^1H\}$-NMR (150 MHz, DMSO-d_6): δ 179.9; 153.5; 146.0; 145.2; 131.9; 125.8; 122.2; 117.9; 113.4; 78.9; 78.7; 59.8; 56.5. $^{19}F\{^1H\}$-NMR (188 MHz, DMSO-d_6): δ -137.6 (s). $^{11}B\{^1H\}$-NMR (193 MHz, DMSO-d_6): δ 0.90 (s). EI-MS: m/z (%) = 650 (1); 611 (5); 545 (2); 293 (2); 96 (100). UV/Vis λ_{max} [nm] (ε [$M^{-1}cm^{-1}$]): 476 (48514). Anal. calcd. for $C_{27}H_{21}BBr_2F_2O_6{\cdot}H_2O$: C (48.54); H (3.47). Found: C (48.49); H (3.29).

2d:

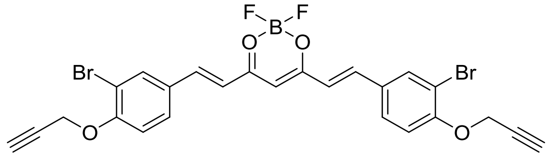

Yield 92%, orange solid. m. p.: 251 °C (dec.). ^1H-NMR (600 MHz, DMSO-d_6): δ 8.22 (s, 2H); 7.96 (d, 2H, *J* = 15.7 Hz); 7.90 (d, 2H, = 8.3 Hz); 7.29 (d, 2H, *J* = 8.5 Hz); 7.22 (d, 2H, *J* = 15.7 Hz); 6.50 (s, 1H); 5.03 (s, 4H); 3.68 (s, 2H). $^{13}C\{^1H\}$-NMR (150

MHz, DMSO-d$_6$): δ 179.6; 156.2; 145.0; 133.8; 131.2; 129.0; 120.6; 114.3; 112.0; 102.4; 79.3; 78.2; 56.9. ^{19}F{^{1}H}-NMR (188 MHz, DMSO-d$_6$): δ = -137.8 (s). ^{11}B{^{1}H}-NMR (128 MHz, DMSO-d$_6$): δ = 0.88 (s). EI-MS: m/z (%) = 590 (2); 485 (2); 39 (100). UV/Vis λ_{max} [nm] (ε [M^{-1}cm^{-1}]): 475 (19847). Anal. calcd. for $C_{25}H_{17}BBr_2F_2O_4$: C (50.89); H (2.90). Found: C (50.58); H (2.83).

2e:

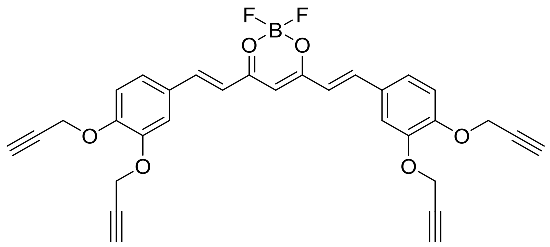

Yield 74%, purple solid. m. p.: 251.5 – 252.5 °C. ^{1}H-NMR (400 MHz, DMSO-d$_6$): δ = 7.98 (d, 2H, *J* = 15.6 Hz); 7.59 (d, 2H, *J* = 1.8 Hz); 7.52 (dd, 2H, *J* = 8.6 Hz, *J* = 1.8 Hz); 7.18 (d, 2H, *J* = 8.6 Hz); 7.10 (d, 2H, *J* = 15.7 Hz); 6.58 (s, 1H); 4.93 (d, 4H, *J* = 2.3 Hz); 4.90 (d, 4H, *J* = 2.3 Hz); 3.64 (t, 2H, *J* = 2.3 Hz); 3.61 (t, 2H, *J* = 2.3 Hz). ^{13}C{^{1}H}-NMR (100 MHz, DMSO-d$_6$): δ = 179.3; 150.5; 147.0; 146.6; 127.6; 125.2; 119.5; 113.9; 102.4; 78.92; 78.87; 78.7; 56.1. ^{19}F{^{1}H}-NMR (188 MHz, DMSO-d$_6$): δ = -138.27. ^{11}B{^{1}H}-NMR (193 MHz, DMSO-d$_6$): δ = -0.88. EI-MS: m/z (%) = 540 (12); 344 (48); 305 (84); 91 (55); 28 (100). UV/Vis λ_{max} [nm] (ε [M^{-1}cm^{-1}]): 487 (29973). Anal. calcd. for $C_{31}H_{23}BF_2O_6$: C (68.91); H (4.29). Found: C (68.74); H (4.37).

2f · 0.4 H_2O:

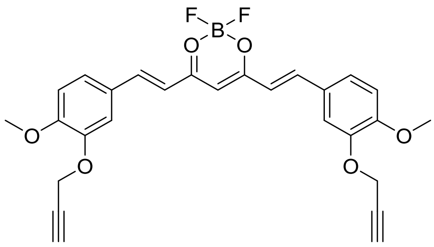

Yield 88%, red solid. m. p.: 208.5 – 209.5 °C. ^{1}H-NMR (400 MHz, DMSO-d_6): δ = 7.96 (d, 2H, *J* = 15.6 Hz); 7.55 (s, 2H); 7.50 (d, 2H, *J* = 8.3 Hz); 7.11 (d, 2H, *J* = 8.4 Hz); 7.05 (d, 2H, *J* = 15.7 Hz); 6.54 (s, 1H); 4.88 (d, 4H, *J* = 2.6 Hz); 3.85 (s, 6H). ^{13}C{^{1}H}-NMR (101 MHz, DMSO-d_6): δ 179.2, 152.9, 146.8, 146.7, 129.0, 128.3, 126.9, 125.9, 125.4, 119.1, 113.6, 112.3, 79.0, 78.6, 56.2, 55.9, 38.7. ^{19}F{^{1}H}-NMR (188 MHz, DMSO-d_6): δ = -138.3. ^{11}B{^{1}H}-NMR (193 MHz, DMSO-d_6): δ = 0.90. EI-MS: m/z (%) = 492 (60); 426 (42); 387 (44); 215 (100). UV/Vis λ_{max} [nm] (ε [$M^{-1}cm^{-1}$]): 497 (52812). Anal. calcd. for $C_{27}H_{23}BF_2O_6$.0.4H_2O: C (64.93); H (4.80). Found: C (64.96); H (4.70).

2g · 0.25 H_2O:

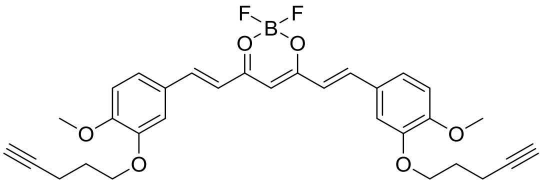

Yield 56%, dark red solid. m. p.: 205 – 206 °C. ^{1}H-NMR (400 MHz, DMSO-d_6): δ = 7.95 (d, 2H, *J* = 15.7 Hz); 7.49 (d, 2H, *J* = 2.0 Hz); 7.45 (dd, 2H, *J* = 8.5, 2.0 Hz); 7.08 (d, 2H, *J* = 15.7 Hz); 7.07 (d, 2H, *J* = 15.7 Hz); 6.49 (s, 1H); 4.11 (t, 4H, *J* = 6.0 Hz); 3.84 (s, 6H); 2.85 (t, 2H, *J* = 2.4 Hz); 2.34 (td, 2H, *J* = 7.1, 2.8 Hz); 1.90 – 1.94 (m, 4H). ^{13}C{^{1}H}-NMR (100 MHz, DMSO-d_6): δ = 179.1; 152.7; 148.3; 146.7; 127.1; 125.2; 118.9; 112.5; 112.0; 101.3; 83.7; 71.6; 66.8; 55.8; 27.7; 14.5. ^{19}F{^{1}H}-NMR (188 MHz, DMSO-d6): δ = -138.3. ^{11}B{^{1}H}-NMR (193 MHz, DMSO-d_6): δ = 0.90. EI-MS: m/z (%) = 548 (18); 529 (23); 482 (98); 243 (60); 177 (100). UV/Vis λ_{max} [nm] (ε [$M^{-1}cm^{-1}$]): 503 (18194). Anal. calcd. for $C_{31}H_{31}BF_2O_6$·0.25H_2O: C (67.34); H (5.74). Found: C (67.28); H (5.43).

2h · 0.5 H_2O:

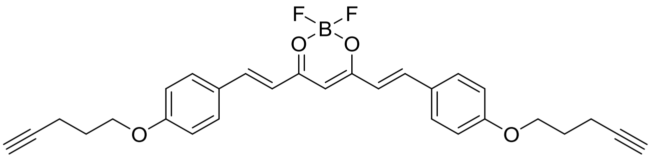

Yield 87%, orange solid. m. p.: 225 – 226 °C. ^{1}H-NMR (600 MHz, DMSO-d$_6$): δ 7.97 (d, 2H, *J* = 15.6 Hz); 7.83 (d, 4H, *J* = 8.6 Hz); 7.06 (d, 4H, *J* = 8.4 Hz); 7.06 (d, 2H, *J* = 16.0 Hz); 6.51 (s, 1H); 4.13 (t, 4H, *J* = 6.2 Hz); 2.82 (t, 2H *J* = 3.1 Hz); 2.34 (td, 4H, *J* = 7.0, 2.9 Hz); 1.91 (p, 4H, *J* = 6.8 Hz). ^{13}C{^{1}H}-NMR (150 MHz, DMSO-d$_6$): δ 179.2; 161.8; 146.4; 131.8; 126.9; 118.8; 115.3; 101.6; 83.5; 71.7; 66.4; 27.6; 14.4. ^{19}F{^{1}H}-NMR (188 MHz, DMSO-d$_6$): δ = -138.2. ^{11}B{^{1}H}-NMR (193 MHz, DMSO-d$_6$): δ = 0.88. EI-MS: m/z (%) = 488 (63); 469 (16); 422 (36); 344 (88); 213 (100); 147 (58). UV/Vis λ_{max} [nm] (ε [$M^{-1}cm^{-1}$]): 489 (9481). Anal. calcd. for $C_{29}H_{27}BF_2O_4 \cdot 0.5\ H_2O$: C (70.04); H (5.67). Found: C (70.20); H (5.75).

4. Synthesis of curcuminoids 3 and characterization data:

General procedure (3a–c and 3e): The respective curcuminoid–BF_2 complex **2** (1.5 mmol) was dissolved in methanol (40 mL) and water (10 mL). NaOH (3 mmol, 5% w/w aqueous solution) was added upon which the color turned to reddish green. The solution was stirred at 70 °C for 3 to 4 h and monitored by TLC (ethyl acetate/*n*-hexane 2:3). After the reaction was completed, the solution was cooled to room temperature and acidified with 2 M HCl. Methanol was removed under reduced pressure and the precipitated solid was collected by filtration, washed neutral with water, recrystallized from acetone/water or ethanol/water and dried in air.

General procedure (3d and 3f–h): The respective curcuminoid–BF_2 complex **2** (0.25 mmol) was dissolved in DMSO (10 mL) and methanol (200 mL) and water (5 mL) were added subsequently. To the resulting suspension was added triethylamine (0.5 mmol) upon which the color turned to orange-green. The solution was stirred at 50 °C for 7 to 18 h and monitored by TLC (ethyl acetate/*n*-hexane 1:1). After the reaction was completed, the solution was cooled to room temperature and acidified with 2 M HCl. Methanol was removed under reduced pressure and the resulting oil was poured into ice water. The precipitated solid was isolated by filtration, washed five times with water (20 mL), recrystallized from acetone/water and dried in air.

3a · 0.5 H_2O [8]:

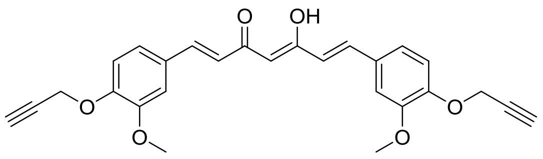

Yield 87%, orange solid. m. p.: 170 – 171 °C. ^{1}H-NMR (400 MHz, DMSO-d_6): δ 7.60 (d, 2H, *J* = 15.9 Hz); 7.39 (d, 2H, *J* = 1.9 Hz); 7.28 (dd, 2H, *J* = 8.3, 1.9 Hz); 7.09 (d, 2H, *J* = 8.3 Hz); 6.87 (d, 2H, *J* = 15.9 Hz); 6.13 (s, 1H); 4.86 (d, 4H, *J* = 2.6 Hz); 3.85 (s, 6H); 3.61 (t, 2H, *J* = 2.6 Hz). ^{13}C{^{1}H}-NMR (100 MHz, DMSO-d_6): δ 183.0; 149.4; 148.5; 140.0; 128.5; 122.5; 114.0; 111.2; 100.8; 78.8; 78.2; 56.1; 55.6. EI-MS: m/z (%) = 444 (30); 426 (25); 405 (8); 387 (38); 215 (100). UV/Vis λ_{max} [nm] (ε [$M^{-1}cm^{-1}$]): 414 (17254). Anal. calcd. for $C_{27}H_{24}O_6 \cdot 0.5H_2O$: C (71.51); H (5.56). Found: C (71.18); H (5.75).

3b · 0.25 H_2O:

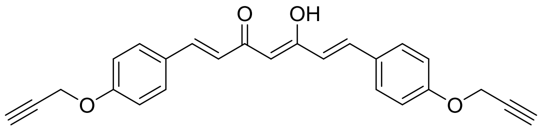

Yield 92%, brownish yellow solid. m. p.: 176 – 178 °C. ^{1}H-NMR (400 MHz, DMSO-d_6): δ 7.70 (d, 4H, *J* = 8.7 Hz); 7.59 (d, 2H, *J* = 15.9 Hz); 7.05 (d, 4H, *J* = 8.7 Hz); 6.81 (d, 2H, *J* = 8.7 Hz); 6.11 (s, 1H); 4.87 (d, 4H, *J* = 2.2 Hz); 3.60 (t, 2H, *J* = 2.2 Hz). ^{13}C{^{1}H}-NMR (100 MHz, DMSO-d_6): 183.2; 159.0; 139.9; 130.1; 128.0; 122.3; 115.4; 101.4; 79.0; 78.6; 55.6. EI-MS: m/z (%) = 384 (29); 366 (10); 345 (10); 185 (90); 180 (100). UV/Vis λ_{max} [nm] (ε [$M^{-1}cm^{-1}$]): 410 (41437). Anal. calcd. for $C_{25}H_{20}O_4 \cdot 0.25\ H_2O$: C (77.20); H (5.31). Found: C (77.57); H (5.18).

3c · 0.5 H_2O:

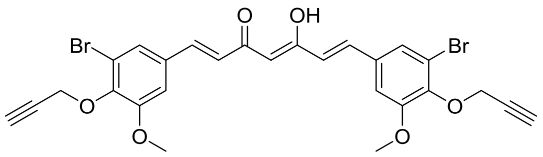

Yield 92%, yellow-orange solid. m. p.: 207.5 – 209.5 °C. ^{1}H-NMR (400 MHz, DMSO-d_6): δ 7.61 (d, 2H, *J* = 2.0 Hz); 7.58 (d, 4H, *J* = 15.9 Hz); 7.47 (d, 2H, *J* = 2.0 Hz); 7.02 (d, 4H, *J* = 15.8 Hz); 6.14 (s, 1H); 4.78 (d, 4H, *J* = 2.6 Hz); 3.90 (s, 6H); 3.54 (t, 2H, *J* = 2.4 Hz). ^{13}C{^{1}H}-NMR (100 MHz, DMSO-d_6): δ 183.1; 153.6; 144.6; 138.8; 132.7; 125.2; 124.6; 117.8; 112.0; 102.1; 78.9; 78.8; 59.7; 56.4. EI-MS: m/z (%) = 602 (18); 584 (10); 563 (17); 545 (35); 517 (13). UV/Vis λ_{max} [nm] (ε [$M^{-1}cm^{-1}$]): 405 (36739). Anal. calcd. for $C_{27}H_{22}Br_2O_6 \cdot 0.5H_2O$: C (53.05); H (3.79). Found: C (52.89); H (3.72).

3e · 0.5 H_2O:

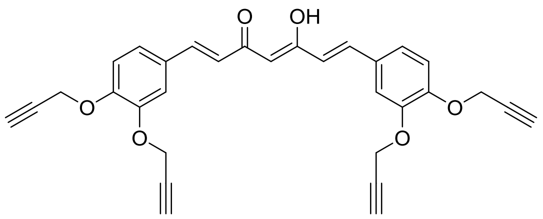

Yield 84%, yellow solid. m. p.: 153 – 154 °C. ^{1}H-NMR (400 MHz, DMSO-d_6): δ 7.58 (d, 2H, *J* = 15.8 Hz); 7.45 (d, 2H, *J* = 1.8 Hz); 7.33 (dd, 2H, *J* = 8.5 Hz, 1.7 Hz); 7.12 (d, 2H, *J* = 8.5 Hz); 6.84 (d, 2H, *J* = 15.9 Hz); 6.14 (s, 1H); 4.89 (d, 4H, *J* = 2.4 Hz); 4.88 (d, 4H, *J* = 2.3 Hz; 3.60 - 3.62 (m, 4H). ^{13}C{^{1}H}-NMR (100 MHz, DMSO-d_6): δ 183.2; 148.9; 147.0; 140.1; 128.2; 123.3; 122.7; 113.9; 113.0; 101.1; 79.0; 78.9; 78.7; 78.6; 56.1; 56.0. EI-MS: m/z (%) = 492 (20); 474 (8); 453 (17); 239 (100). UV/Vis λ_{max} [nm] (ε [$M^{-1}cm^{-1}$]): 405 (50732). Anal. calcd. for $C_{31}H_{24}O_6 \cdot 0.5H_2O$: C (74.24); H (5.02). Found: C (74.11); H (4.93).

3f · 0.6 H_2O:

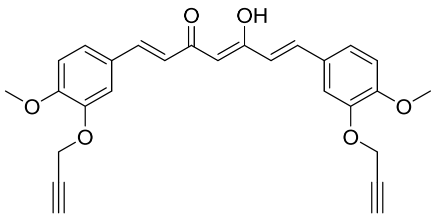

Yield 90%, yellow-orange solid. m. p.: 176 – 177 °C. ^{1}H-NMR (400 MHz, DMSO-d_6): δ 7.58 (d, 2H, *J* = 15.9 Hz); 7.42 (d, 2H, *J* = 1.8 Hz); 7.32 (dd, *J* = 8.4 Hz, 1.7 Hz); 7.05 (d, 2H, *J* = 8.5 Hz); 6.80 (d, 2H, *J* = 15.9 Hz); 6.12 (s, 1H); 4.87 (d, 4H, *J* = 2.3 Hz); 3.82 (s, 6H); 3.57 (t, 2H, *J* = 2.3 Hz). ^{13}C{^{1}H}-NMR: (400 MHz, DMSO-d_6): δ = 183.2; 151.4; 146.7; 140.3; 127.4; 123.9; 122.3; 112.7; 112.2; 101.0; 79.2; 78.5; 56.1; 55.7. EI-MS: m/z (%) 444 (19); 426 (17); 405 (10); 215 (60); 188 (100); 173 (75). UV/Vis λ_{max} [nm] (ε [$M^{-1}cm^{-1}$]): 418 (25029). Anal. calcd. for $C_{27}H_{24}O_6 \cdot 0.6H_2O$: C (71.23); H (5.58). Found: C (71.03); H (5.26).

3g · 0.5 H_2O:

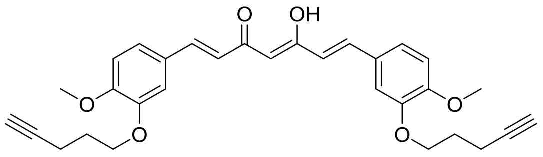

Yield 80%, yellow-orange solid. m. p.: 132 – 133 °C. ^{1}H-NMR (400 MHz, DMSO-d_6): δ = 7.56 (d, 2H, *J* = 15.6 Hz); 7.35 (d, 2H, *J* = 2.1 Hz); 7.26 (dd, 2H, *J* = 8.4, 2.1 Hz); 7.01 (d, 2H, *J* = 8.4 Hz); 6.81 (d, 2H, *J* = 15.8 Hz); 6.10 (s, 1H); 4.09 (t, 4H, *J* = 6.2 Hz); 3.81 (s, 6H); 2.82 (t, 2H, *J* = 2.7 Hz); 2.34 (td, 2H, *J* = 7.1, 2.7 Hz); 1.89 – 1.93 (m, 4H). ^{13}C{^{1}H}-NMR (100 MHz, DMSO-d_6): δ = 183.2; 151.2; 148.2; 140.3; 127.6; 123.1; 122.1; 111.9; 111.8; 83.7; 71.6; 66.8; 55.7; 27.8; 14.5. EI-MS: m/z (%) = 500 (49); 482 (100); 467; 404 (37); 256 (47); 232 (70); 203 (31); 178 (55). UV/Vis λ_{max} [nm] (ε [$M^{-1}cm^{-1}$]): 405 (11036). Anal. calcd. for $C_{31}H_{32}O_6{\cdot}0.5H_2O$: C (73.07); H (6.53). Found: C (73.15); H (6.47).

3h:

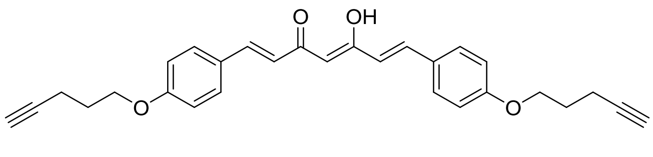

Yield 95%, orange solid. m. p.: 153.5 – 154.5 °C. ^{1}H-NMR (400 MHz, DMSO-d_6): δ = 16.32 (br s, 1H); 7.67 (d, 4H, *J* = 8.8 Hz); 7.59 (d, 2H, *J* = 15.9 Hz); 7.00 (d, 4H, *J* = 8.8 Hz); 6.78 (d, 2H, *J* = 15.9 Hz); 6.08 (s, 1H); 4.09 (t, 4H, *J* = 6.2 Hz); 2.82 (t, 2H, *J* = 2.6 Hz); 2.34 (td, 4H, *J* = 7.1, 2.6 Hz); 2.34 (td, 2H, *J* = 7.1, 2.7 Hz); 1.90 (qui, 4H, *J* = 6.7 Hz). ^{13}C{^{1}H}-NMR (100 MHz, DMSO-d_6): δ = 183.2; 160.3; 140.0; 130.2; 127.4; 121.9; 114.9; 101.2; 83.6; 71.7; 66.2; 27.6; 14.4. EI-MS: m/z (%) = . UV/Vis λ_{max} [nm]

(ε [$M^{-1}cm^{-1}$]): 414 (29870). Anal. calcd. for $C_{29}H_{28}O_4$: C (79.07); H (6.41). Found: C (78.86); H (3.36).

5. X-ray crystallographic data for 2f, 2g and 2h

The intensity data for the compounds were collected on a Nonius KappaCCD diffractometer using graphite-monochromated Mo-Kα radiation. Data were corrected for Lorentz and polarization effects; absorption was taken into account on a semi-empirical basis using multiple-scans [9-11]. The structures were solved by direct methods (SHELXS [12]) and refined by full-matrix least squares techniques against Fo2 (SHELXL-97 [13]). All hydrogen atoms were located by difference Fourier synthesis and refined isotropically. All non-hydrogen atoms were refined anisotropically [12]. Crystallographic data as well as structure solution and refinement details are summarized in Table S1. MERCURY [13] was used for structure representations.

Table S1: Crystal data and refinement details for the X-ray structure determinations of the compounds **2f–2h.**

Compound	**2f**	**2g**	**2h**
formula	$C_{27}H_{23}BF_2O_6$	$C_{31}H_{31}BF_2O_6$	$C_{29}H_{27}BF_2O_4$
fw (g·mol^{-1})	492.26	548.37	488.32
T/°*C*	-140(2)	-140(2)	-140(2)
crystal system	triclinic	triclinic	monoclinic
space group	P ī	P ī	C 2/c
a/ Å	8.5535(3)	11.1586(3)	10.7368(3)
b/ Å	10.2782(3)	11.2846(3)	16.3081(5)
c/ Å	14.3695(5)	11.7934(3)	14.5077(5)
α/°	101.013(2)	88.864(2)	90
β/°	102.402(2)	83.361(2)	102.498(2)
γ/°	101.684(2)	69.043(2)	90
V/Å^3	1171.36(7)	1377.12(6)	2480.06(13)
Z	2	2	4
ρ (g·cm^{-3})	1.396	1.322	1.308
μ (cm^{-1})	1.07	.99	.95
measured data	7699	17970	8445
data with I > 2σ(I)	3677	5030	2349
unique data (R_{int})	5140/0.0384	6282/0.0289	2741/0.0321
wR_2 (all data, on F^2)[a)]	0.1551	0.1180	0.1189
R_1 ($I > 2\sigma(I)$) [a)]	0.0778	0.0494	0.0456
S [b)]	1.168	1.050	1.067
Res. dens./e·Å^{-3}	0.310/-0.310	0.326/-0.208	0.432/-0.223
absorpt method	multi-scan	multi-scan	multi-scan
absorpt corr $T_{min}/_{max}$	0.6526/0.7456	0.6925/0.7456	0.6783/0.7456
CCDC No.	1526555	1526556	1526557

a) Definition of the *R* indices: $R_1 = (\Sigma || F_o| - |F_c||)/\Sigma |F_o|$;
$wR_2 = \{\Sigma[w(F_o^2-F_c^2)^2]/\Sigma[w(F_o^2)^2]\}^{1/2}$ with $w^{-1} = \square^2(F_o^2) + (aP)^2+bP$; $P = [2F_c^2 + Max(F_O^2]/3$;
b) $s = \{\Sigma[w(F_o^2-F_c^2)^2]/(N_o-N_p)\}^{1/2}$.

6. References:

1. Nakhjiri, M.; Safavi, M.; Alipour, E.; Emami, S.; Atash, A. F.; Jafari-Zavareh, M.; Ardestani, S. K.; Khoshneviszadeh, M.; Foroumadi, A.; Shafiee, A. *Eur. J. Med. Chem.* **2012,** *50*, 113–123.
2. Williams, A.; Winfield, S. *Analyst* **1983**, *108*, 1067–1071.
3. DecayFit - Fluorescence Decay Analysis Software 1.3, FluorTools, www.fluortools.com
3. Pal, M.; Parasuraman, K.; Yeleswarapu, K. R. *Org. Lett.* **2003,** *5*, 349–352.
4. Kant, R.; Kumar, D.; Agarwal, D.; Gupta, R. D.; Tilak, R.; Awasthi, S. K.; Agarwal, A. *Eur. J. Med. Chem.* **2016,** *113*, 34–49.
5. Zammit, S. C.; Cox, A. J.; Gow, R. M.; Zhang, Y.; Gilbert, R. E.; Krum, H.; Kelly, D. J.; Williams, S. J. *Bioorg. Med. Chem. Lett.* **2009,** *19*, 7003–7006.
6. Goto, H.; Nimori, S.; Akagi, K. *Synt. Met.* **2005,** *155*, 576–587.
7. Liu, K.; Chen, J.; Chojnacki, J.; Zhang, S. *Tetrahedron Lett.* **2013,** *54*, 2070–2073.
8. Gomes, D.; Alegrio, L.; Lim, M.; Leon, L.; Araújo, C. *Arzneim. Forsch.* **2002,** *52*, 120–124.
9. *COLLECT*, Data Collection Software; Nonius B. V., Netherlands, 1998.
10. Otwinowski, Z., Minor, W. Processing of X-Ray Diffraction Data Collected in Oscillation Mode. In *Methods in Enzymology*; Carter, C. W., Sweet, R. M., Eds.; Macromolecular Crystallography, Part A, Vol. 276, Academic Press, 1997, pp. 307-326. doi:10.1016/S0076-6879(97)76066-X
11. *SADABS 2.10*, Bruker-AXS inc., Madison, WI, U.S.A., 2002.
12. Sheldrick, G. M. *Acta Cryst.* **2008**, *A64*, 112–122. doi:10.1107/S0108767307043930
13. Macrae C. F.; Edgington P. R.; McCabe P.; Pidcock E.; Shields G. P.; Taylor R., Towler M., van de Streek J. *J. Appl. Cryst.* **2006**, *39*, 453. doi:10.1107/S002188980600731X

7. Intermolecular interactions in 2f:

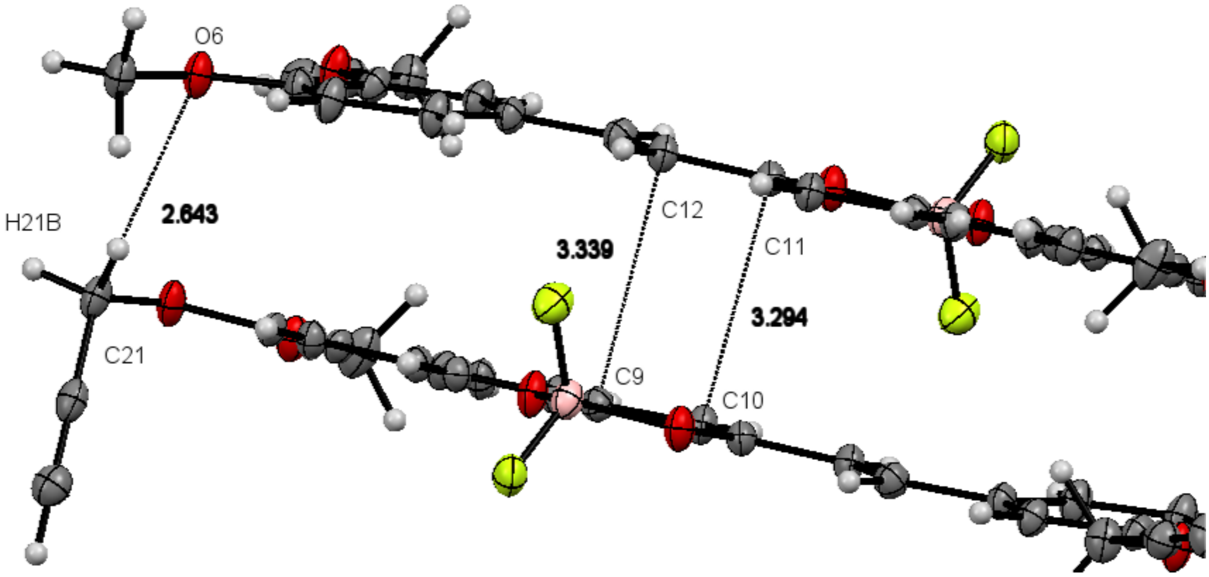

Figure S1: ORTEP drawing showing intermolecular interactions between two molecules of **2f**. Calculated distances are given in Å.

8. NMR spectra for compounds 2 and 3

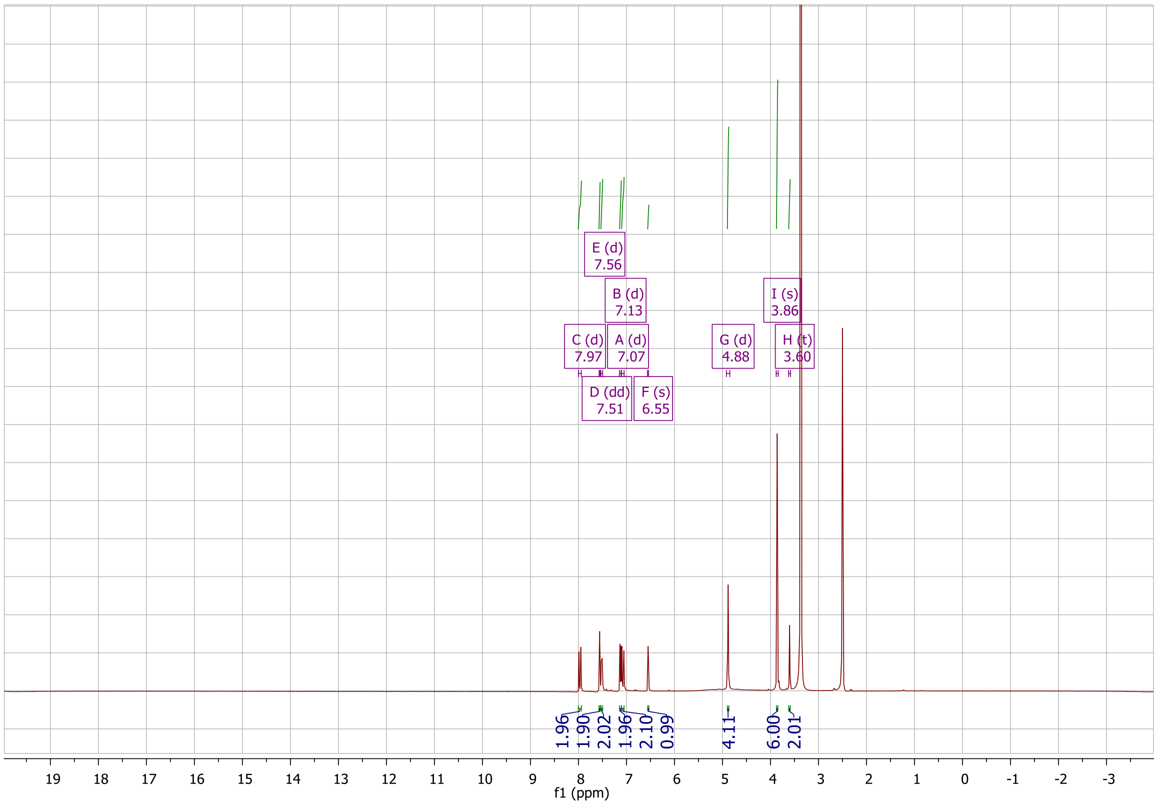

Figure S2: ^{1}H NMR spectrum (400 MHz, DMSO-d_6) of **2a**.

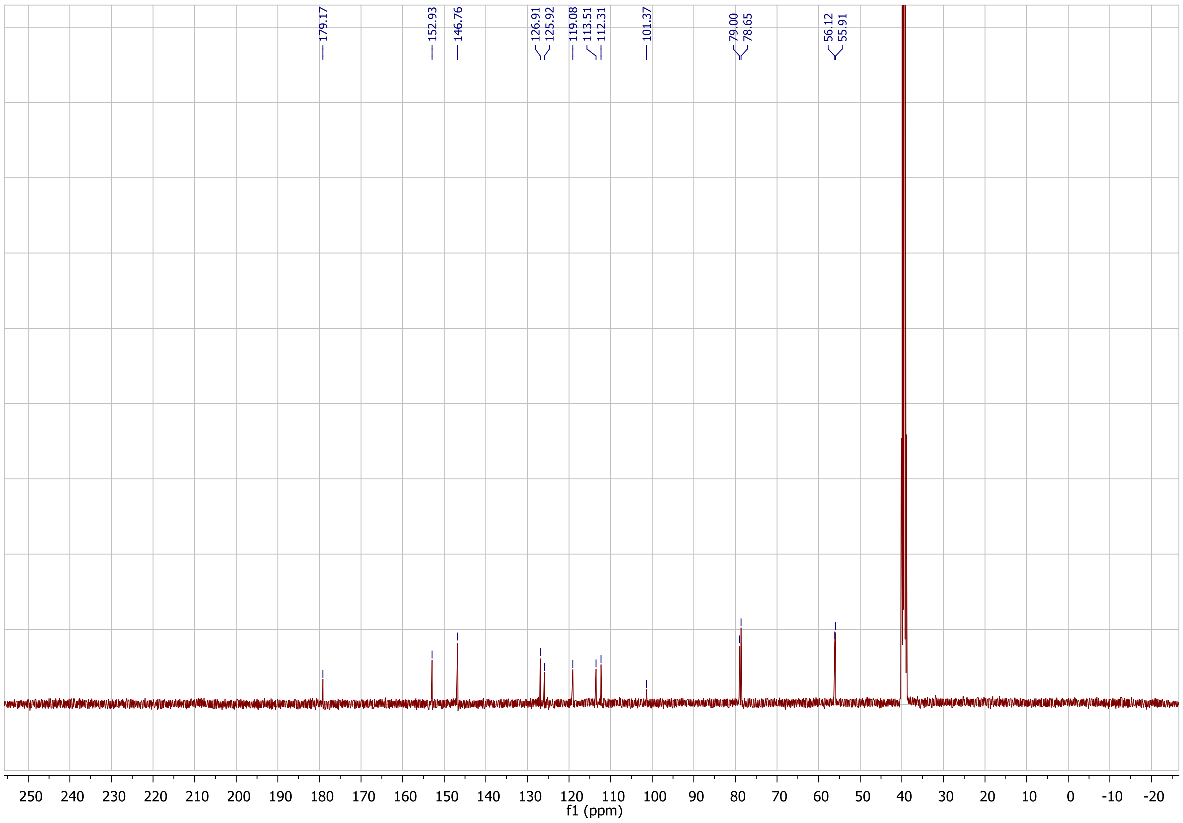

Figure S3: ^{13}C{^{1}H} NMR spectrum (100 MHz, DMSO-d_6) of **2a**.

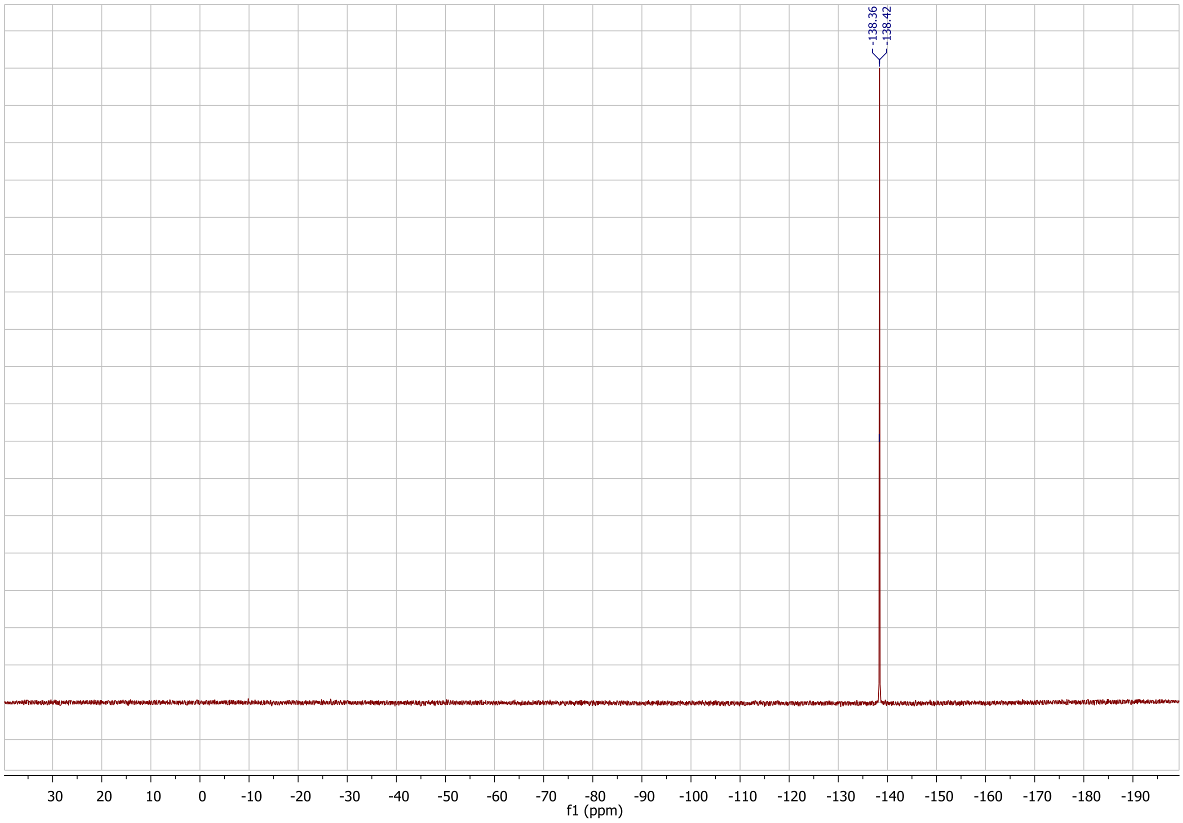

Figure S4: $^{19}F\{^{1}H\}$ NMR spectrum (188 MHz, DMSO-d_6) of **2a**.

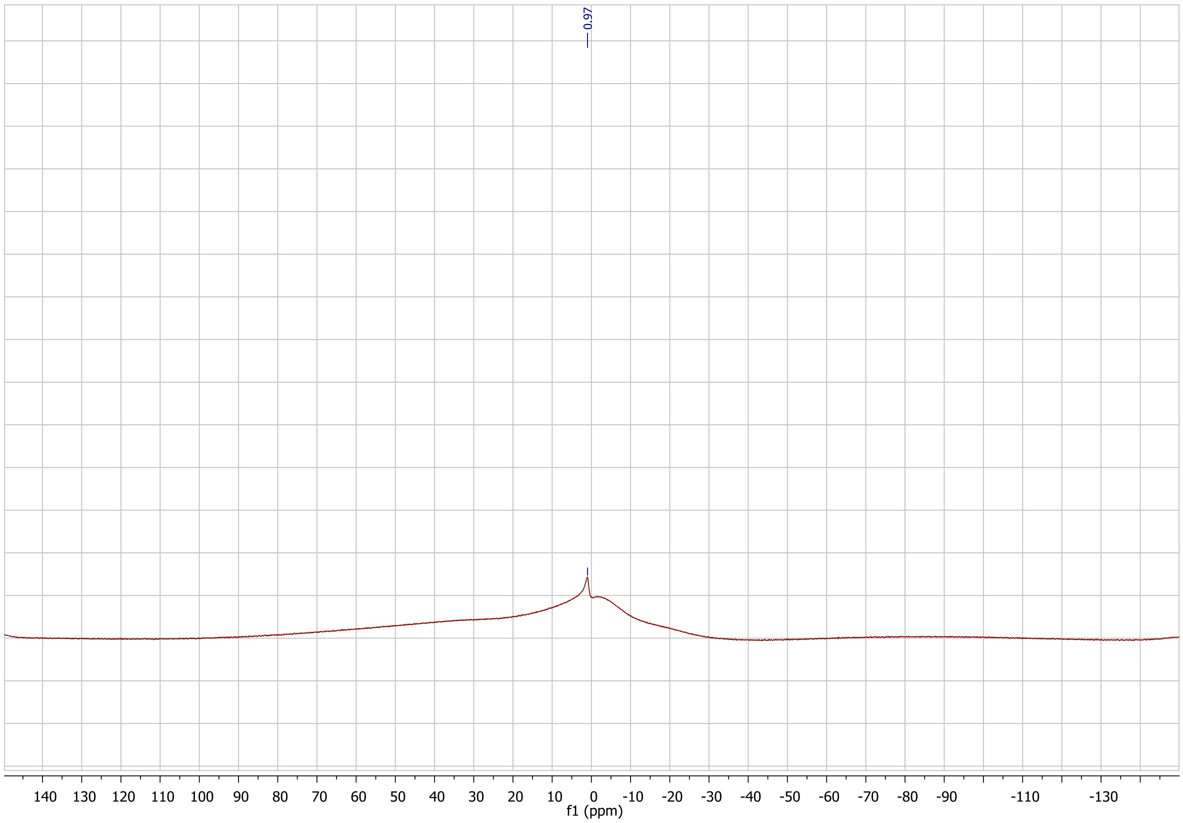

Figure S5: $^{11}B\{^{1}H\}$ NMR spectrum (128 MHz, DMSO-d_6) of **2a**.

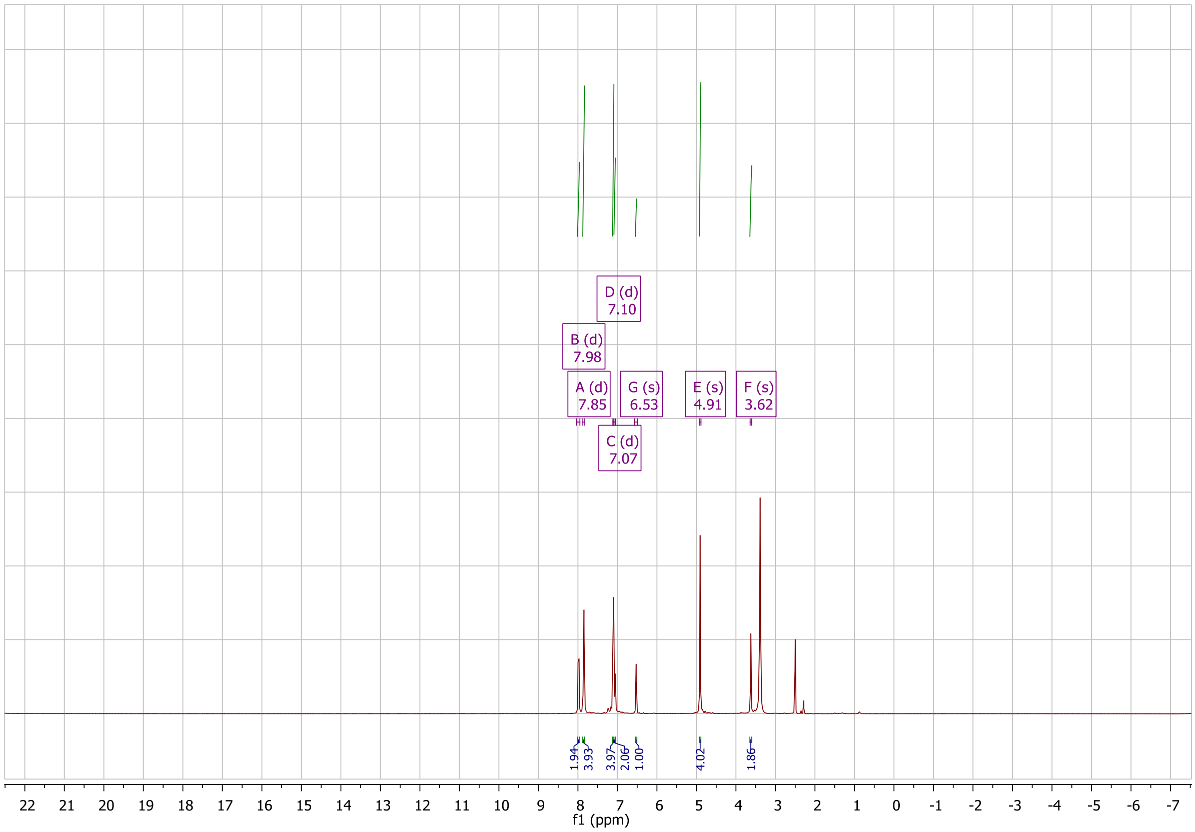

Figure S6: ^{1}H NMR spectrum (400 MHz, DMSO-d_6) of **2b**.

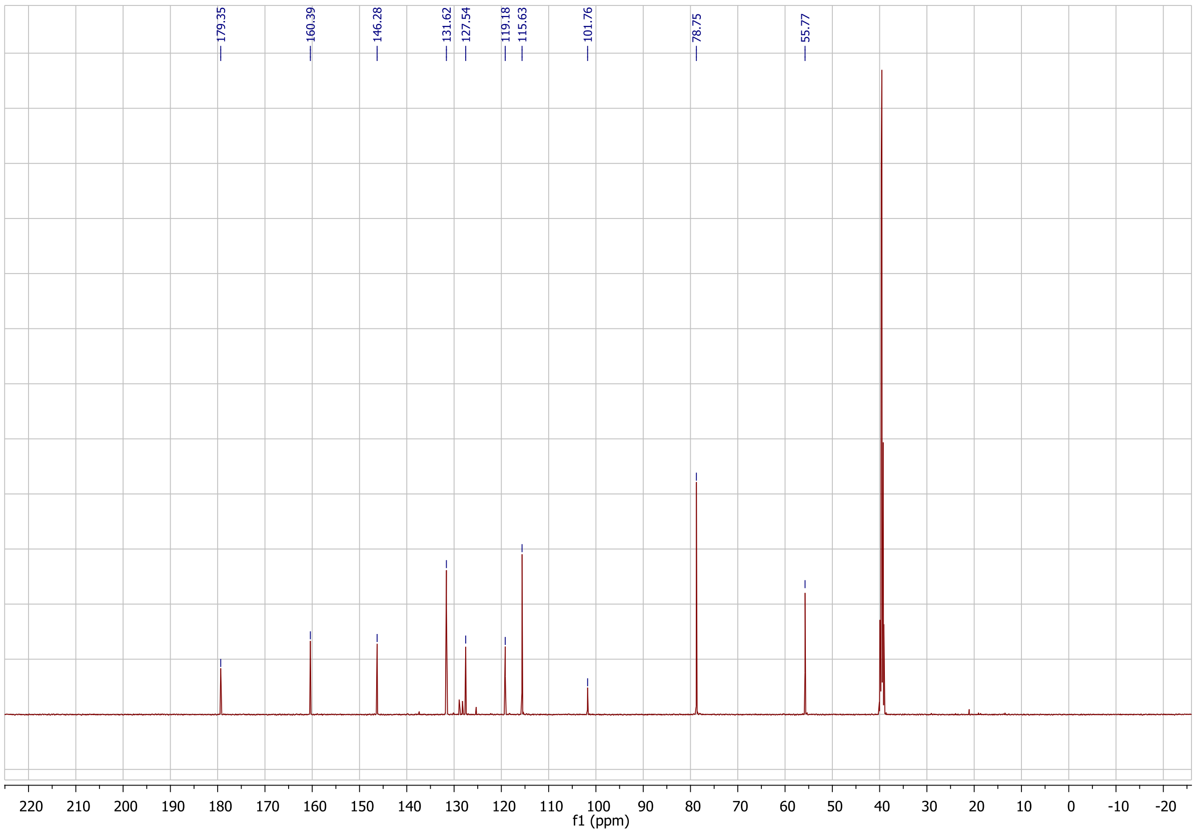

Figure S7: $^{13}C\{^{1}H\}$ NMR spectrum (100 MHz, DMSO-d_6) of **2b**.

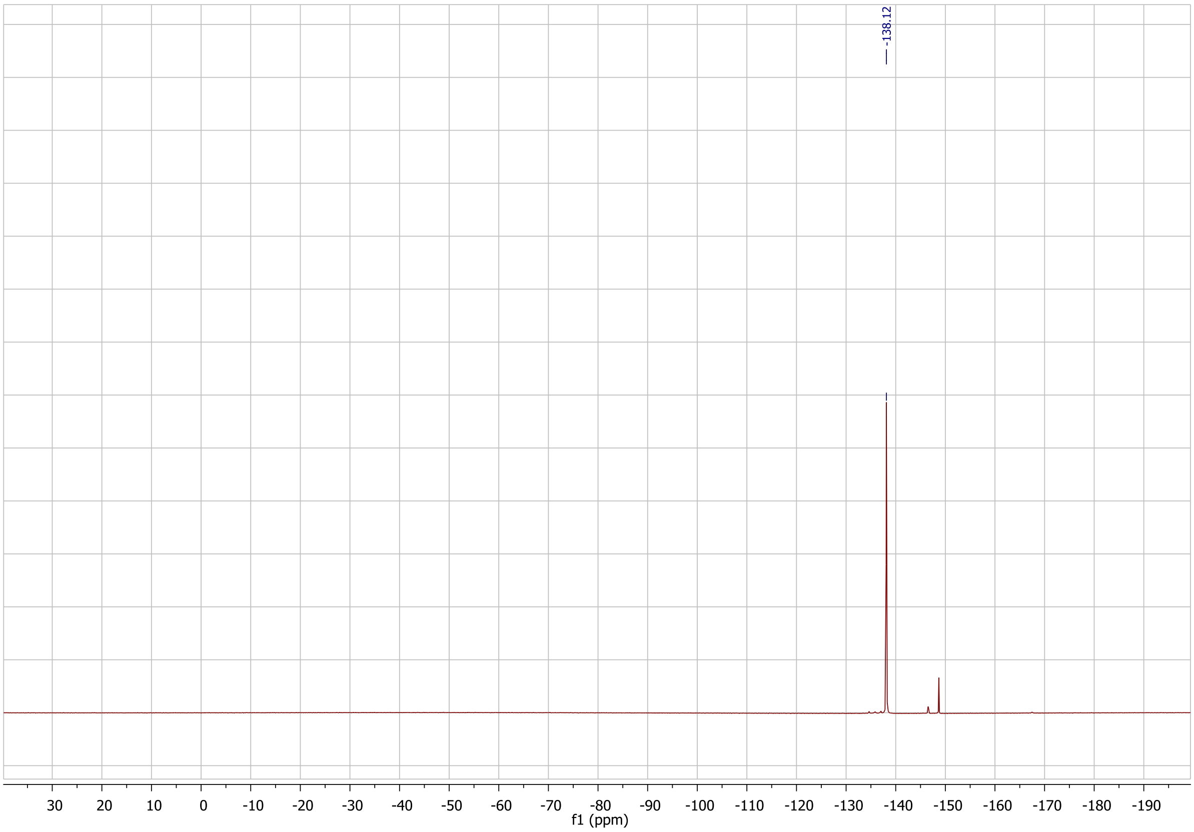

Figure S8: $^{19}F\{^1H\}$ NMR spectrum (188 MHz, DMSO-d_6) of **2b**.

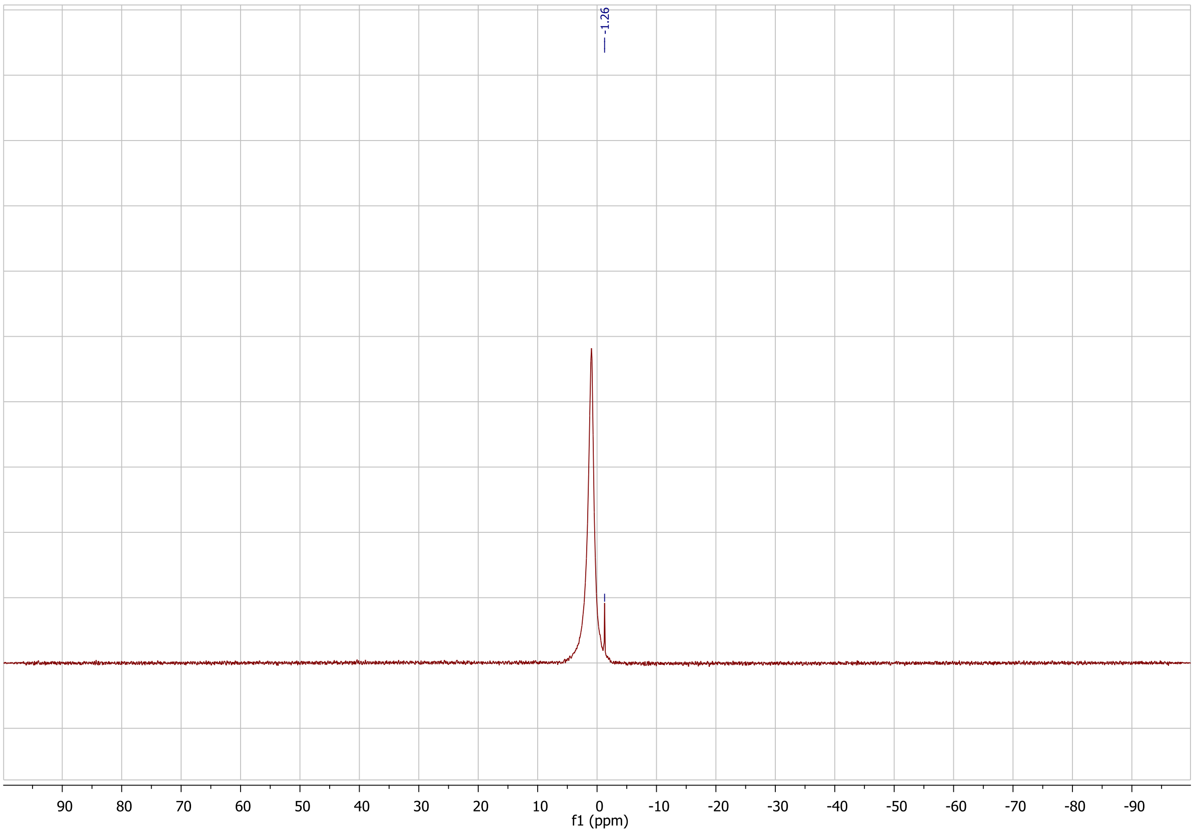

Figure S9: $^{11}B\{^1H\}$ NMR spectrum (128 MHz, DMSO-d_6) of **2b**.

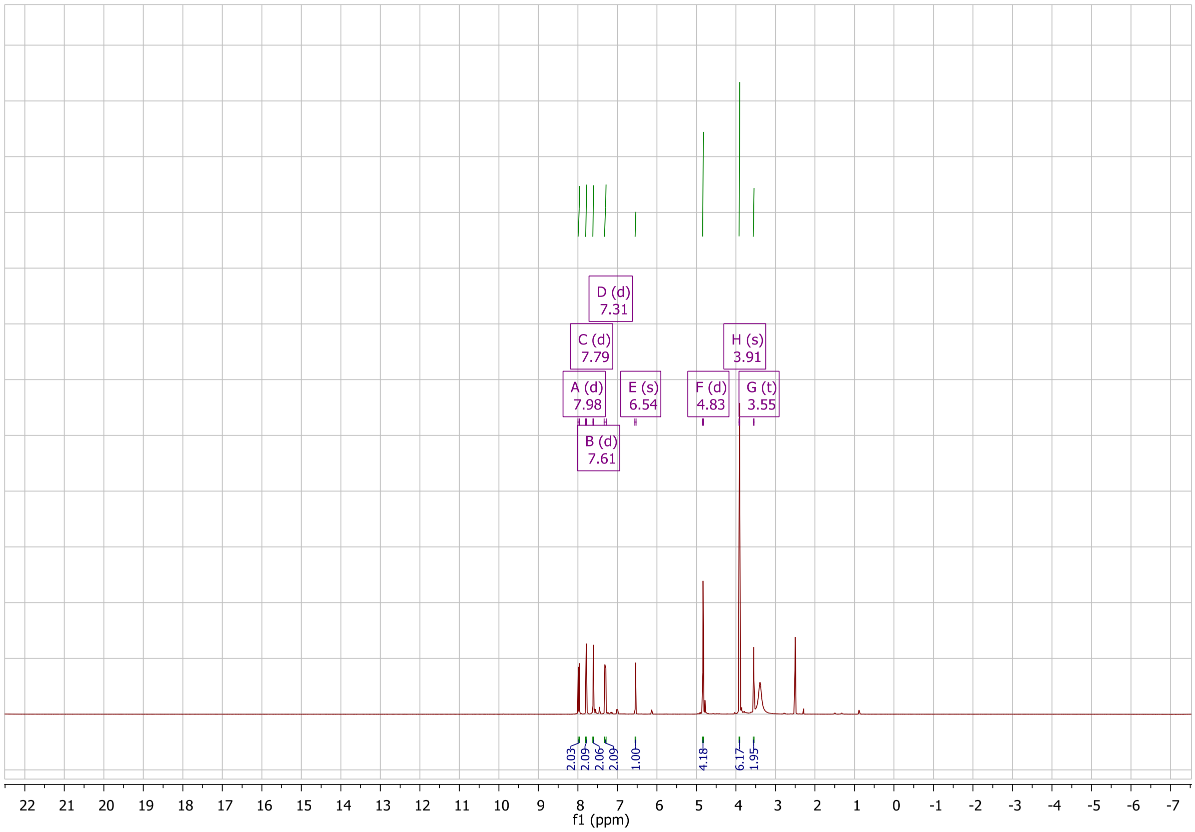

Figure S10: ^{1}H NMR spectrum (600 MHz, DMSO-d_6) of **2c**.

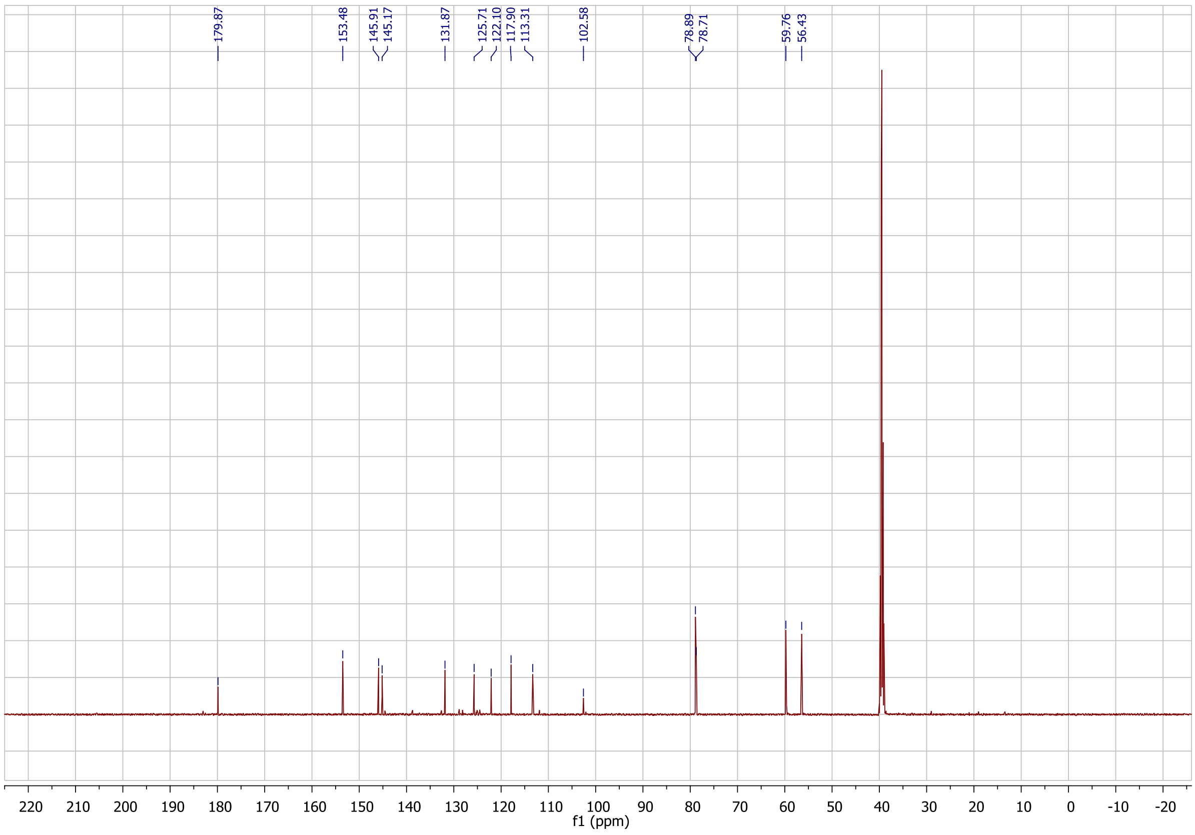

Figure S11: $^{13}C\{^{1}H\}$ NMR spectrum (150 MHz, DMSO-d_6) of **2c**.

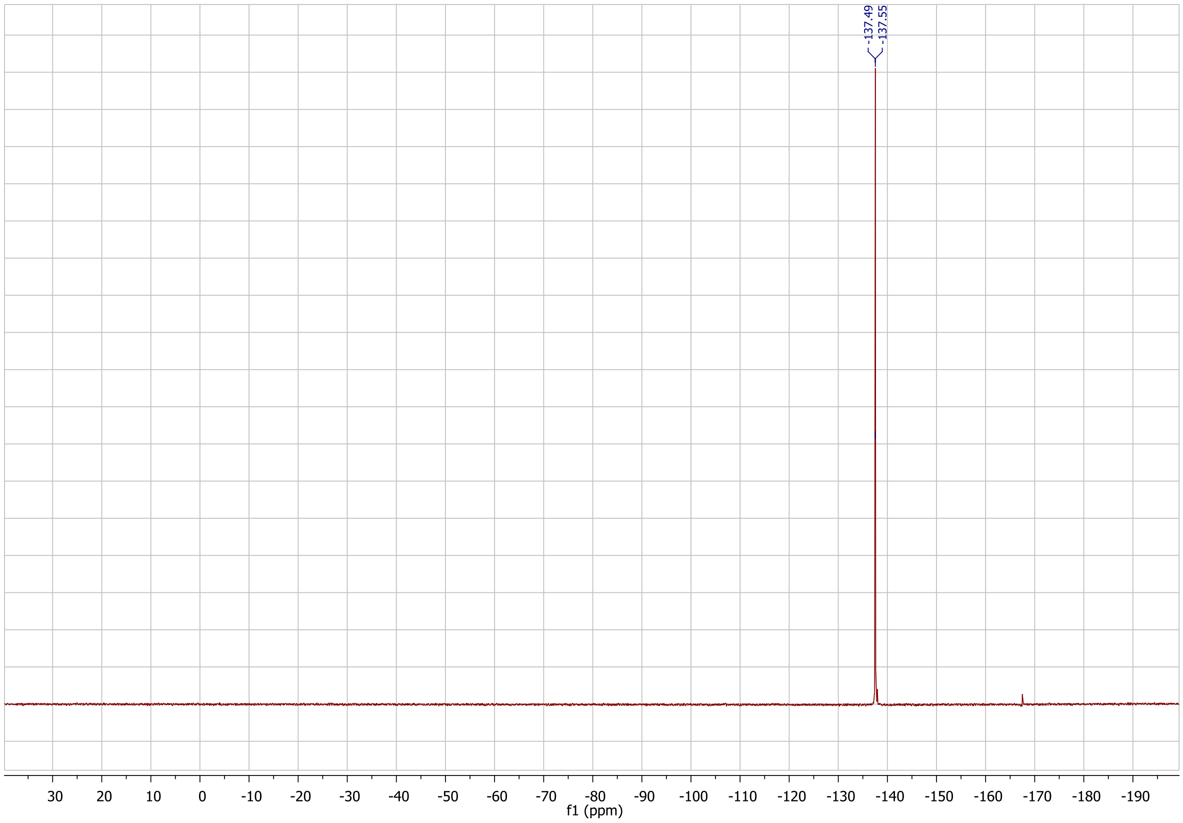

Figure S12: $^{19}F\{^{1}H\}$ NMR spectrum (188 MHz, DMSO-d_6) of **2c**.

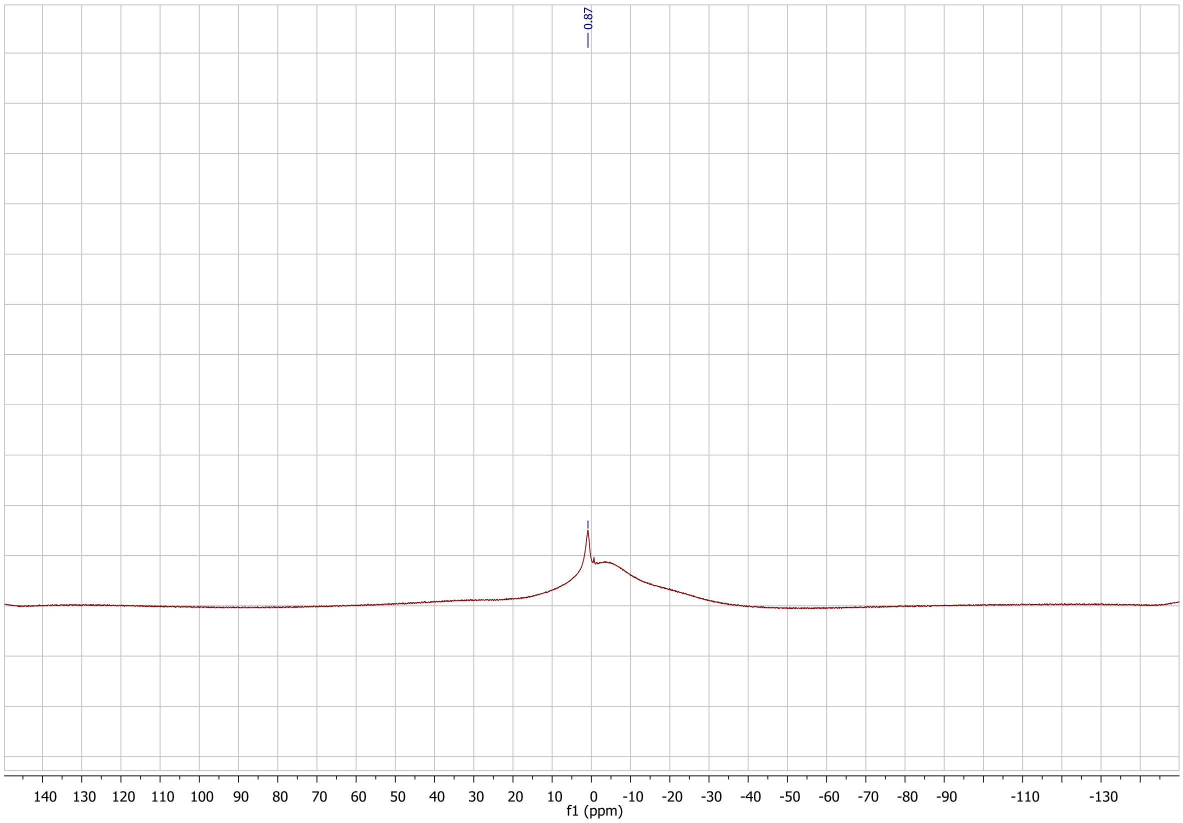

Figure S13: $^{11}B\{^{1}H\}$ NMR spectrum (128 MHz, DMSO-d_6) of **2c**.

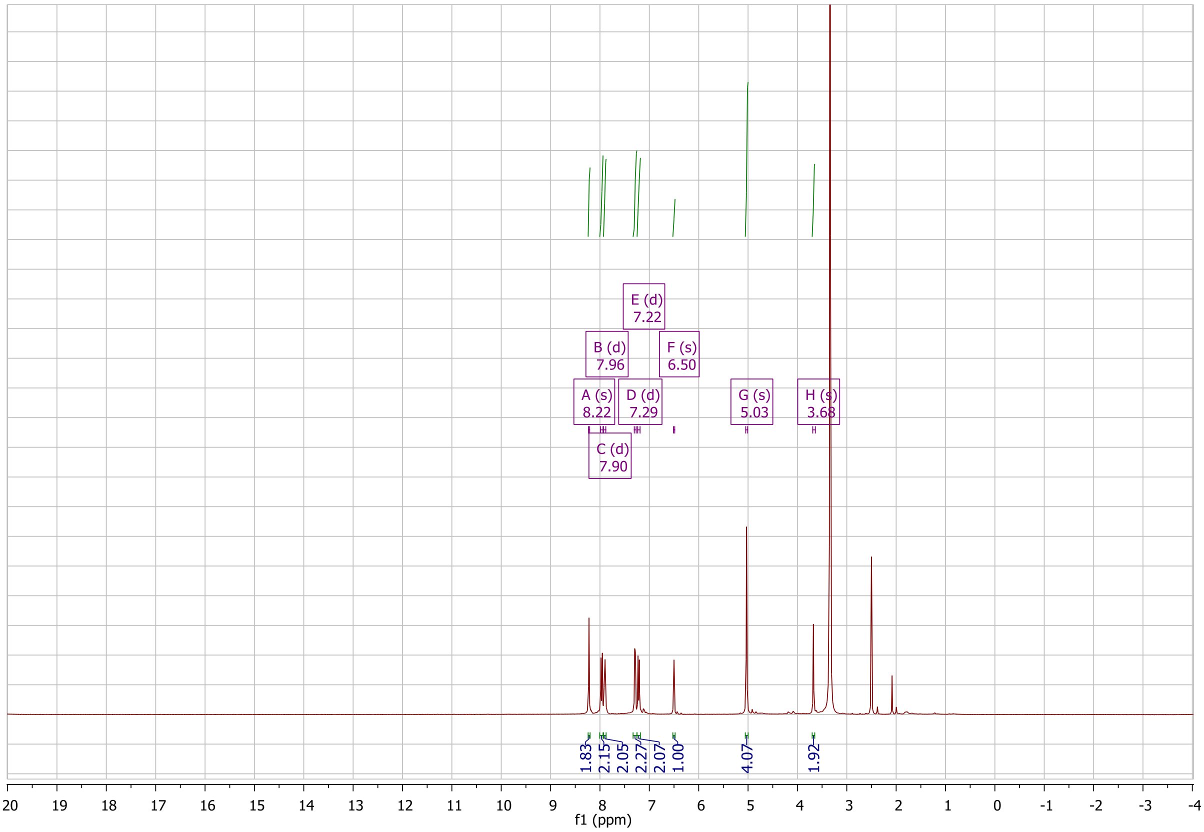

Figure S14: ^{1}H NMR spectrum (600 MHz, DMSO-d_6) of **2d**.

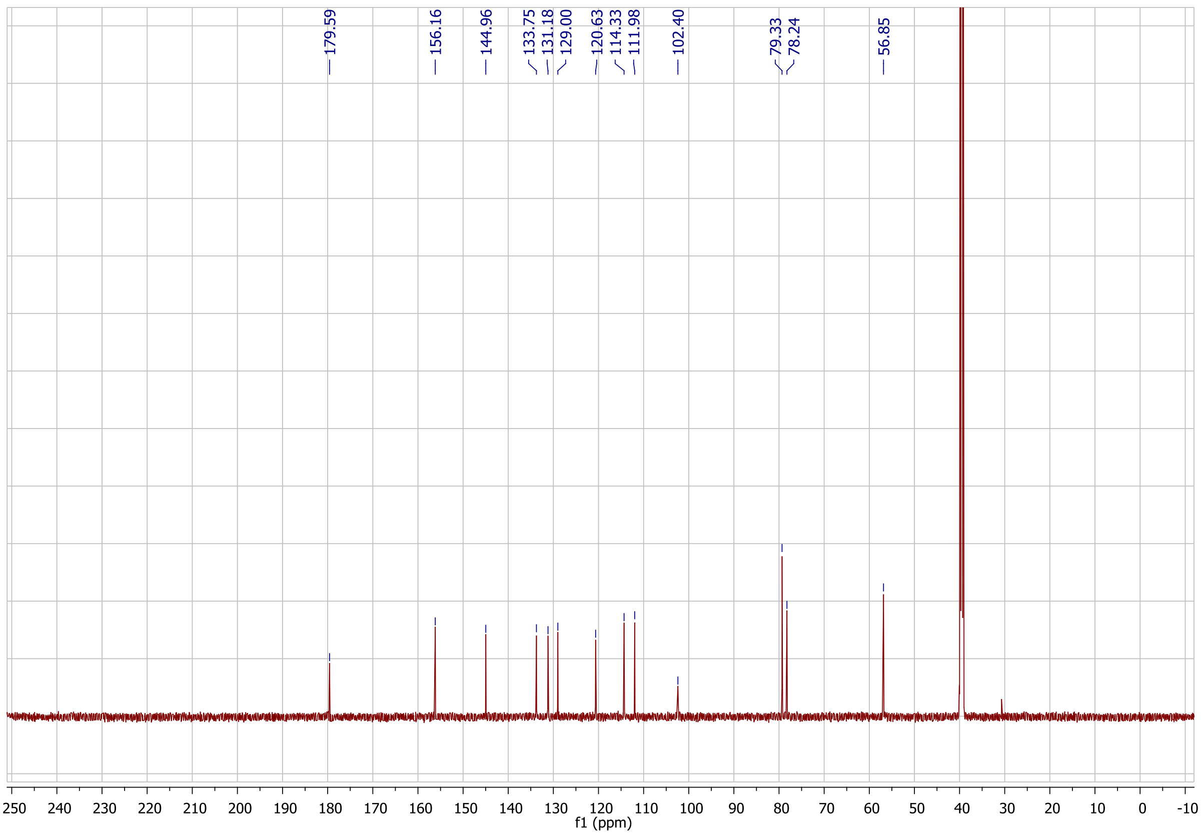

Figure S15: $^{13}C\{^{1}H\}$ NMR spectrum (150 MHz, DMSO-d_6) of **2d**.

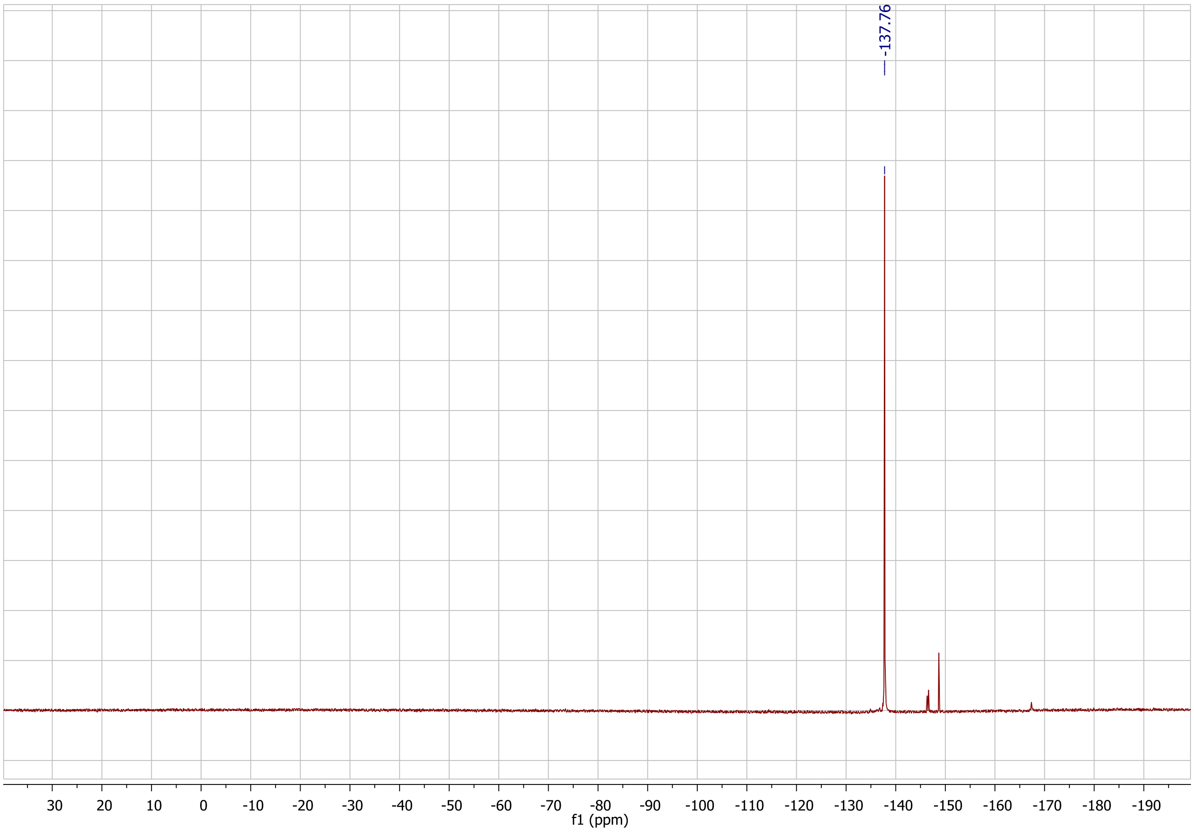

Figure S16: $^{19}F\{^{1}H\}$ NMR spectrum (188 MHz, DMSO-d_6) of **2d**.

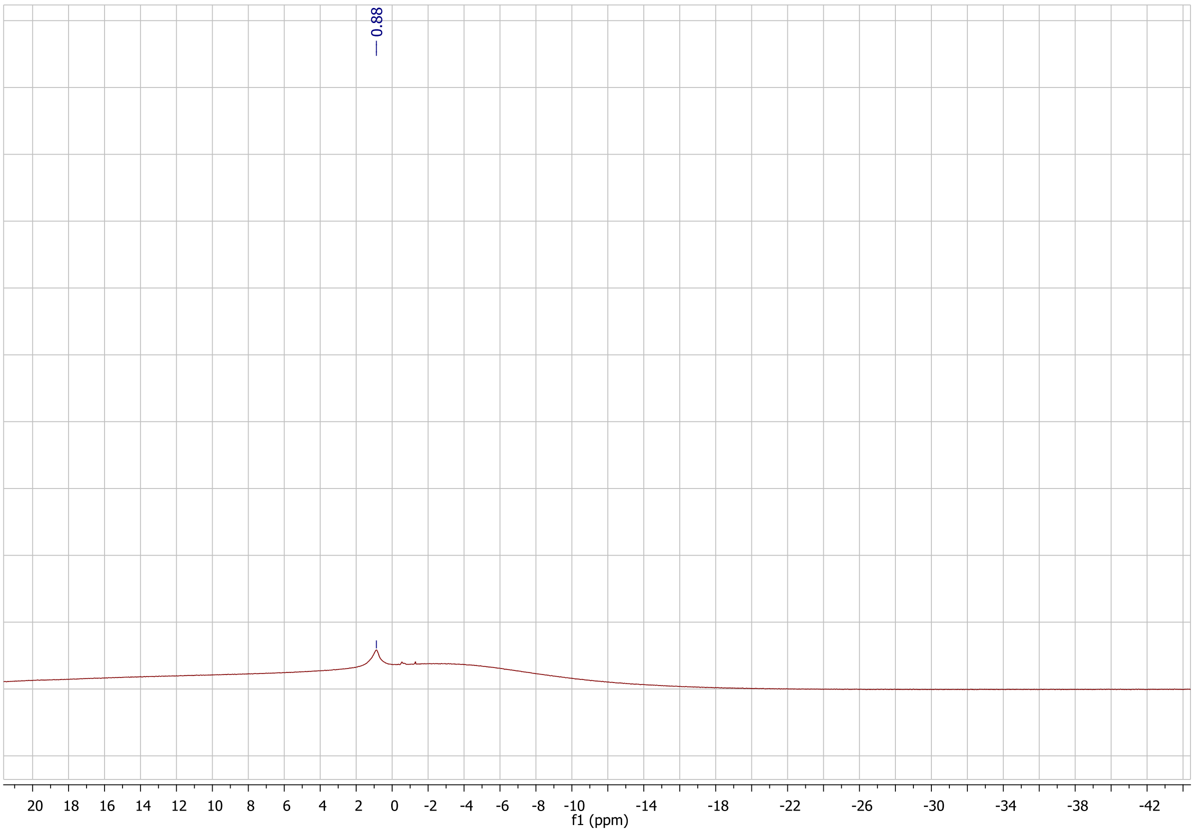

Figure S17: $^{11}B\{^{1}H\}$ NMR spectrum (128 MHz, DMSO-d_6) of **2d**.

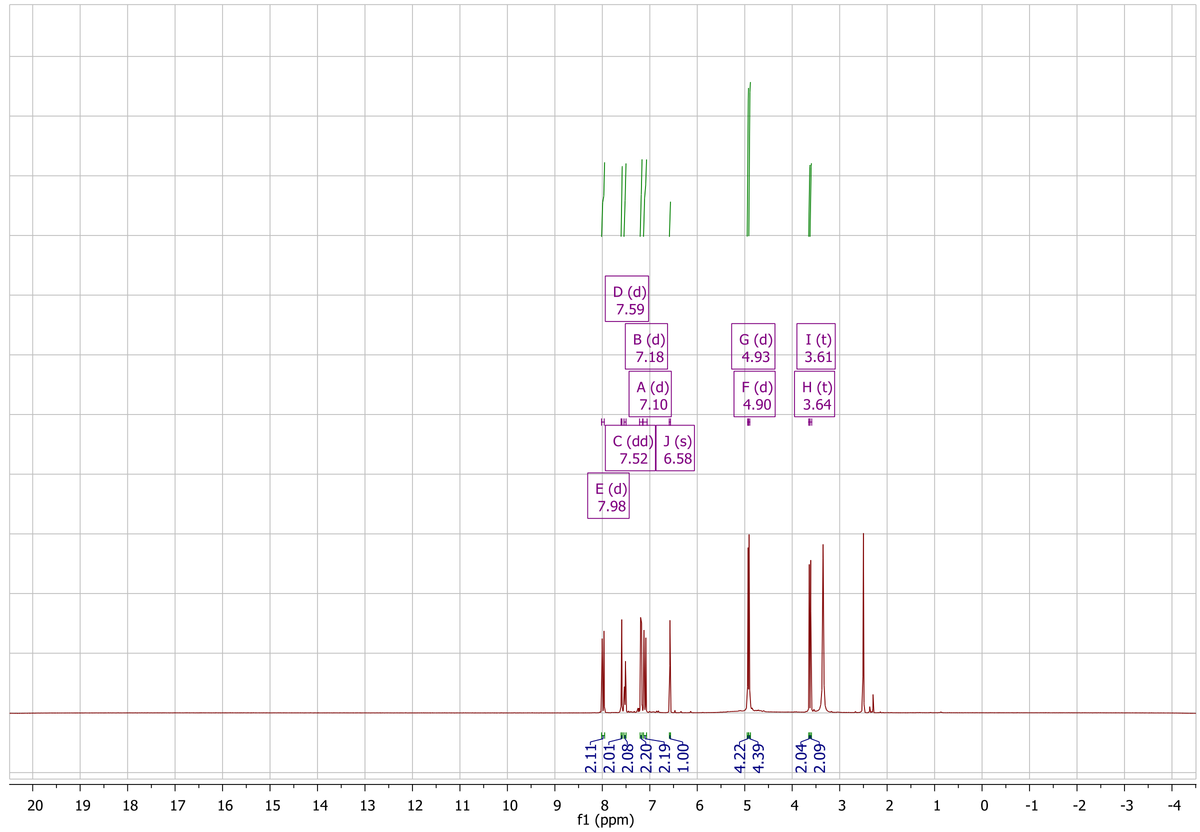

Figure S18: ^{1}H NMR spectrum (400 MHz, DMSO-d_6) of **2e**.

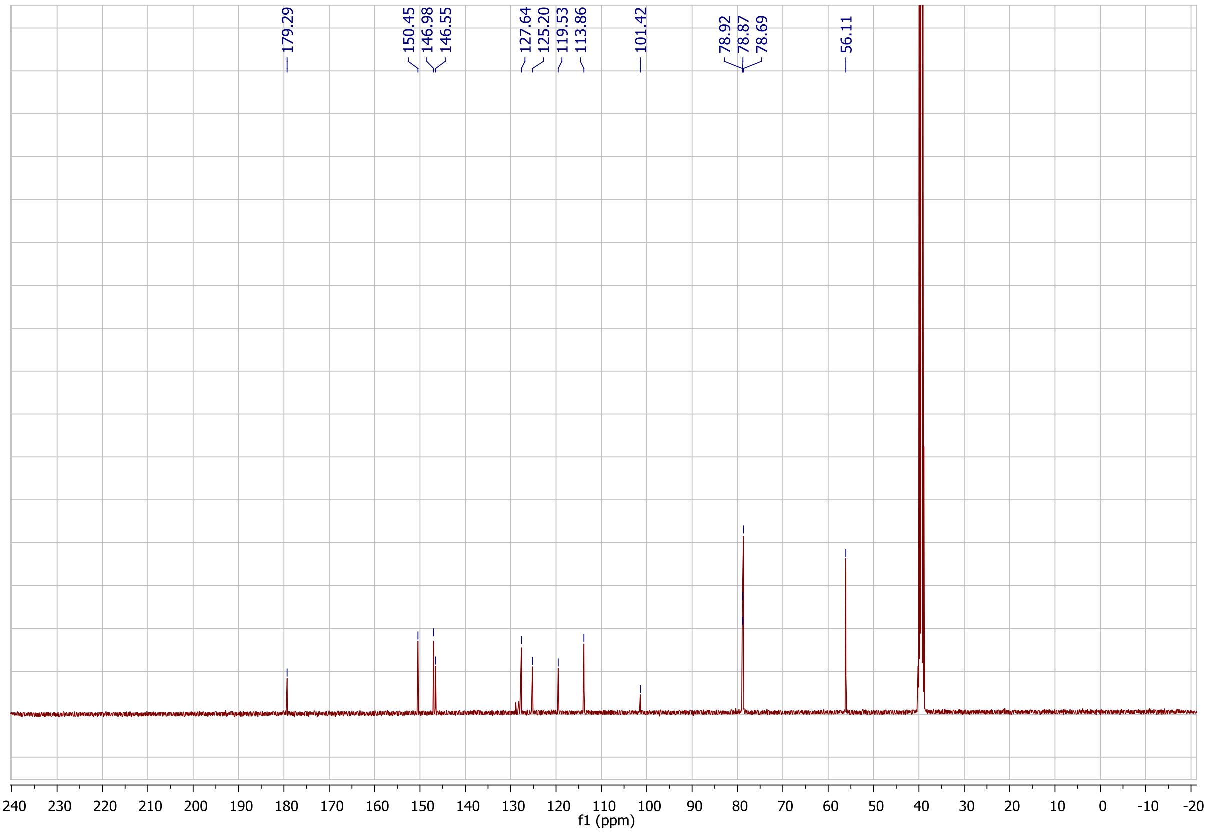

Figure S19: $^{13}C\{^{1}H\}$ NMR spectrum (100 MHz, DMSO-d_6) of **2e**.

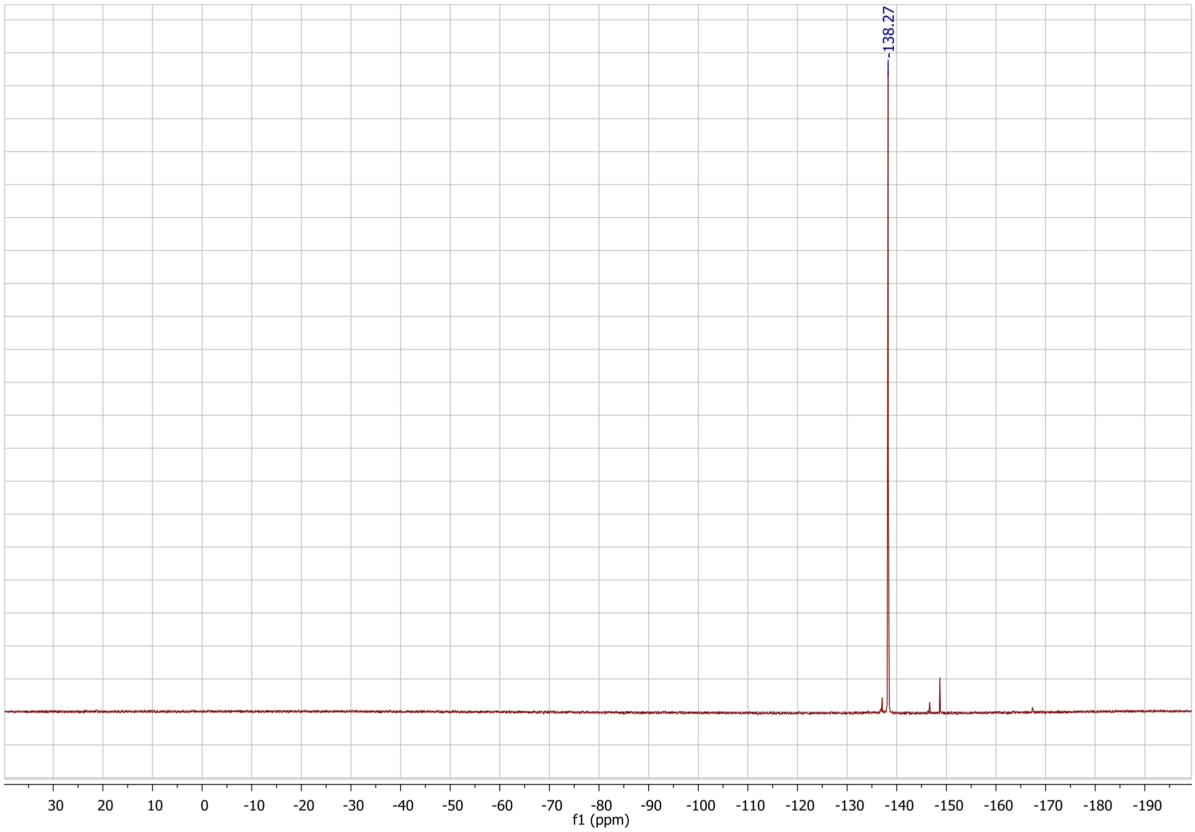

Figure S20: $^{19}F\{^{1}H\}$ NMR spectrum (188 MHz, DMSO-d_6) of **2e**.

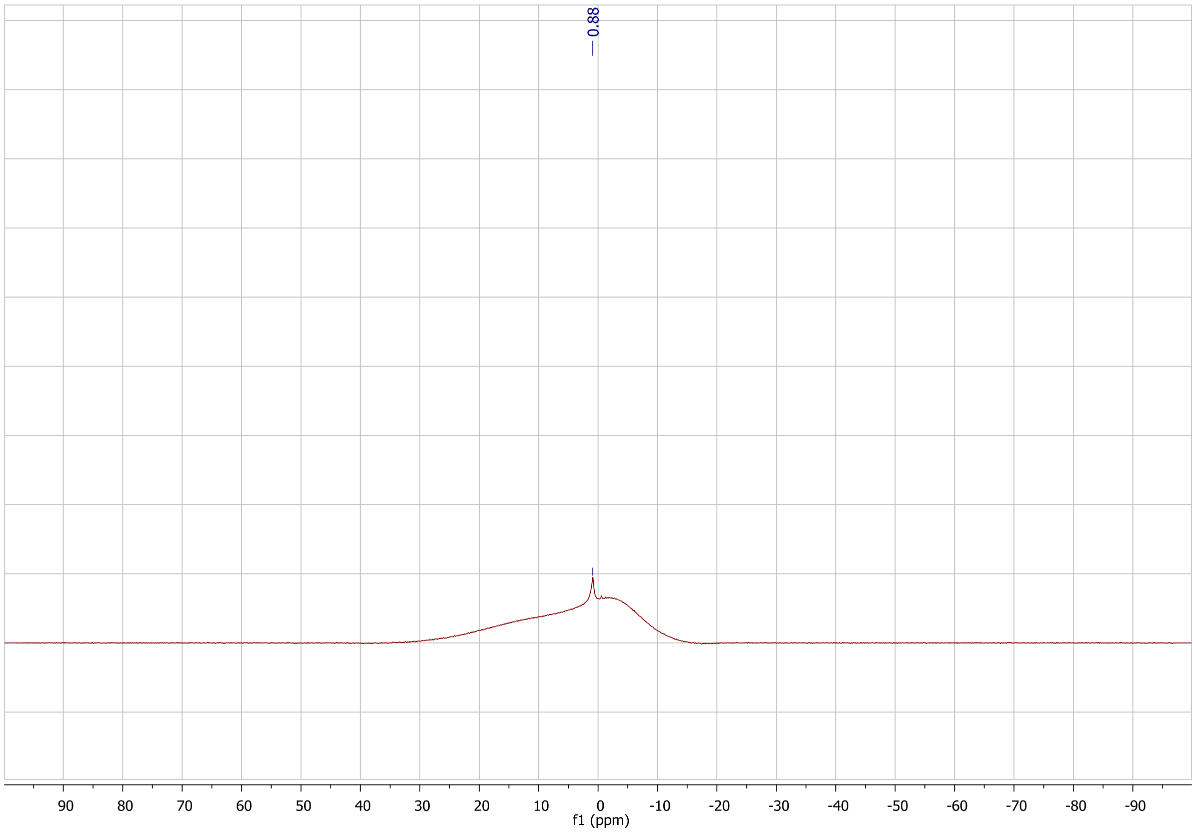

Figure S21: $^{11}B\{^{1}H\}$ NMR spectrum (128 MHz, DMSO-d_6) of **2e**.

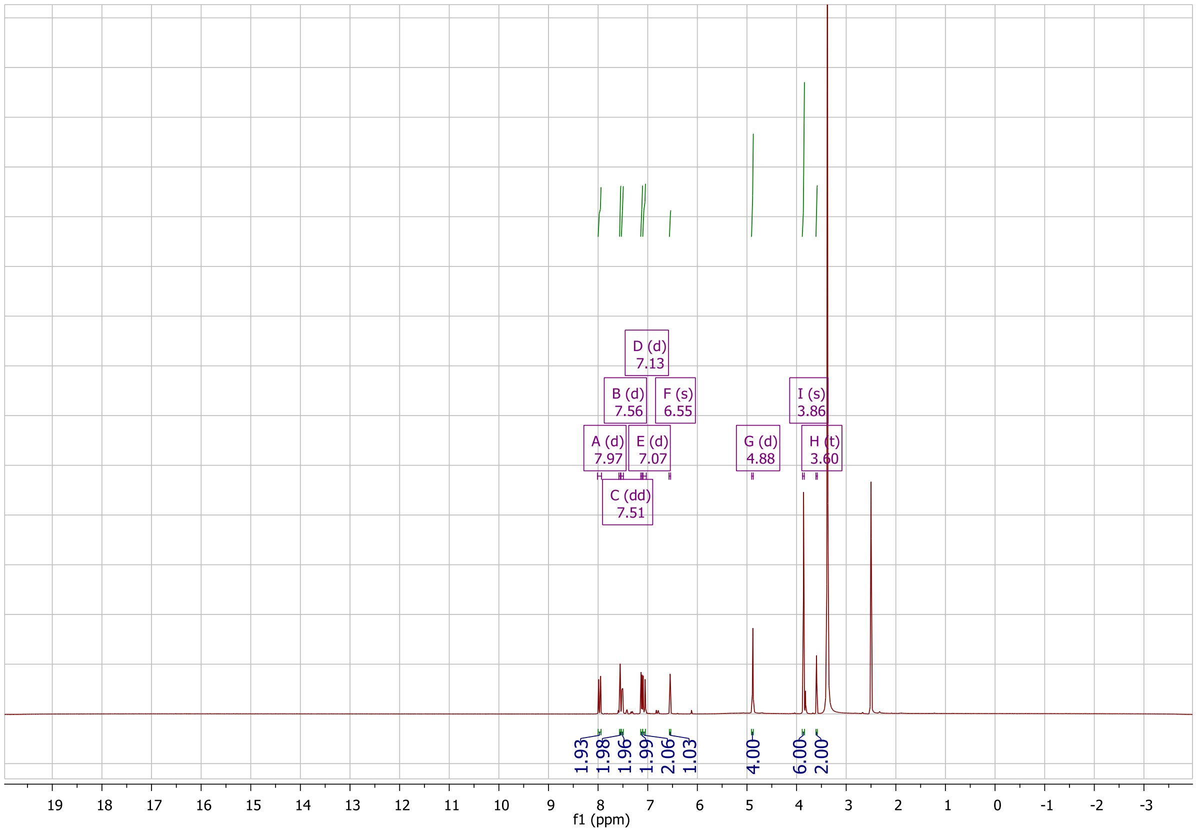

Figure S22: ^{1}H NMR spectrum (400 MHz, DMSO-d_6) of **2f**.

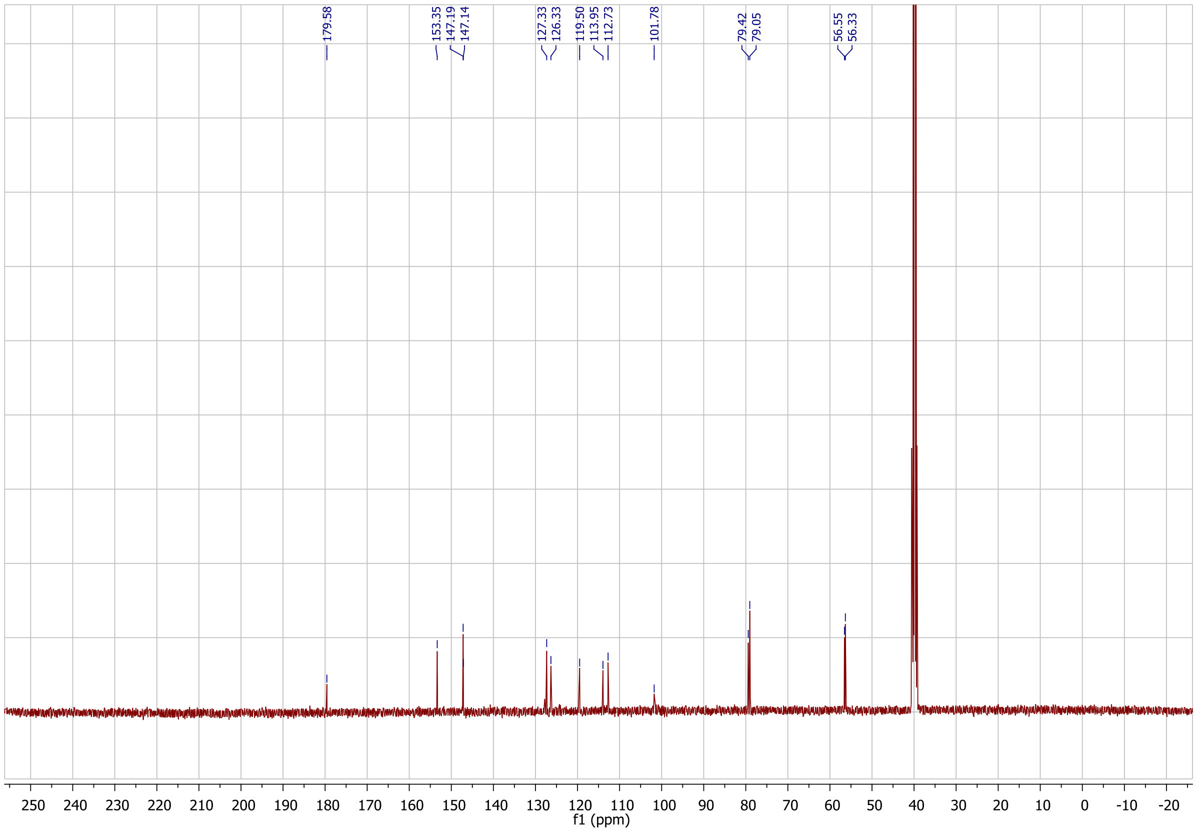

Figure S23: ^{13}C{^{1}H} NMR spectrum (101 MHz, DMSO-d_6) of **2f**.

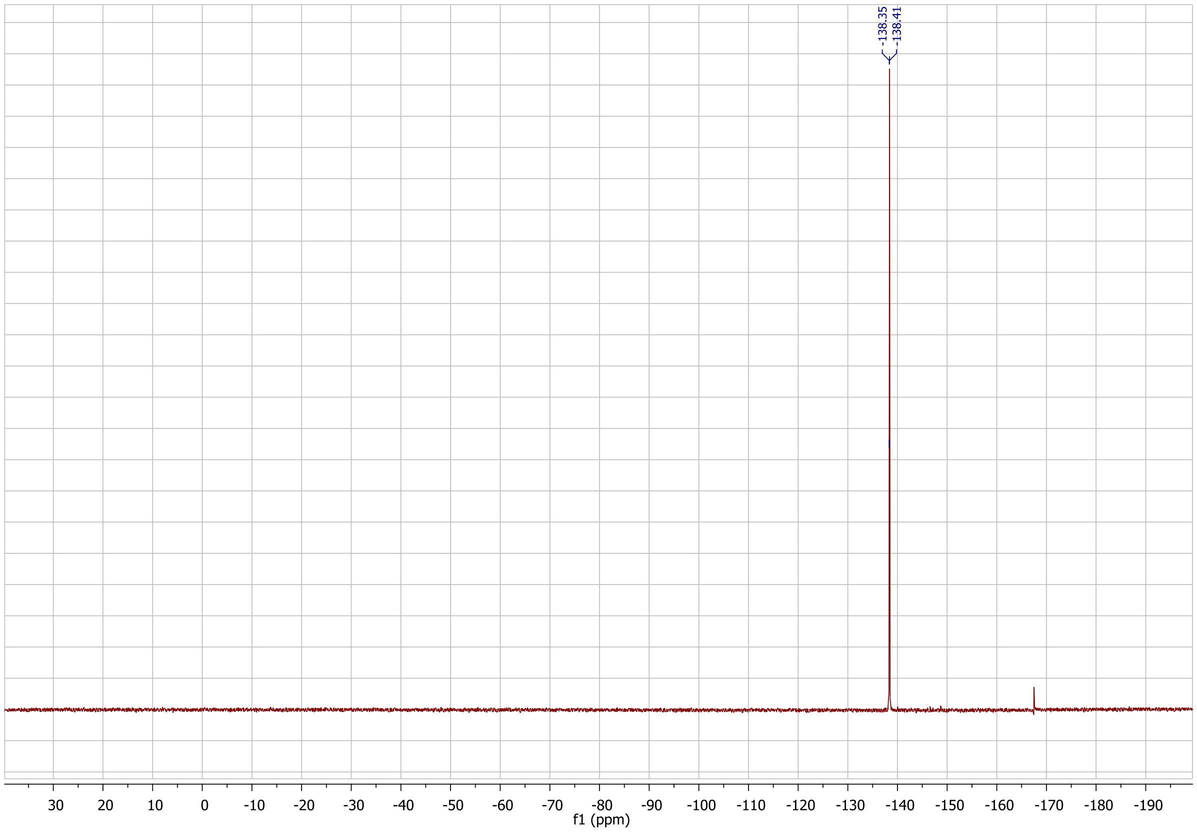

Figure S24: $^{19}F\{^{1}H\}$ NMR spectrum (188 MHz, DMSO-d_6) of **2f**.

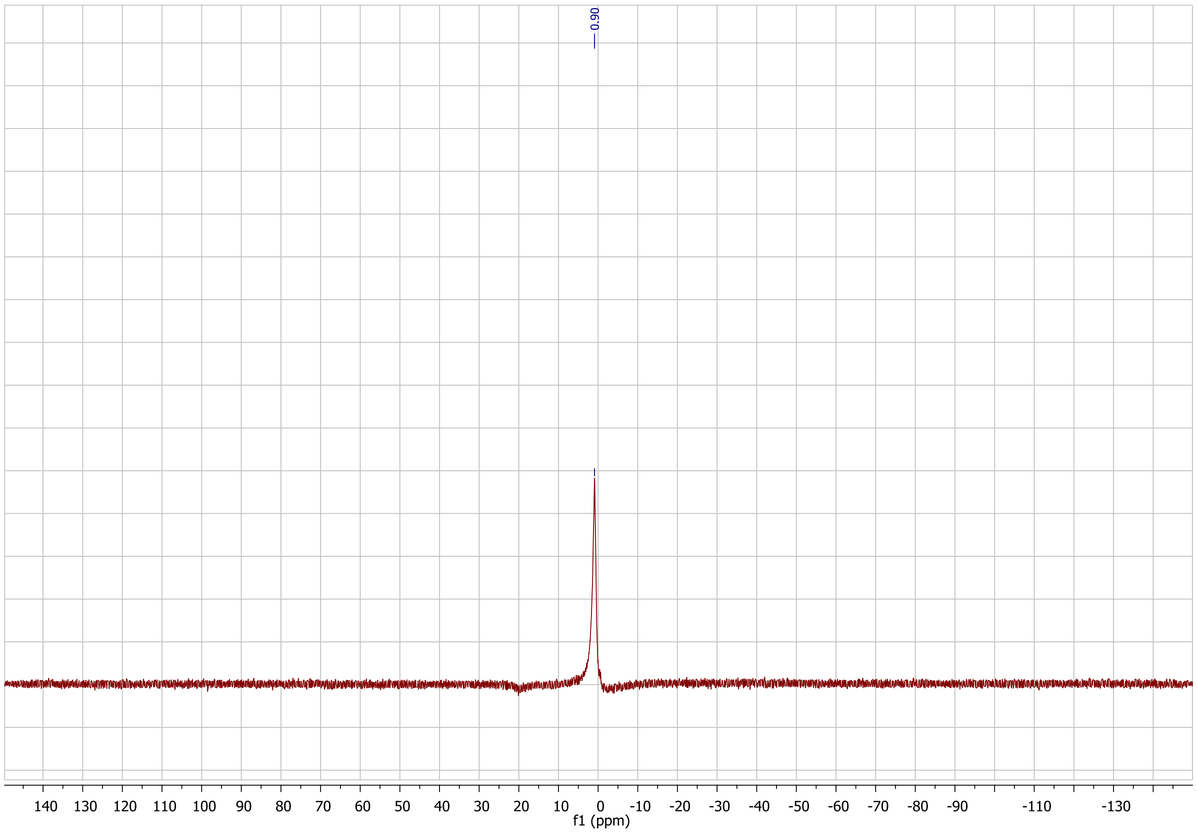

Figure S25: $^{11}B\{^{1}H\}$ NMR spectrum (128 MHz, DMSO-d_6) of **2f**.

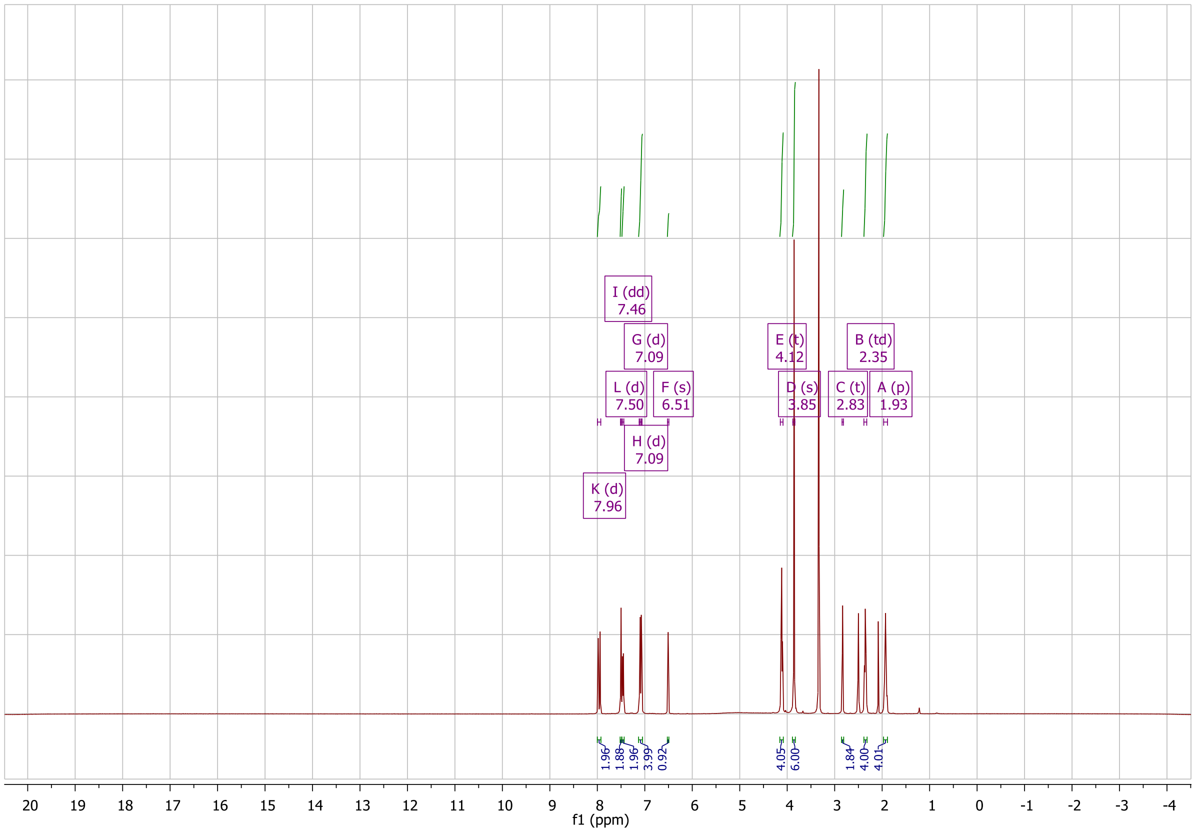

Figure S26: ^{1}H NMR spectrum 400 MHz, DMSO-d_6) of **2g**.

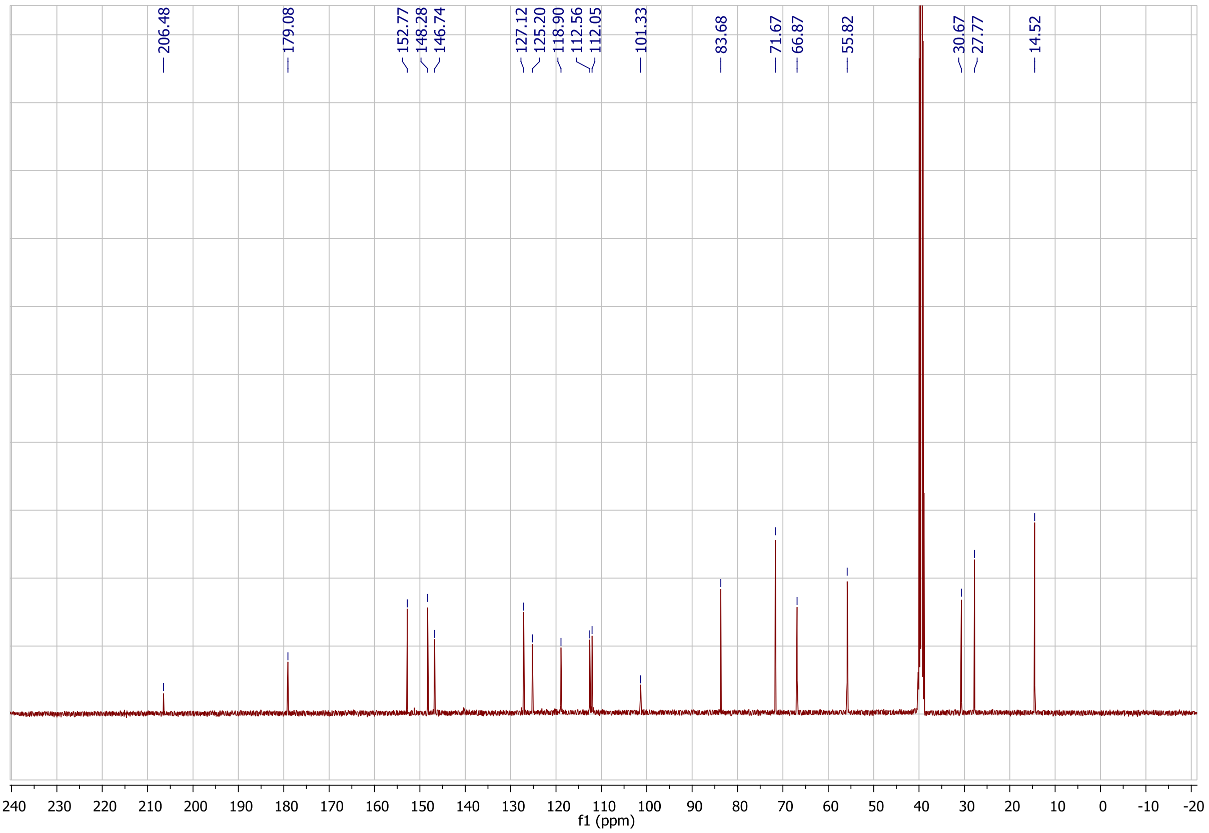

Figure S27: $^{13}C\{^1H\}$ NMR spectrum (100 MHz, DMSO-d_6) of **2g**.

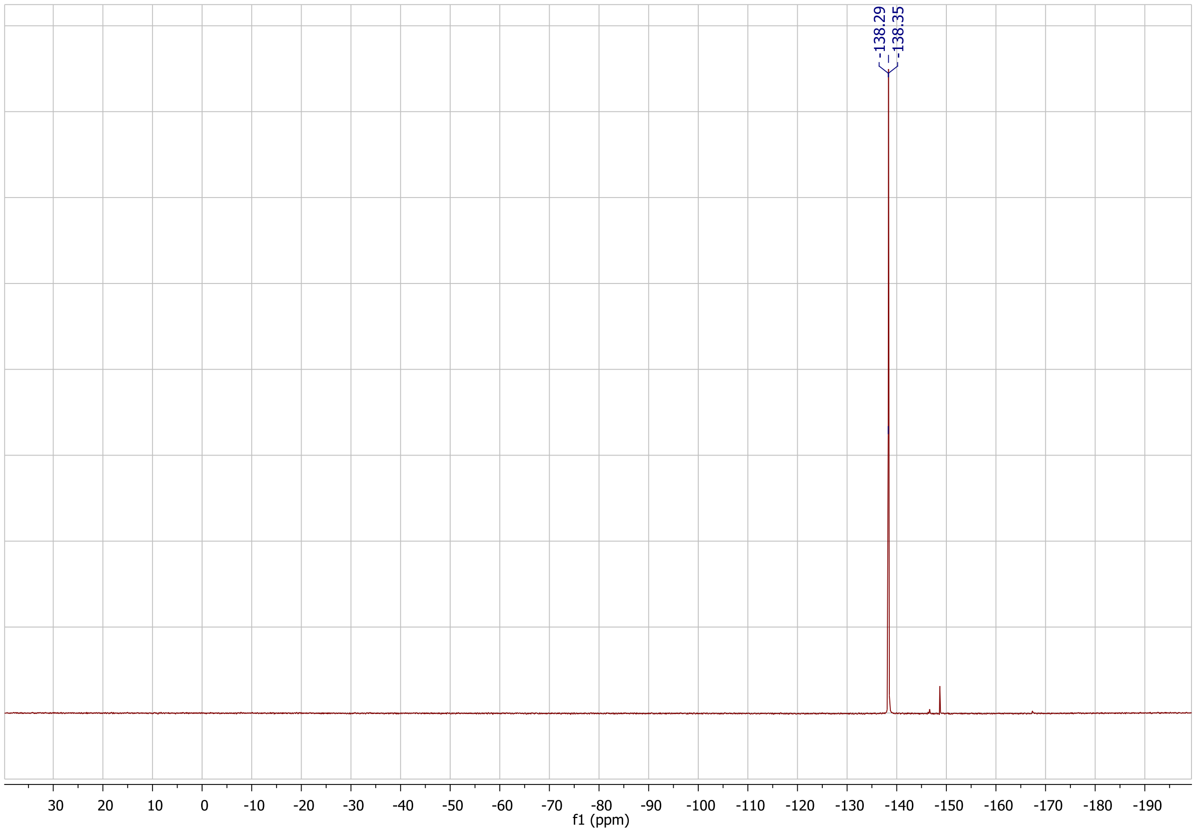

Figure S28: $^{19}F\{^{1}H\}$ NMR spectrum (188 MHz, DMSO-d_6) of **2g**.

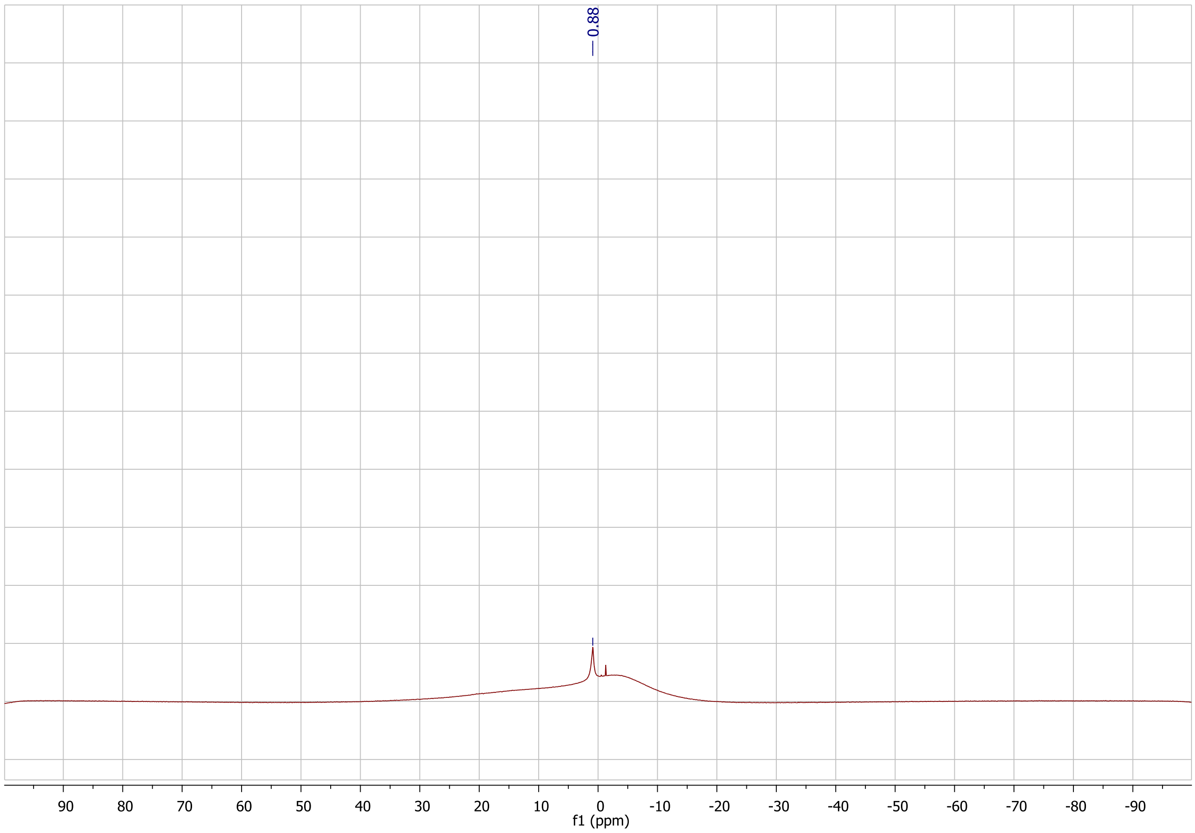

Figure S29: $^{11}B\{^{1}H\}$ NMR spectrum (128 MHz, DMSO-d_6) of **2g**.

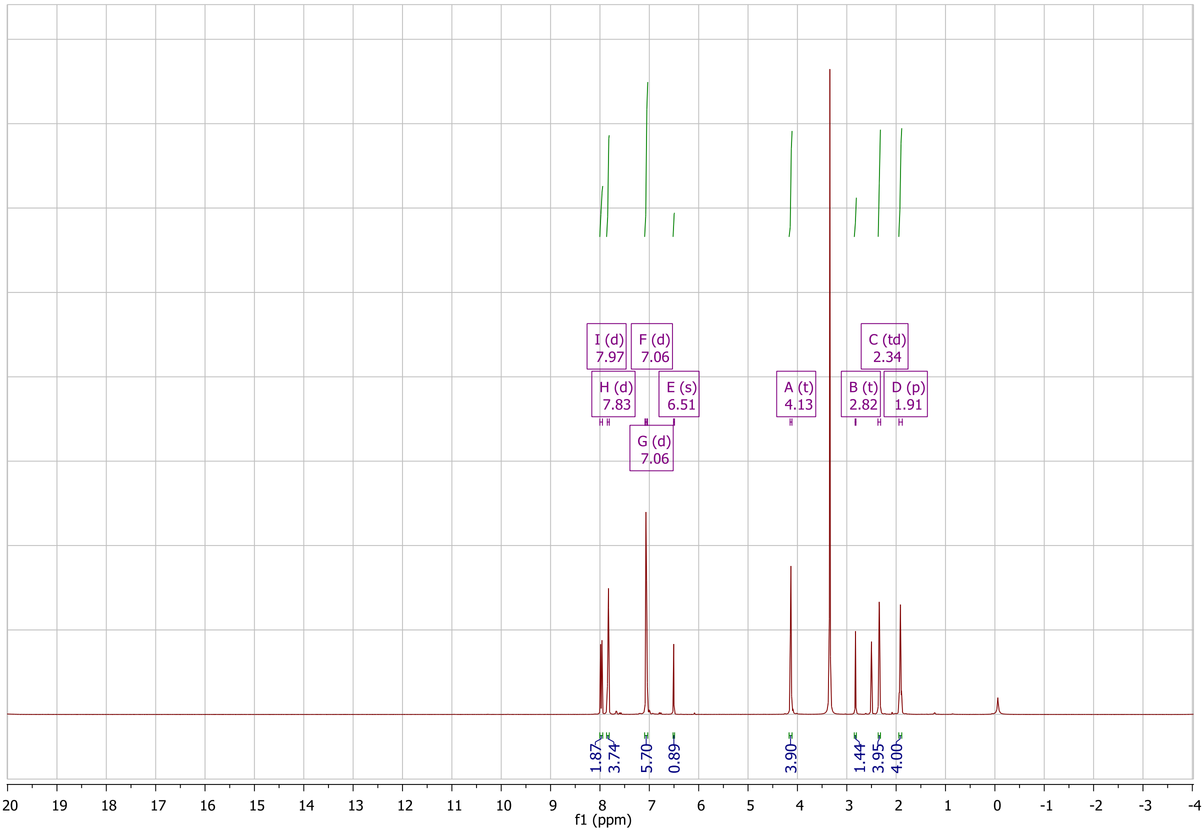

Figure S30: ^{1}H NMR spectrum (600 MHz, DMSO-d_6) of **2h**.

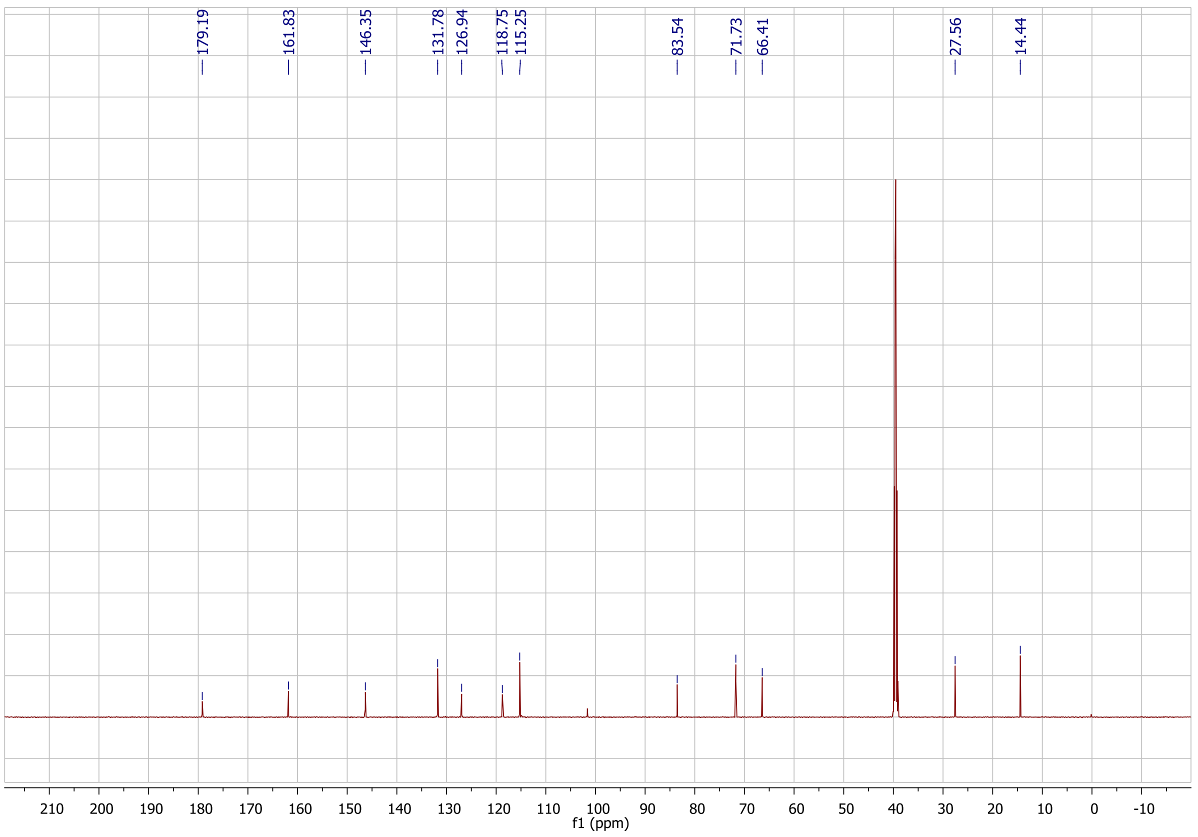

Figure S31: $^{13}C\{^{1}H\}$ NMR spectrum (150 MHz, DMSO-d_6) of **2h**.

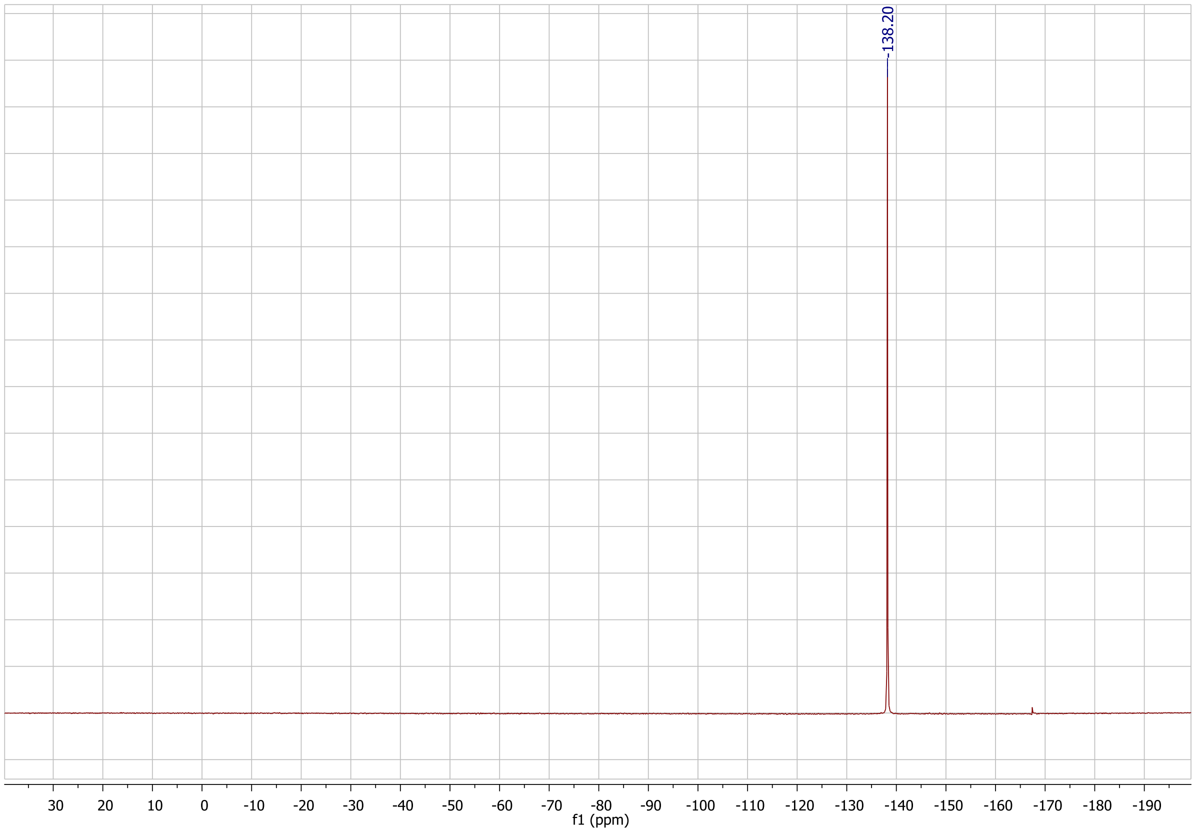

Figure S32: $^{19}F\{^{1}H\}$ NMR spectrum (188 MHz, DMSO-d_6) of **2h**.

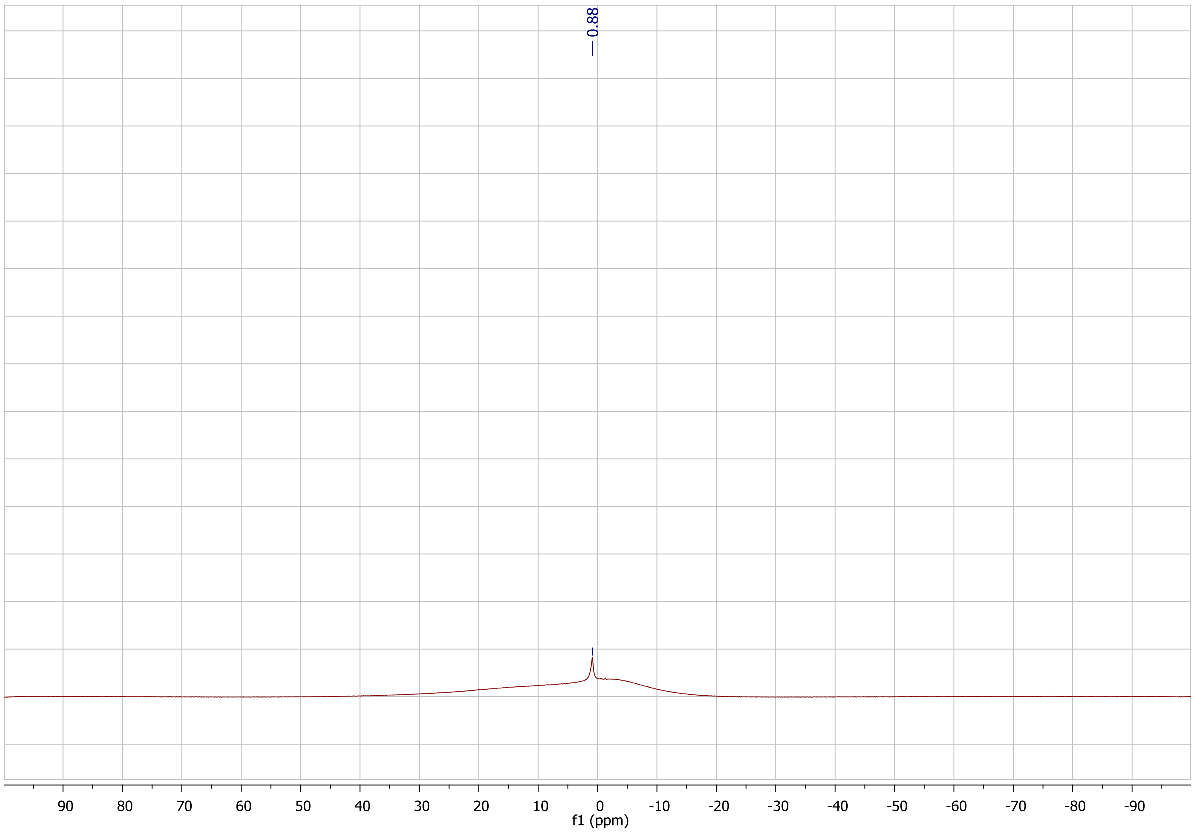

Figure S33: $^{11}B\{^{1}H\}$ NMR spectrum (128 MHz, DMSO-d_6) of **2h**.

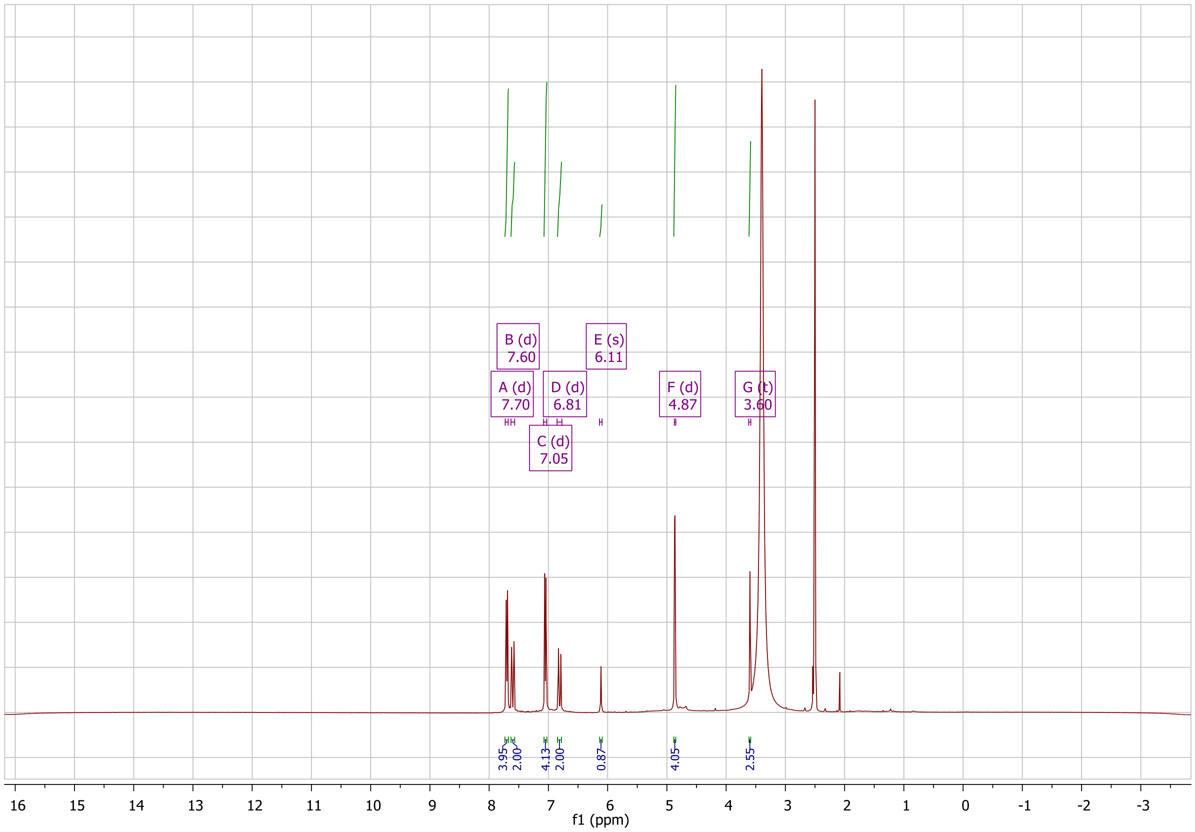

Figure S34: ^{1}H NMR spectrum (400 MHz, DMSO-d_6) of **3a**.

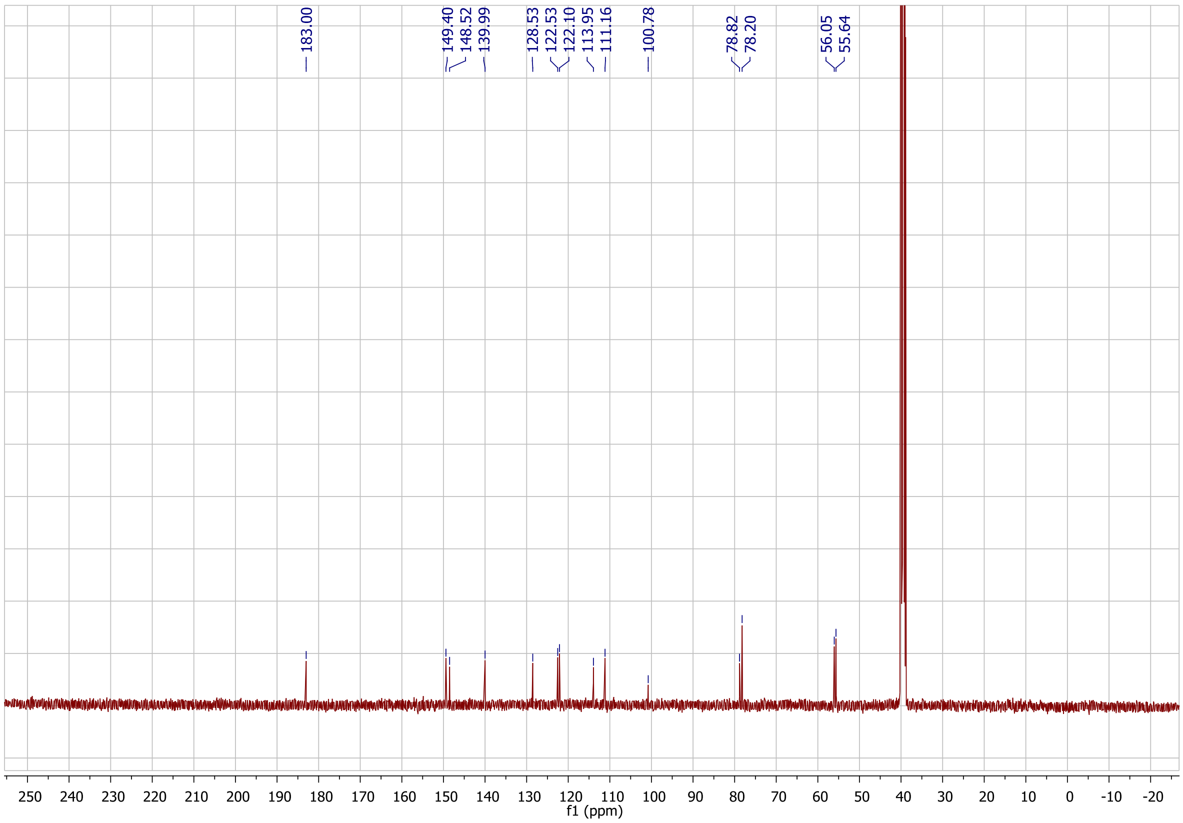

Figure S35: $^{13}C\{^{1}H\}$ NMR spectrum (100 MHz, DMSO-d_6) of **3a**.

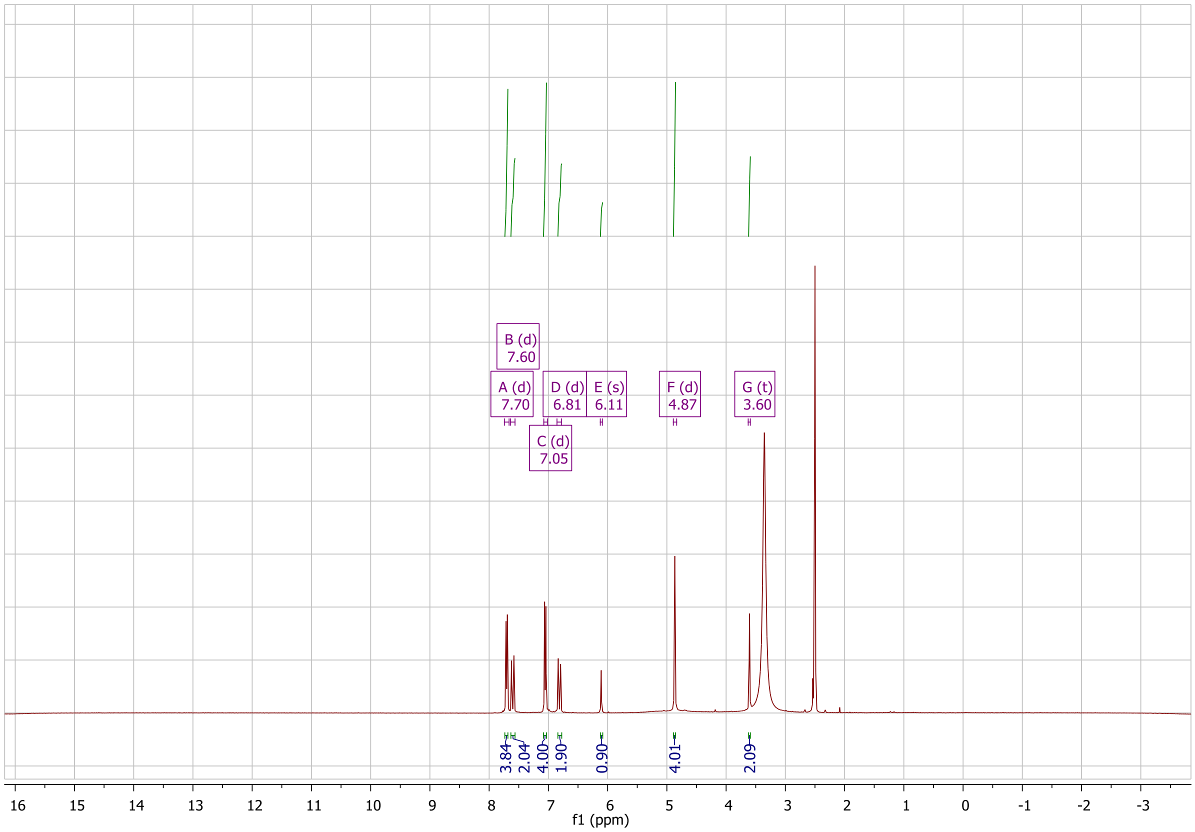

Figure S36: ^{1}H NMR spectrum (400 MHz, DMSO-d_6) of **3b**.

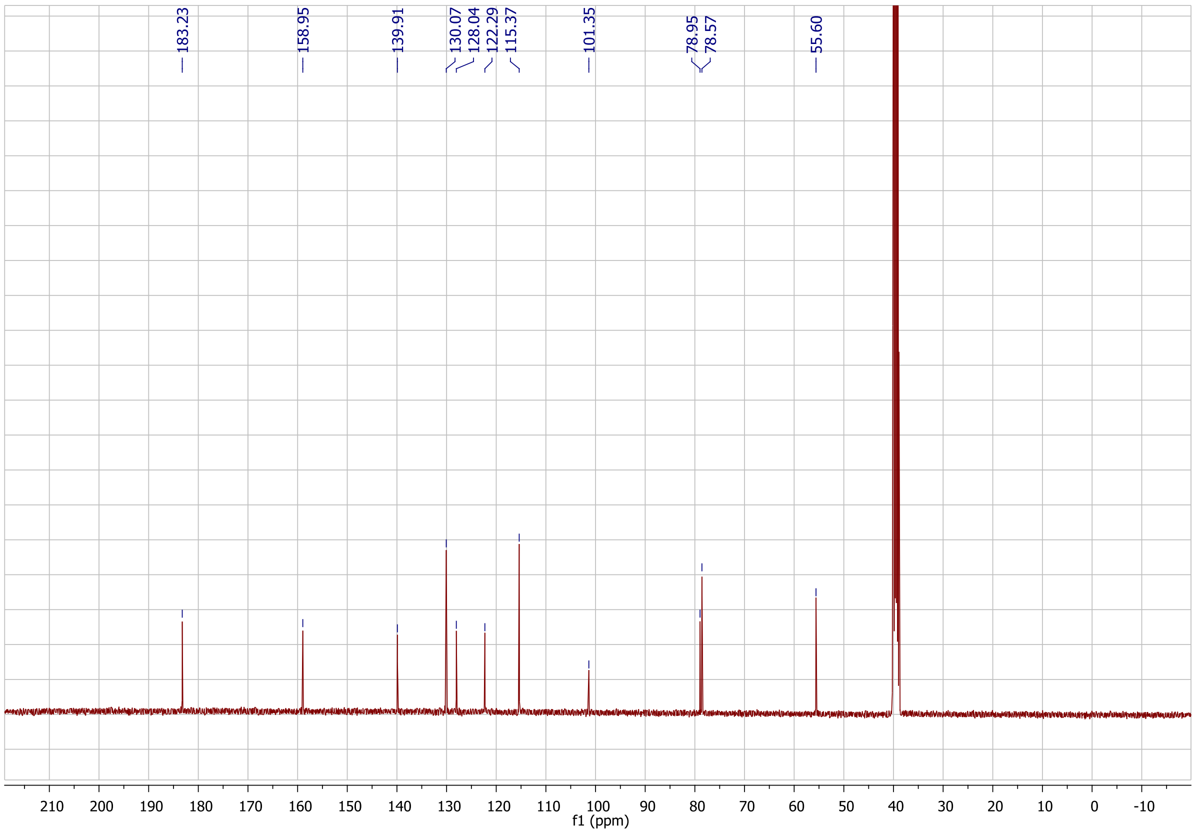

Figure S37: $^{13}C\{^{1}H\}$ NMR spectrum (100 MHz, DMSO-d_6) of **3b**.

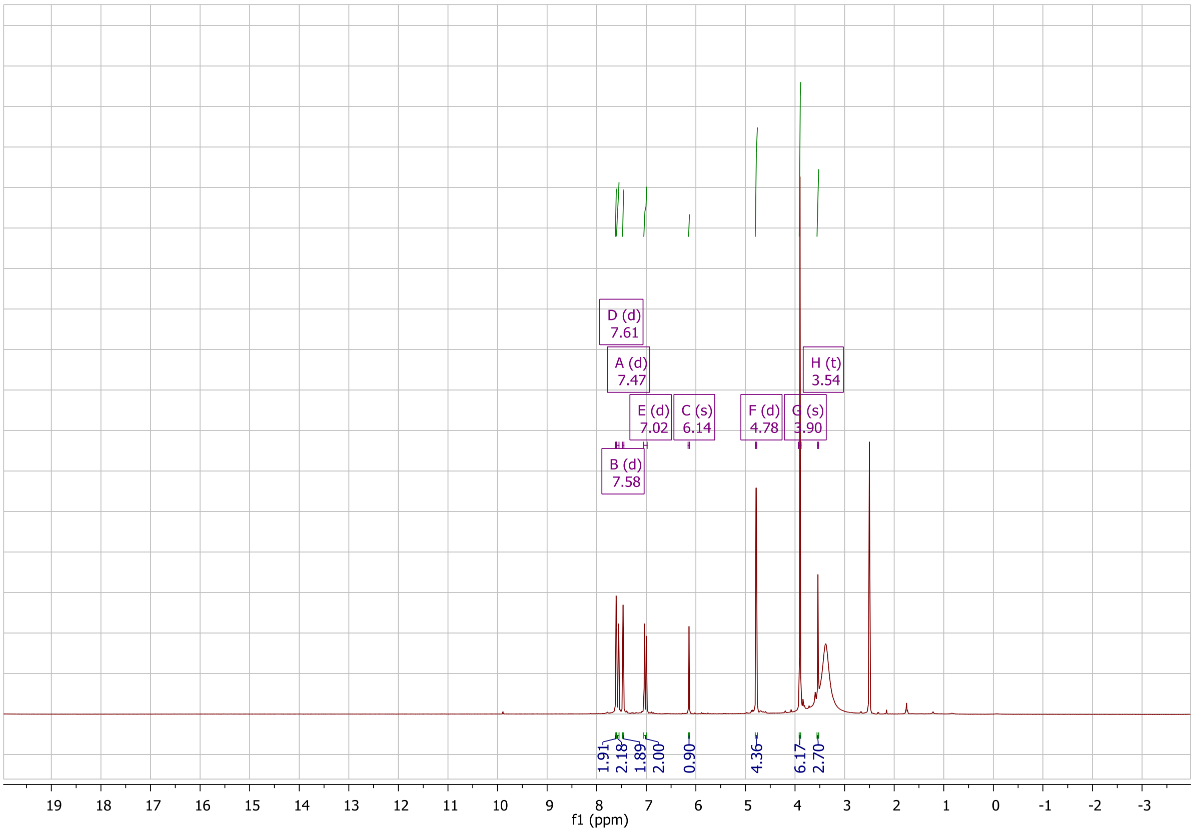

Figure S38: ^{1}H NMR spectrum (400 MHz, DMSO-d_6) of **3c**.

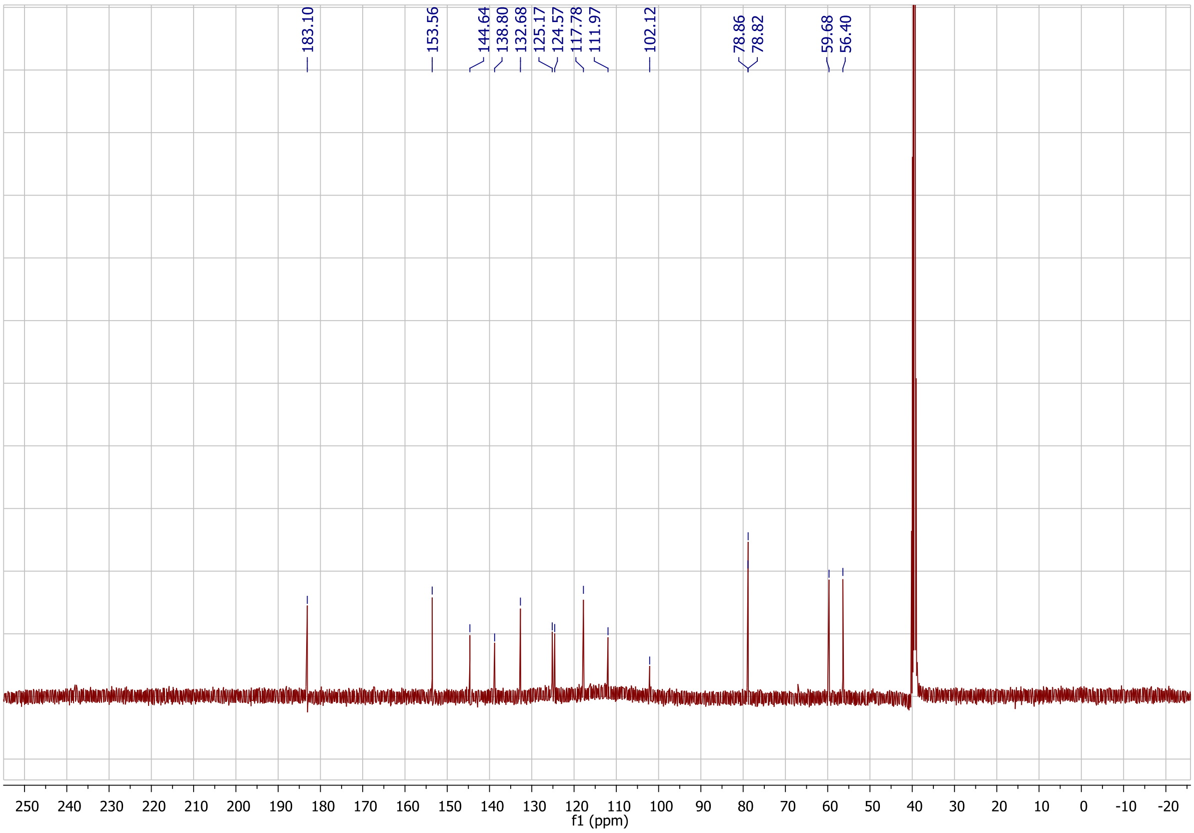

Figure S39: ^{13}C{^{1}H} NMR spectrum (100 MHz, DMSO-d_6) of **3c**.

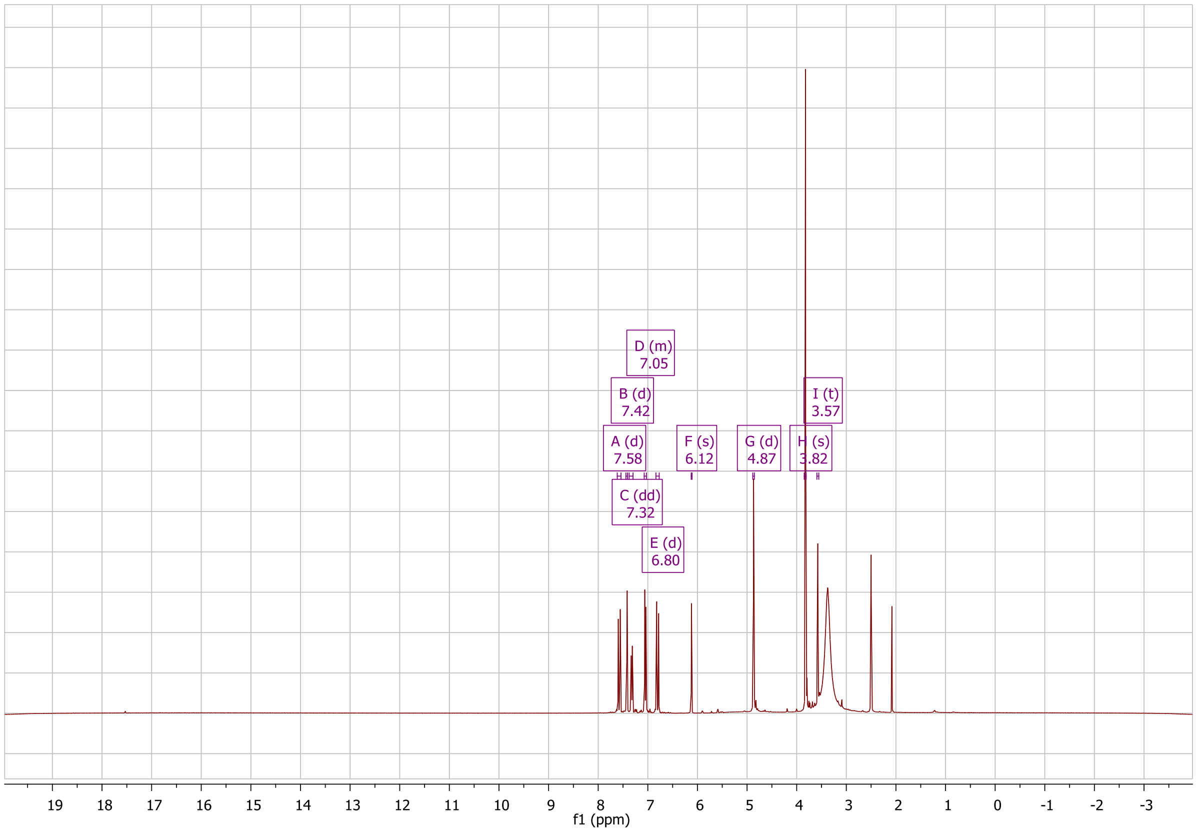

Figure S40: ^{1}H NMR spectrum (400 MHz, DMSO-d_6) of **3d**.

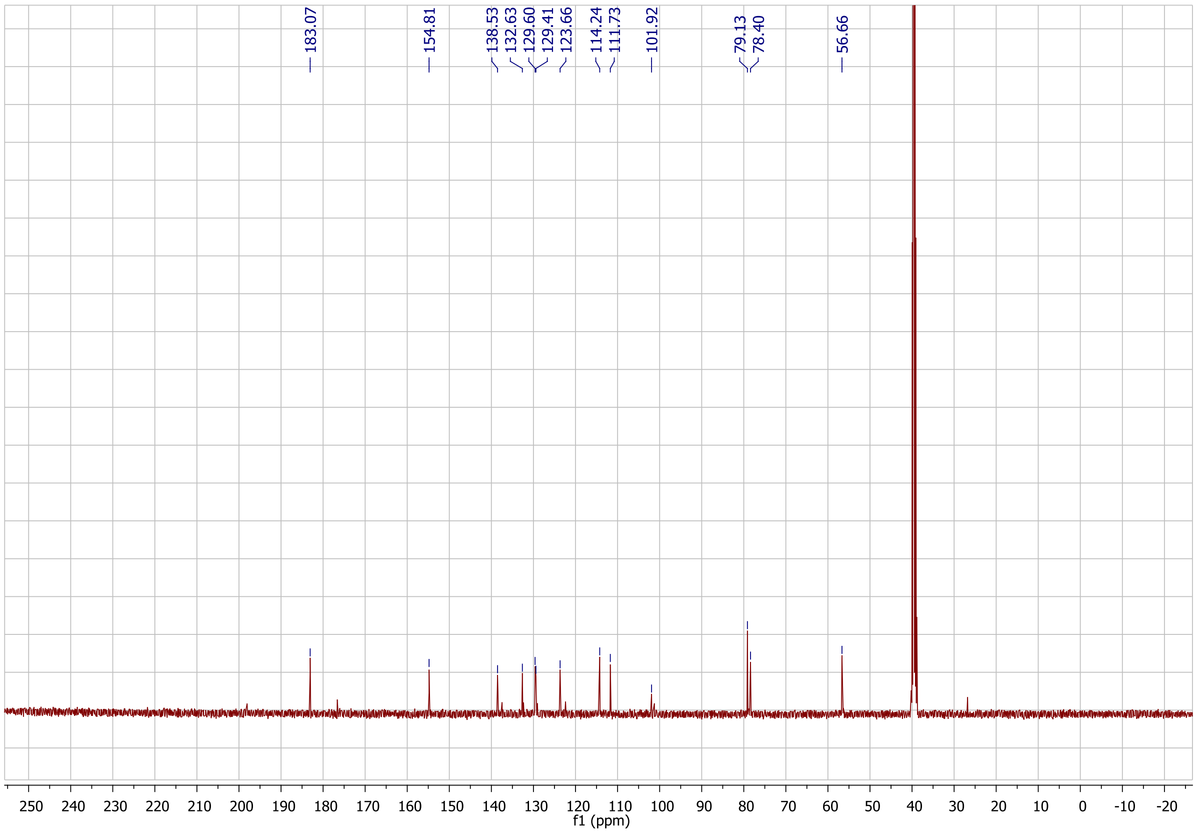

Figure S41: ^{13}C{^{1}H} NMR spectrum (100 MHz, DMSO-d_6) of **3d**.

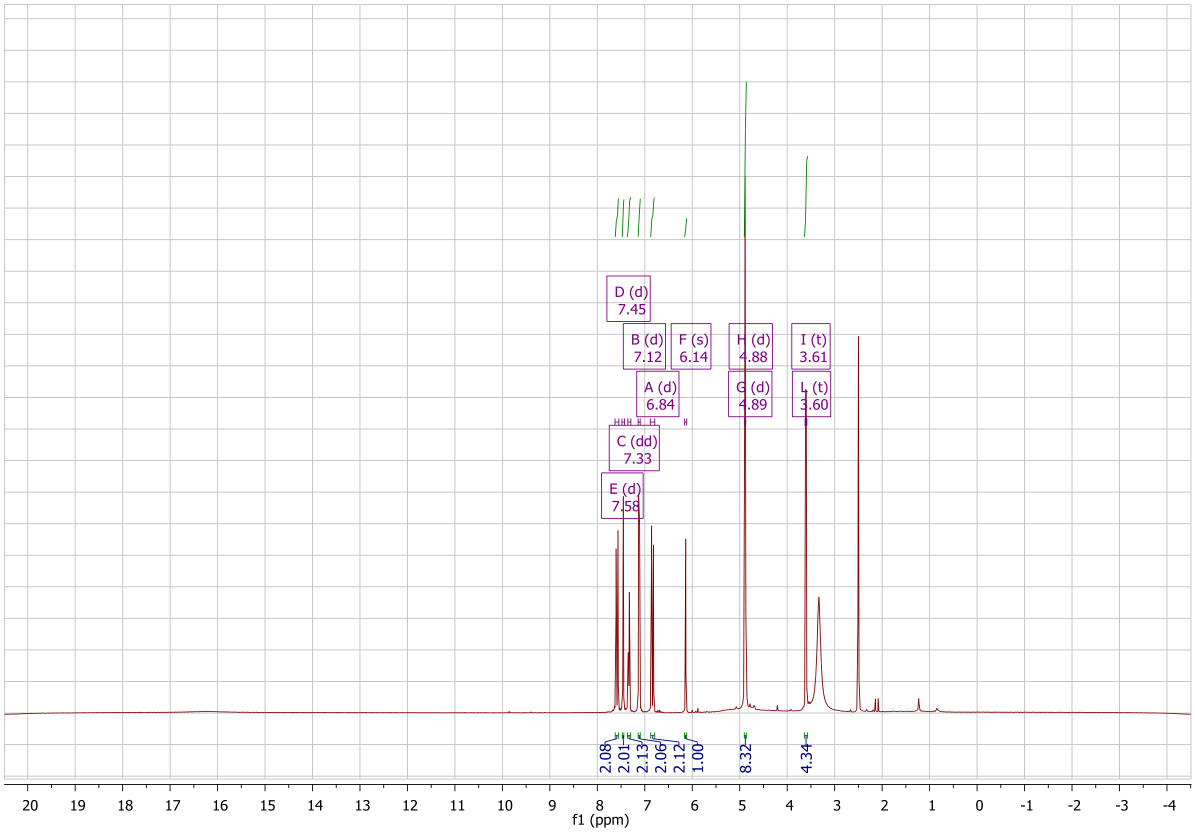

Figure S42: ^{1}H NMR spectrum (400 MHz, DMSO-d_6) of **3e**.

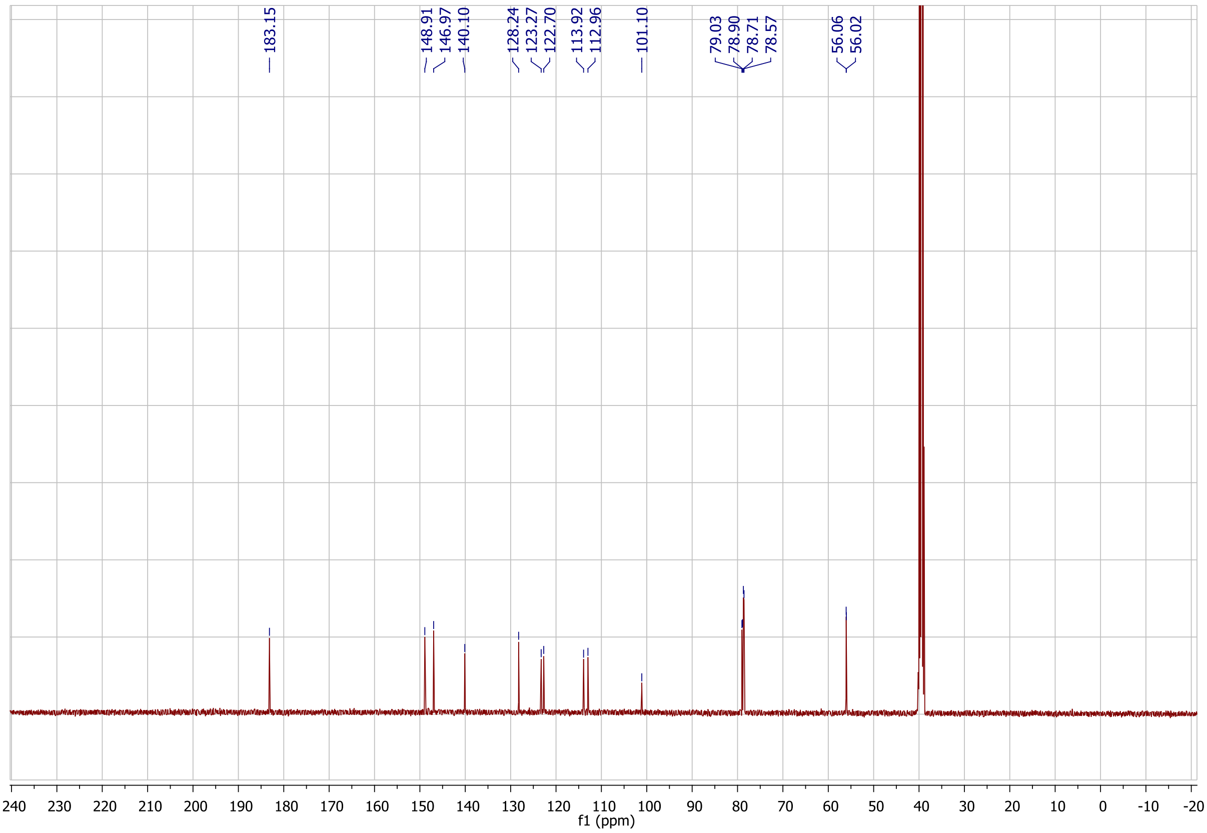

Figure S43: ^{13}C{^{1}H} NMR spectrum (100 MHz, DMSO-d_6) of **3e**.

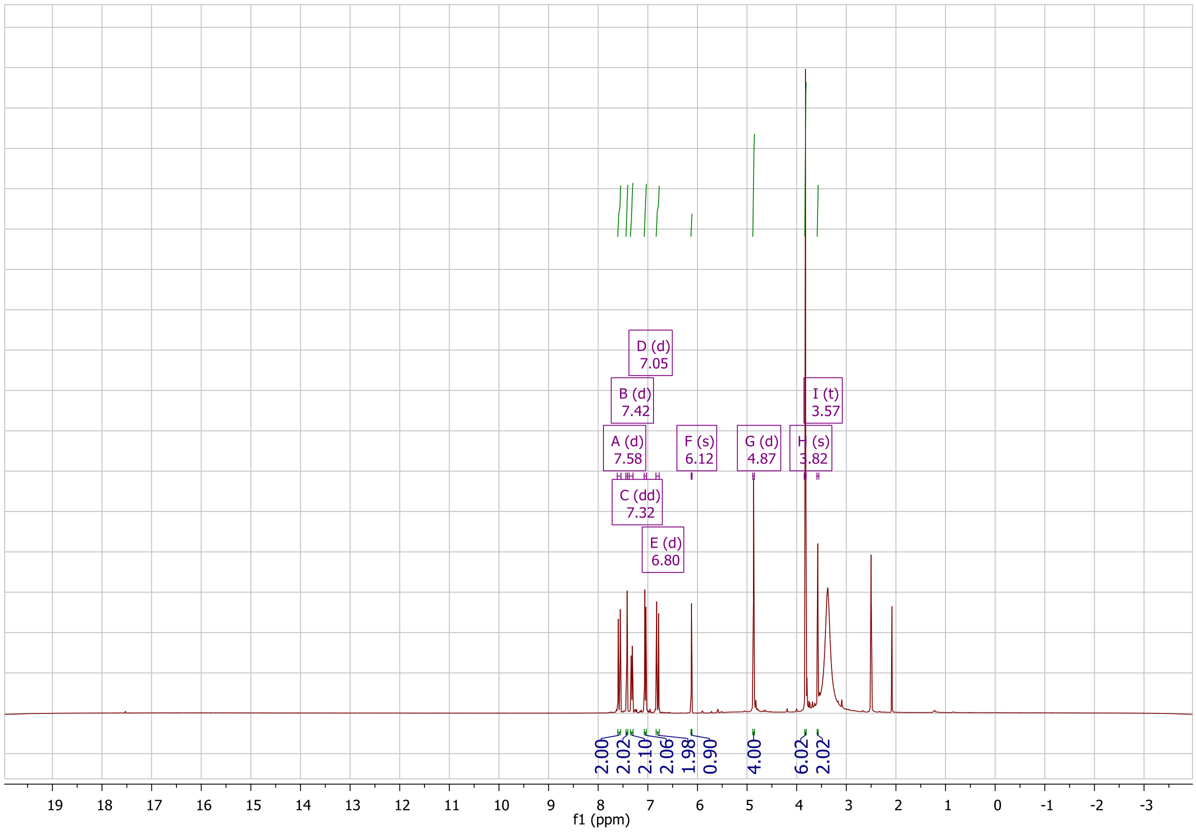

Figure S44: ^{1}H NMR spectrum (400 MHz, DMSO-d_6) of **3f**.

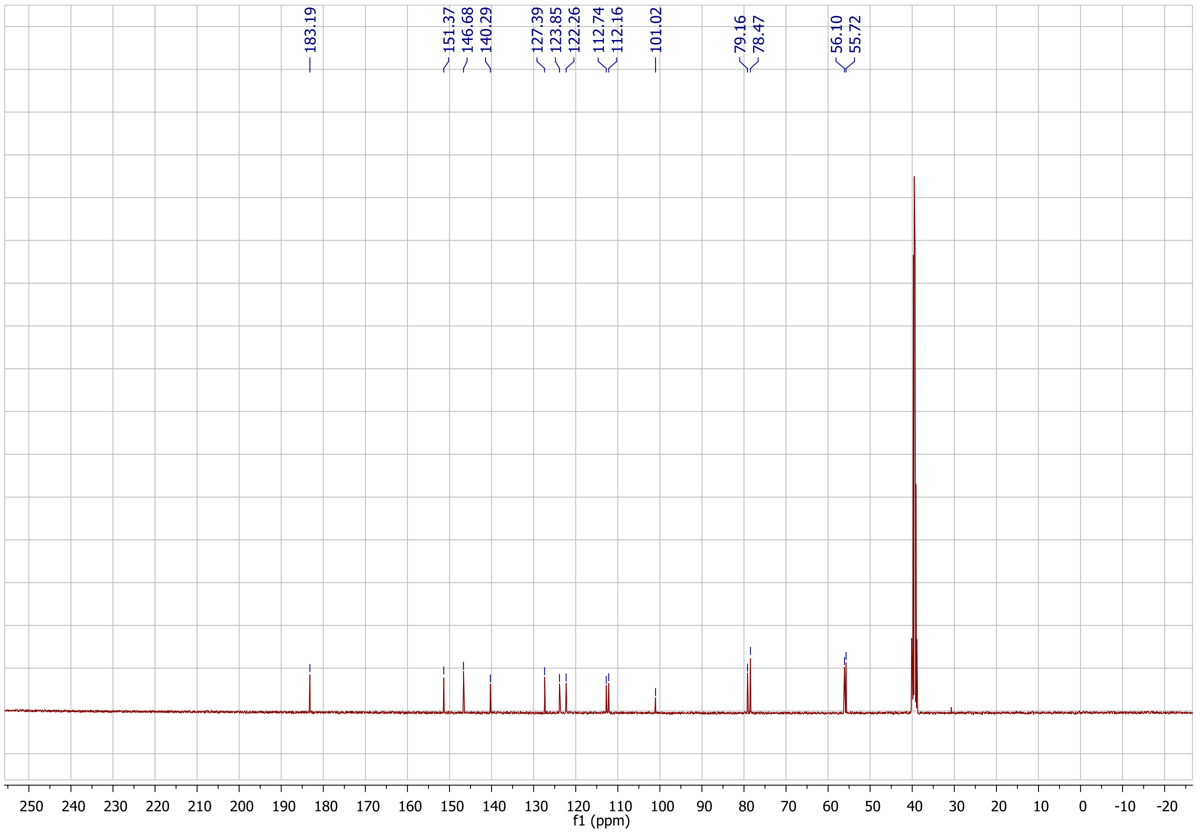

Figure S45: $^{13}C\{^{1}H\}$ NMR spectrum (100 MHz, DMSO-d_6) of **3f**.

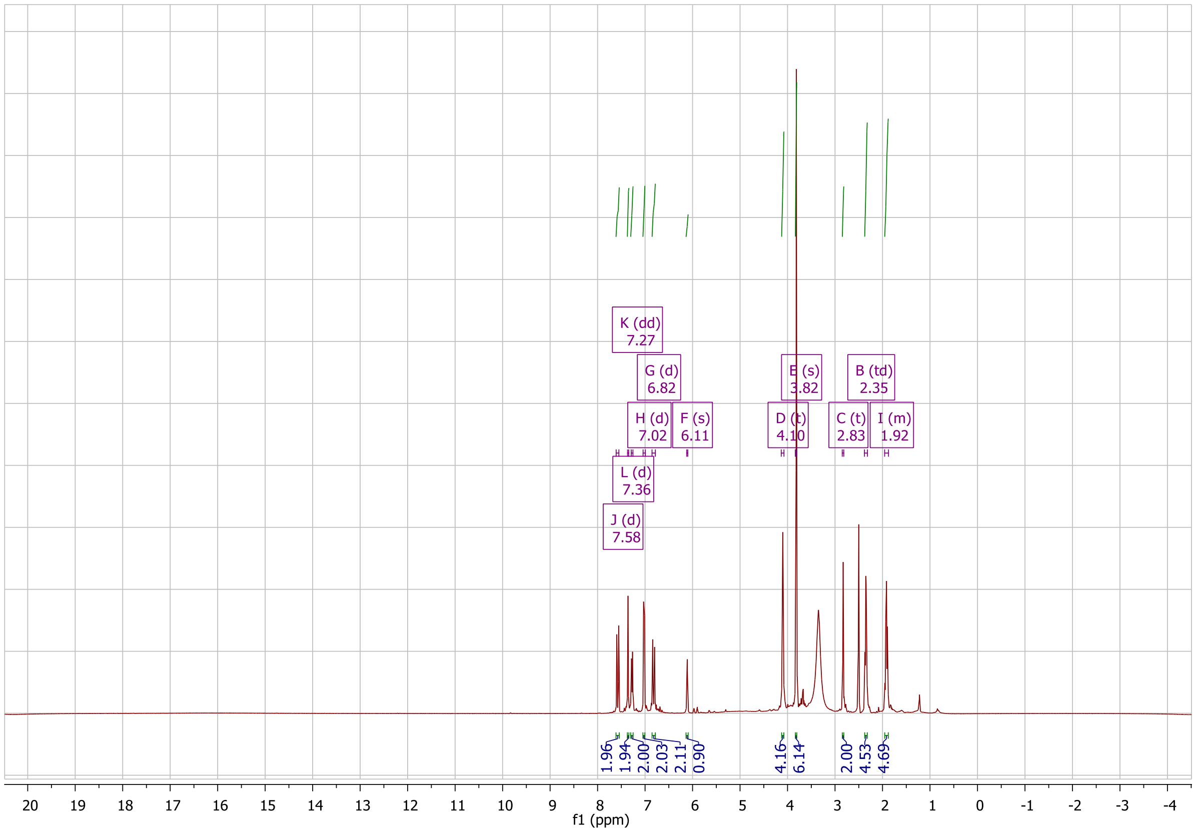

Figure S46: ^{1}H NMR spectrum (400 MHz, DMSO-d_6) of **3g**.

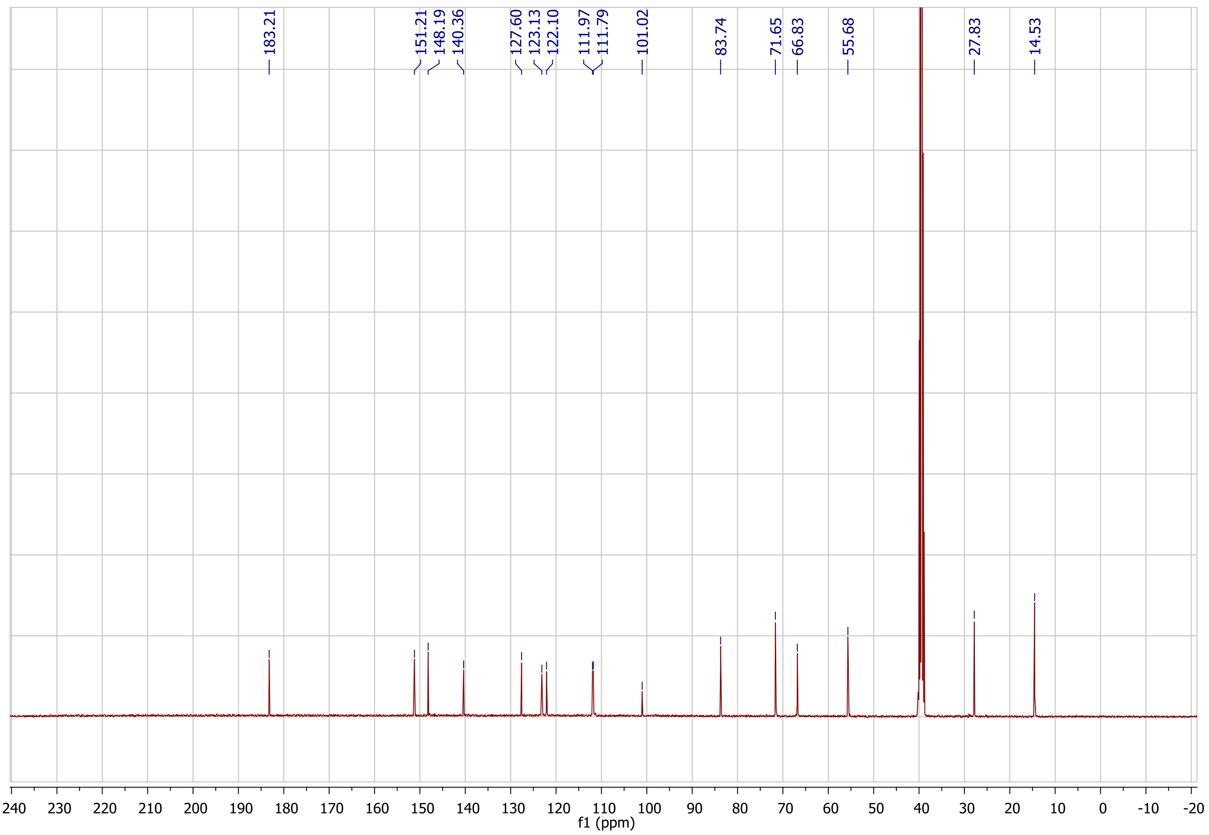

Figure S47: $^{13}C\{^{1}H\}$ NMR spectrum (100 MHz, DMSO-d_6) of **3g**.

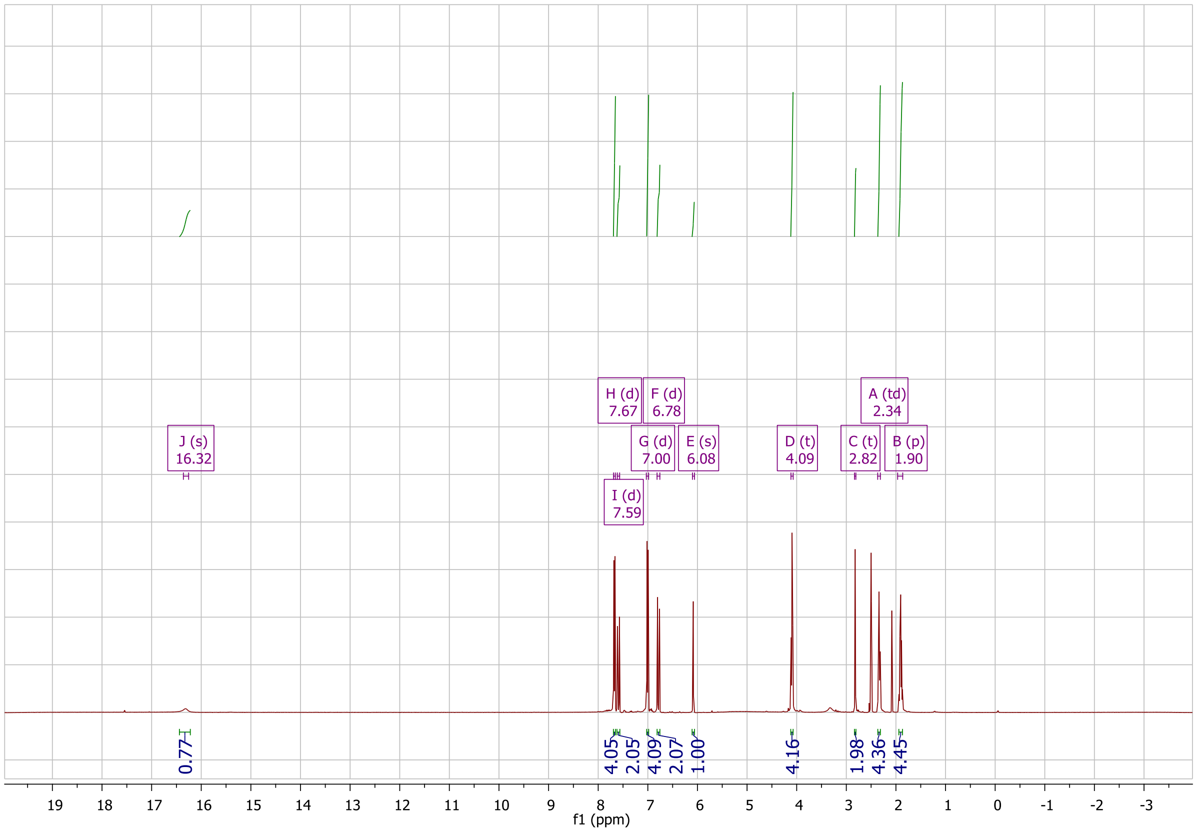

Figure S48: ^{1}H NMR spectrum (400 MHz, DMSO-d_6) of **3h**.

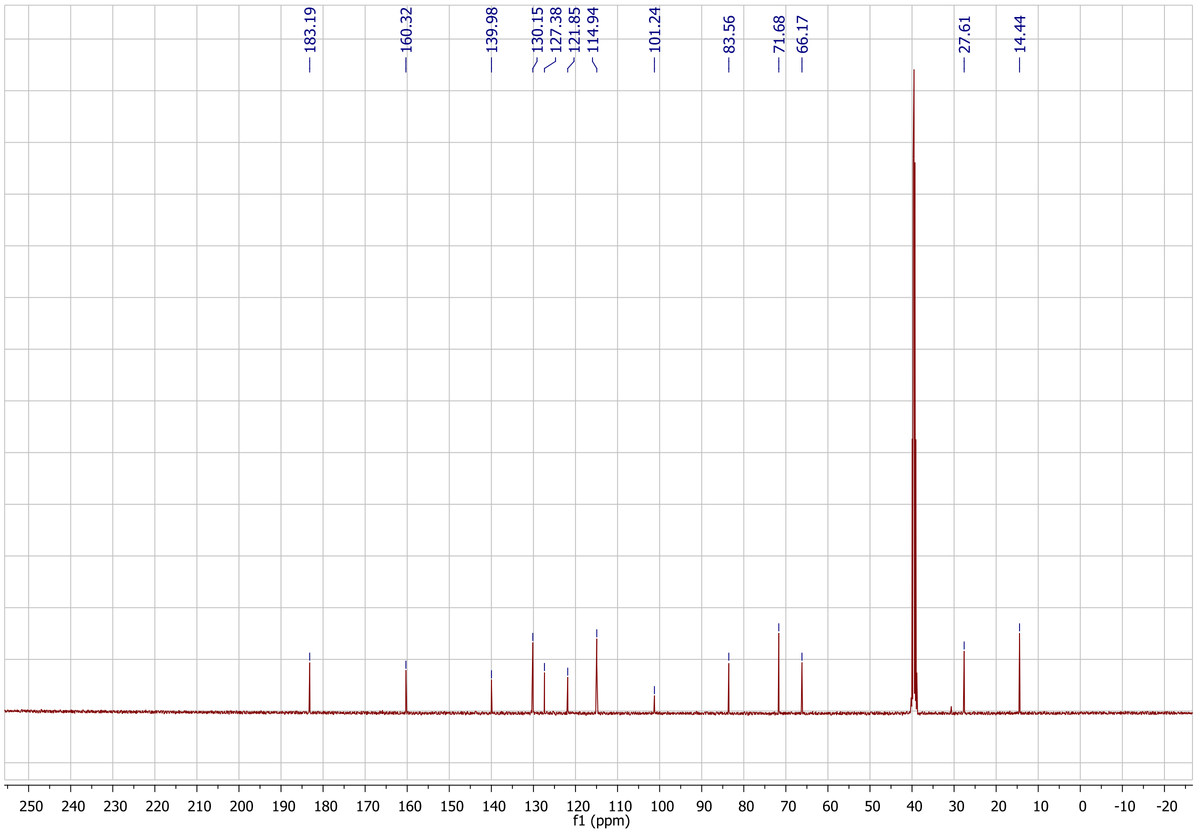

Figure S49: $^{13}C\{^{1}H\}$ NMR spectrum (100 MHz, DMSO-d_6) of **3h**.
